# Synthesis, Characterization and DFT Study of a New Family of High-Energy Compounds Based on *s*-Triazine, Carborane and Tetrazoles

**DOI:** 10.3390/molecules27217484

**Published:** 2022-11-02

**Authors:** Anton V. Makarenkov, Sergey S. Kiselev, Elena G. Kononova, Fedor M. Dolgushin, Alexander S. Peregudov, Yurii A. Borisov, Valentina A. Ol’shevskaya

**Affiliations:** 1 A.N. Nesmeyanov Institute of Organoelement Compounds, Russian Academy of Sciences, 28, bld. 1 Vavilova Street, 119334 Moscow, Russia; 2 N.S. Kurnakov Institute of General and Inorganic Chemistry, Russian Academy of Sciences, 31 Leninsky Prosp., 119071 Moscow, Russia

**Keywords:** carborane, tetrazole, HEMDs, DFT, single crystal X-ray diffraction

## Abstract

An efficient one-pot synthesis of carborane-containing high-energy compounds was developed via the exploration of carbon–halogen bond functionalization strategies in commercially available 2,4,6-trichloro-1,3,5-triazine. The synthetic pathway first included the substitution of two chlorine atoms in *s*-triazine with 5-R-tetrazoles (R = H, Me, Et) units to form disubstituted tetrazolyl 1,3,5-triazines followed by the sequential substitution of the remaining chlorine atom in 1,3,5-triazine with carborane N- or S-nucleophiles. All new compounds were characterized by IR- and NMR spectroscopy. The structure of four new compounds was confirmed by single crystal X-ray diffraction analysis. The density functional theory method (DFT B3LYP/6-311 + G*) was used to study the geometrical structures, enthalpies of formation (EOFs), energetic properties and highest occupied and lowest unoccupied molecular orbital (HOMO and LUMO) energies and the detonation properties of synthesized compounds. The DFT calculation revealed compounds processing the maximum value of the detonation velocity or the maximum value of the detonation pressure. Theoretical terahertz frequencies for potential high-energy density materials (HEDMs) were computed, which allow the opportunity for the remote detection of these compounds.

## 1. Introduction

In recent years, extensive efforts have been devoted to the development of high-energy density materials (HEDMs) such as propellants, explosives and pyrotechnics for commercial and military applications. There are many compounds that can be attributed to this category, and the analysis of their energetic characteristics provides information on the relationship between the structure and properties [1,2,3]. Among them, promising types of high-energy compounds are acyclic compounds, strained, fused and caged compounds, nitrogen heterocyclic compounds [4,5,6]. Nitrogen-containing heterocycles have traditionally been in the center of focus in the preparation of high-energy compounds due to their large positive heat of formation, high density, insensitivity and the presence of energetic N=N and C=N bonds. Upon decomposition, nitrogen-rich molecules would produce a large quantity of nitrogen gas and large amounts of energy [7,8,9,10,11].

Moreover, energetic materials based on heterocyclic compounds are considered as attractive candidates for a replacement of conventional high-energy materials, such as 2,4,6-trinitrotoluene (TNT), 1,3,5-trimethylene-2,4,6-trinitramine (RDX), and 1,3,5,7-tetranitrotetraaza-cyclooctane (HMX) [12,13]. Earlier works showed that 1,3,5-triazine is an important framework for the construction of numerous energetic compounds, owing to its positive heat of formation, high nitrogen content (53.8%), and thermal stability. Moreover, their nitrogen content can be increased by substitution with suitable functional groups. Chemically modified 1,3,5-triazine with a variety of nitrogen-containing substituents have been demonstrated to be promising HEDMs [14,15,16]. The key compound for their preparation is cyanuric chloride, which can be readily functionalized through the conventional S_N_Ar reactions with various nucleophiles including a variety of energetic moieties [17]. Indeed, the replacement of chlorine atoms in cyanuric chloride by tetrazole moieties offers the possibility of tuning the energetic properties in order to produce nitrogen-rich HEDMs with improved energetic properties [18]. Tetrazole is an important functionality of the most of energetic materials due to 80% nitrogen content, stability, and high enthalpy of formation. The enthalpies of formation of tetrazole in the crystalline and gaseous states are ΔH_f_(cr) = 236.1 kJ/mol (56.4 kcal/mol) and ΔH_f_(g) = 327.2 kJ/mol (78 kcal/mol), respectively [19,20]. Tetrazoles are also characterized by high potential energy and low sensitivity to friction and shock and are thus used as green energetic materials in propellants, gas generators, explosives and pyrotechnics. Tetrazoles have some advantages compared to classical high-energy materials such as lower toxicity, easier synthesis, being cleaner and environmentally friendly decomposition [21].

Among the variety of HEDMs, boron-containing materials have also drawn particular attention because of boron’s high heat of combustion on both a gravimetric (58 kJ/g) and volumetric (136 kJ/cm^3^) basis [22,23,24]. Amorphous boron of more than 99% purity is added to propellant compositions as a fuel additive and burn rate modifier [22]. However, the application of pure boron powder in the solid propellant composition has some disadvantages such as the difficulty in ignition and incomplete combustion in the presence of atmospheric oxygen [25,26]. New types of boron materials for energetic composition were prepared to resolve this problem. It is believed [27] that the use of boron in a composition with aluminum can increase the combustion efficiency of boron due to a synergistic effect of composite systems including Al and B as well as the capping of boron nanoparticles with a polymer can provide a solution to the aforementioned problem. The use of an energetic polymer for that purpose can bring additional heat close to the surface of the nanoparticles and facilitate their ignition [27].

However, nowadays studies in the field of boron HEDMs have been directed towards the preparation of the monomolecular systems featuring high heats of formation and high nitrogen content based on tetraazido-, azole and azolium borate salts [28,29,30]. Potential candidates for the development of new boron energetic materials with both high energy and low sensitivity are nitrogen-rich carborane systems [31,32], the functionalization strategies of which have been recently described. The *closo*-carboranes (C_2_B_10_H_12_) are boron-rich icosahedral compounds with unique structural and electronic properties, remarkable thermal and chemical stability, spherical geometry, and exceptional hydrophobic character [33]. They are characterized by the existence of three-center two-electron bonds, and can be considered as three-dimensional delocalized aromatic systems [34,35], demonstrating many features with that of benzene. The remarkable thermal and chemical stabilities make them unique candidates for use in several special applications in the fields of materials science [36,37] including HEDMs since they demonstrate a high enthalpy (ΔH_f_ = 42 kcal/mol) of formation [38]. Previously, it was shown by us that carboranyl-substituted tetrazoles are prospective for their potential applications as HEDMs [31,32]. In this work, aiming to achieve promising energy-rich materials with carborane exploration, the synthetic route for three-component high energy systems containing *s*-triazine, carborane and tetrazoles within one molecule was been developed. Spectroscopic, structural data and DFT calculations for new compounds are reported.

## 2. Result and Discussion

### 2.1. Synthesis

**Scheme 1:**
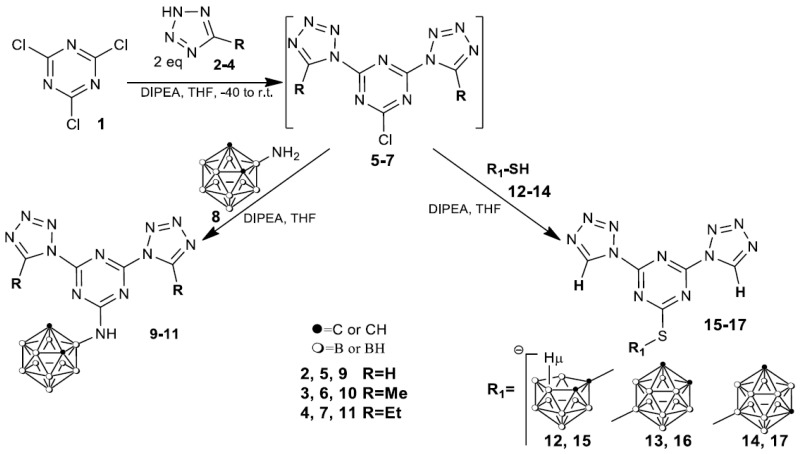
Reactions of intermediates **5**–**7** with amino- and mercaptocarboranes.

Our initial goal was to develop an efficient strategy for the preparation of nitrogen-rich carborane-containing HEDMs. 2,4,6-Trichloro-1,3,5-triazine, i.e., cyanuric chloride (**1**), was suggested as the trifunctional rigid linker for the synthesis of desired compounds since it is a relatively stable nitrogen heteroaromatic system, promising for derivatization with various nucleophiles during S_N_Ar reaction capable of forming a variety of highly structured polyfunctionalized intermediates [39]. The synthetic pathway to obtain new *s*-triazine derivatives modified with tetrazoles and carborane cluster is shown in Scheme 1.

Tetrazoles **2**–**4** were reacted with cyanuric chloride **1** (molar ratio 2:1) at −40 °C to ambient temperature for 2–24 h in THF in the presence of DIPEA as an HCl scavenger demonstrating, by TLC (CHCl_3_-acetone 10:1), the formation of 2-chloro-4,6-ditetrazolyl-1,3,5-triazines intermediates **5**–**7**. It should be noted that the formation of disubstituted compounds **5**–**7** depends on the structure of tetrazoles **2**–**4** and under similar reaction conditions, a decrease in the reactivity and a significant increase in the reaction time in series **2** (2–3 h) ≥ **3** (6–7 h) ≥ **4** (24 h) were observed. The sequential substitution of the remaining electrophilic site in **5**–**7** with the carborane cluster was carried out by treating the reaction mixture with 3-amino-*o*-carborane **8** [40] and heating at 20–25 °C for 20–24 h to afford the desired compounds **9**–**11** in 59–84% isolated yields as white solids.

At the same time, the reactivity of intermediate **5** towards isomeric mercapto carboranes **12**–**14** in which mercapto group is bound to electron-deficient **12** [41] or electron-donating **13**, **14** carborane groups [42] was studied in order to clarify the effect of the carborane structure on the energetic characteristics of the synthesized compounds. The reaction of **5** with carboranes **12**–**14** readily proceeded in THF in the presence of DIPEA for 3 h at ambient temperature to afford compounds **15**–**17** in 50–79% yields. It was found that the reaction of carborane **12** with **5** is accompanied by the deboronation of a *closo*-carborane cluster to afford anionic *nido*-derivative **15**, obtained as cesium salt.

**Scheme 2:**
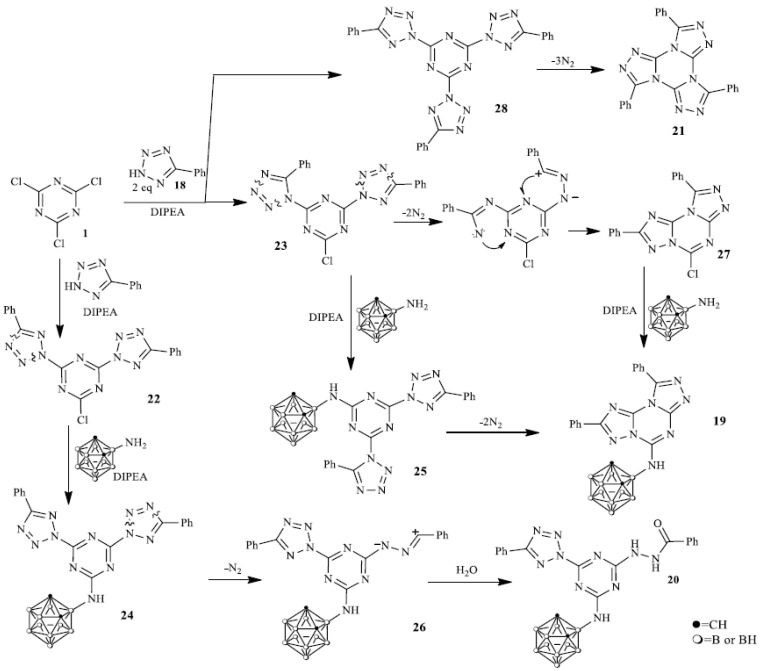
Reaction of phenyltetrazole **18** with aminocarborane **8**.

A similar reaction of phenyltetrazole **18** with cyanuric chloride **1** and aminocarborane **8** led to the formation of a complex mixture of products from which three substances were isolated and characterized by X-ray diffraction analysis (Scheme 2), namely 5-(*o*-carborane-3-yl)amino-2,9-diphenylbis [1,2,4]tri-azolo[1,5-a:1′,5′-c][1,3,5]triazine **19**, 2-(2-benzoylhydrazinyl)-4-(*o*-carborane-3-yl)amino- 6-(5-phenyl-2*H*-tetrazol-2-yl)-1,3,5-triazine **20** and 3,7,11-triphenyltris([1,2,4]triazolo) [4,3-a:4′,3′-c:4′’,3′’-e] [1,3,5]triazine **21**.

**Figure 1:**
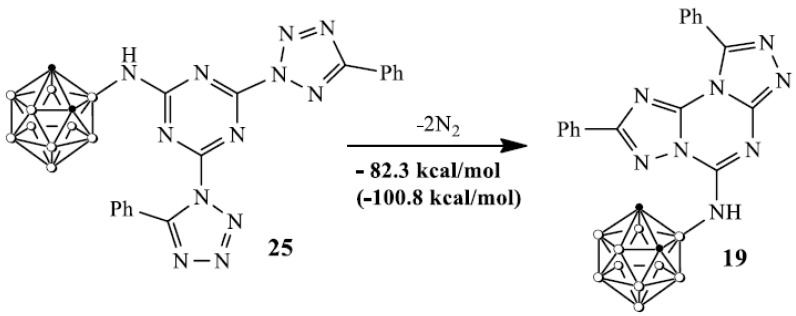
Values of enthalpy and a change in the Gibbs free energy in the process of transformation of **25** into **19**.

A very concise discussion of the key players in this area and their contribution may also be placed here. The reaction of the tetrazole **18** and cyanuric chloride **1** led to the formation of tetrazole N^1^- and N^2^-substituted [43,44,45,46] *s*-triazine regioisomers **22**, **23** and their reaction with carborane **8** resulted in the formation of unstable intermediates **24**, **25**. The evolution of nitrogen in compound **25** afforded fused carborane triazolotriazine **19** according to the known rearrangement [47] while **24** was received via the interaction of the intermediate 1,3-dipole **26** with H_2_O [48] used for the reaction treatment. Trimer **21** could be formed via a self-cyclization of the symmetrical tris(tetrazolyl)triazine **28** [47,49,50]. The low thermal stability of intermediates **22**–**25** is in good agreement with the DFT calculations. DFT calculations (Figure 1) for compound **25** showed that this compound can be easily transformed into a stable compound **19** via a successive evolution of nitrogen molecules from two tetrazole rings in **25**. This process can be characterized by a negative value of the enthalpy of the process and a change in the Gibbs free energy (in parentheses) in the form of the following the equation:

Apparently, the ease of the decomposition of complex **25** with the release of two nitrogen molecules is due to the relatively small activation energy of this process. Simple energy estimation confirms this conclusion. The comparison of similar reactions for compounds **9** and **10**, where R = H and Me, showed that the activation energies for R = Ph are approximately 20 kcal lower than for compounds **9** and **10**.

### 2.2. NMR Spectral Data of Prepared Compounds

All the prepared compounds are stable in air and could be stored for extended periods of time.

In the ^1^H NMR spectra, tetrazole CH protons in compound **9** (Figure S1) appeared downfield at δ = 10.1 ppm, protons of the tetrazole methyl groups in compound **10** (Figure S9) were observed at δ = 3.0 ppm and protons of the tetrazole ethyl groups in compound **11** (Figure S11) were observed at δ = 3.5 ppm (CH_2_) and δ = 1.4 ppm (CH_3_), respectively. The protons of B^3^-NH amino groups in all synthesized compounds were observed as broad singlets at δ = 9.0–9.1 ppm. The signals of the carborane CH protons in compounds **9**–**11** shifted downfield relatively to the corresponding signals in amine **8** due to the electron-withdrawal effect of the *s*-triazine ring and manifested as broad singlets at δ = 5.3 ppm. The ^11^B NMR spectra for compounds **9**–**11** are also in good agreement with the structures of the synthesized compounds and are in the range from δ = −4.5 to −14.5 ppm, supporting the *closo*-structure of carborane polyhedron. The signals of the substituted B^3^-NH atoms appeared as singlets in the region of δ = −6.0–−6.5 ppm. In ^1^H NMR spectra, the signals of tetrazole CH protons in *s*-triazines **15**–**17** were observed as singlets at δ = 8.95–10.17 ppm, as in the case of compounds **9**–**11**, which indicates the absence of a significant influence of the carborane electronic properties on the tetrazole ring. The signal of the carborane CH protons for compound **17** (Figure S21) was observed as a broadened singlet at δ = 3.98 ppm, whereas in the case of compounds **15** and **16**, carborane CH protons appeared as two broadened singlets at δ = 3.16, 3.69 ppm for **15** (Figure S17) and at δ = 4.84, 4.90 ppm for **16** (Figure S19). The ^11^B NMR signals of compounds **16** and **17** are in a range from δ = 1.8 to −14.4 ppm for **16** and from −4.4 to −17.5 ppm for **17** and the signals of substituted boron atoms in position 9 were observed as singlets at δ = 1.8 ppm and −4.4 ppm for compounds **16** (Figure S20) and **17** (Figure S22), respectively. For *nido*-derivative **15** (Figure S17), a characteristic extra hydrogen atom signal was observed in the ^1^H NMR spectrum at δ = −1.9 ppm in the form of a broadened doublet, and in the ^11^B NMR spectrum, a characteristic doublet of doublet signal was observed at δ = −34.4 ppm (Figure S18), attributed to the boron atom in position 10 of *nido*-carborane polyhedron.

**Figure 2:**
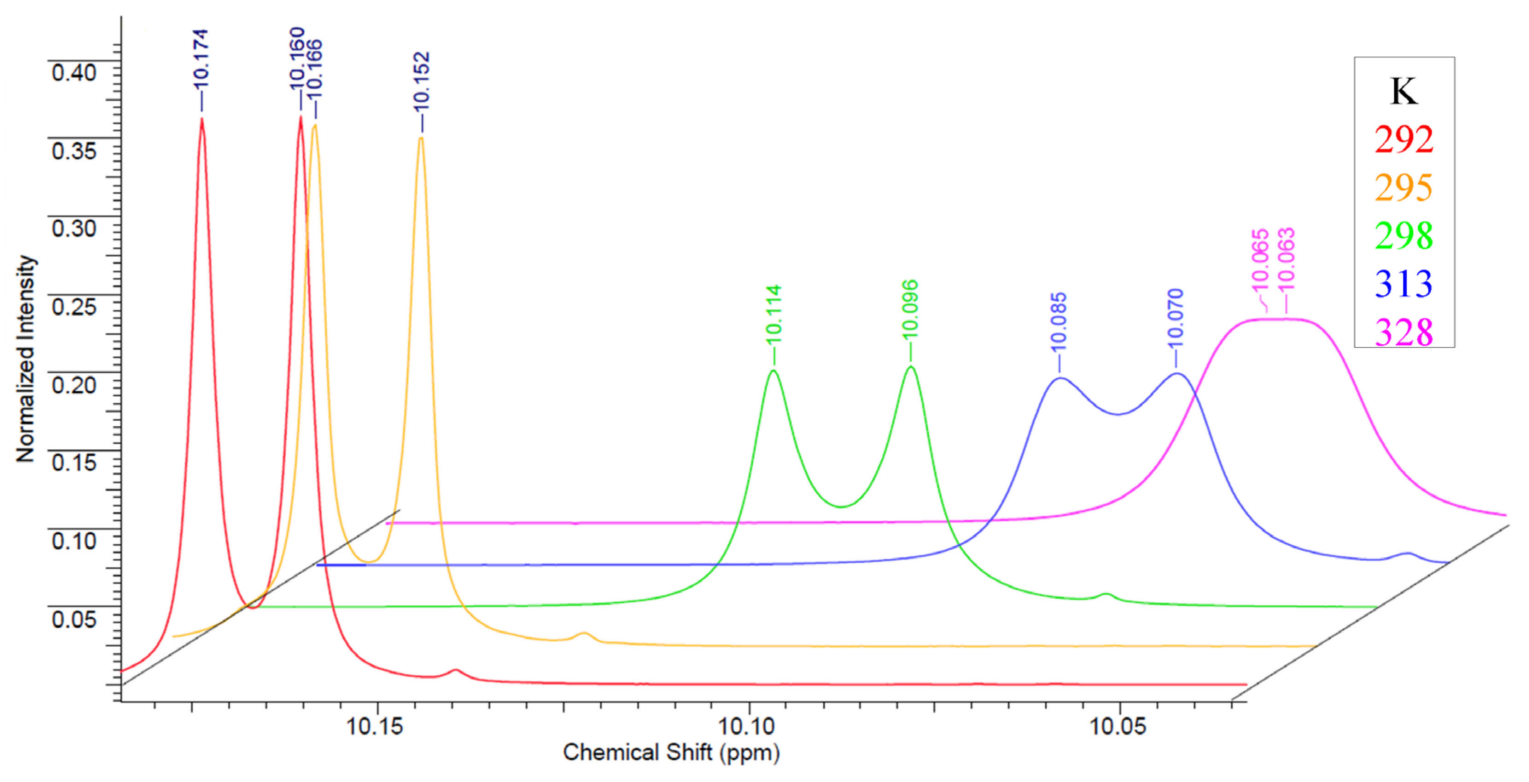
^1^H NMR spectra of compound **9** recorded in acetone-D_6_ at different temperatures.

Nevertheless, it should be noted that special features for compounds **9**–**11** were observed in their NMR spectra. In the NMR spectra of 5-alkyl (**10**, **11**) and 5-H (**9**) carborane-substituted bis(tetrazolyl)-*s*-triazines prepared using 3-amino-*o*-carborane **8**, a splitting of signals related to protons of alkyl groups of substituted tetrazoles **10**, **11** and protons of the CH-group of the unsubstituted tetrazole ring of the compound **9** is observed. Initially, the splitting of the proton signal of tetrazole ring substituents in the ^1^H NMR spectra was attributed to the formation of a mixture of 4- and 5-isomeric tetrazoles, which was observed for the most spatially hindered phenyl-tetrazole **18** and described in detail in the literature [43,44,45,46]; however, in this case, the ratio of the tetrazole ring proton signals should have shifted towards 1,4-tetrazoles in accordance with the increase in the steric effect of the substituent in the starting compounds. However, this was not found in real spectra. In all cases, the signal was divided into two equal multiplets, in which the spin–spin interaction constants correspond to the proton interaction usual for such groups. The most likely explanation of this phenomenon is the hindrance rotation of the 3-amino-*o*-carborane fragment and tetrazoles relative to the central *s*-triazine ring. To confirm this idea, the NMR spectra of compound **9** were recorded in both acetone-D_6_ (Figure S1) and acetonitrile-D_3_ (Figure S2), since their ability to coordinate with the amino group at the carborane fragment and heterocycles is significantly different. In the less coordinating acetonitrile, an increase in the difference between the signals was found to be almost twice without changing the ratio of their integrals, which indicates in favor of the hypothesis of a hindrance rotation. This conclusion was also supported by the ^1^H NMR spectra of compound **9** recorded in acetone at different temperatures (Figure S4). A gradual broadening and fusion of the proton signals of the tetrazole CH groups with an increase in temperature was observed, which is characteristic of the splitting of the proton signal caused by hindrance rotation (Figure 2).

The structure of compound **9** was also studied with NOESY(EXSY) NMR spectra (Figure S7). The broad signal of the NH proton reveals a lower intensity (0.4) due to the exchange with H_2_O protons. The presence of such an exchange follows from the NOESY(EXSY) NMR spectra (Figure S7). In this spectrum, in addition to antiphase signals related to spatially close CH protons of carborane and tetrazole, in-phase cross peaks of the NH proton (δ = 9.09 ppm) with signals at δ = 3.78 ppm and δ = 2.88 ppm were found. These signals, apparently, correspond to water protons, which has been confirmed by the absence of correlation peaks in the HMQC NMR spectrum (Figure S8). The doubling of the signals in these NMR spectra is due to the different shielding of the indicator signals of compound **9** in the two conformations (see above in the discussion of the results). No such effect was observed in compounds **15**–**17** prepared using mercaptocarboranes.

**Figure 3:**
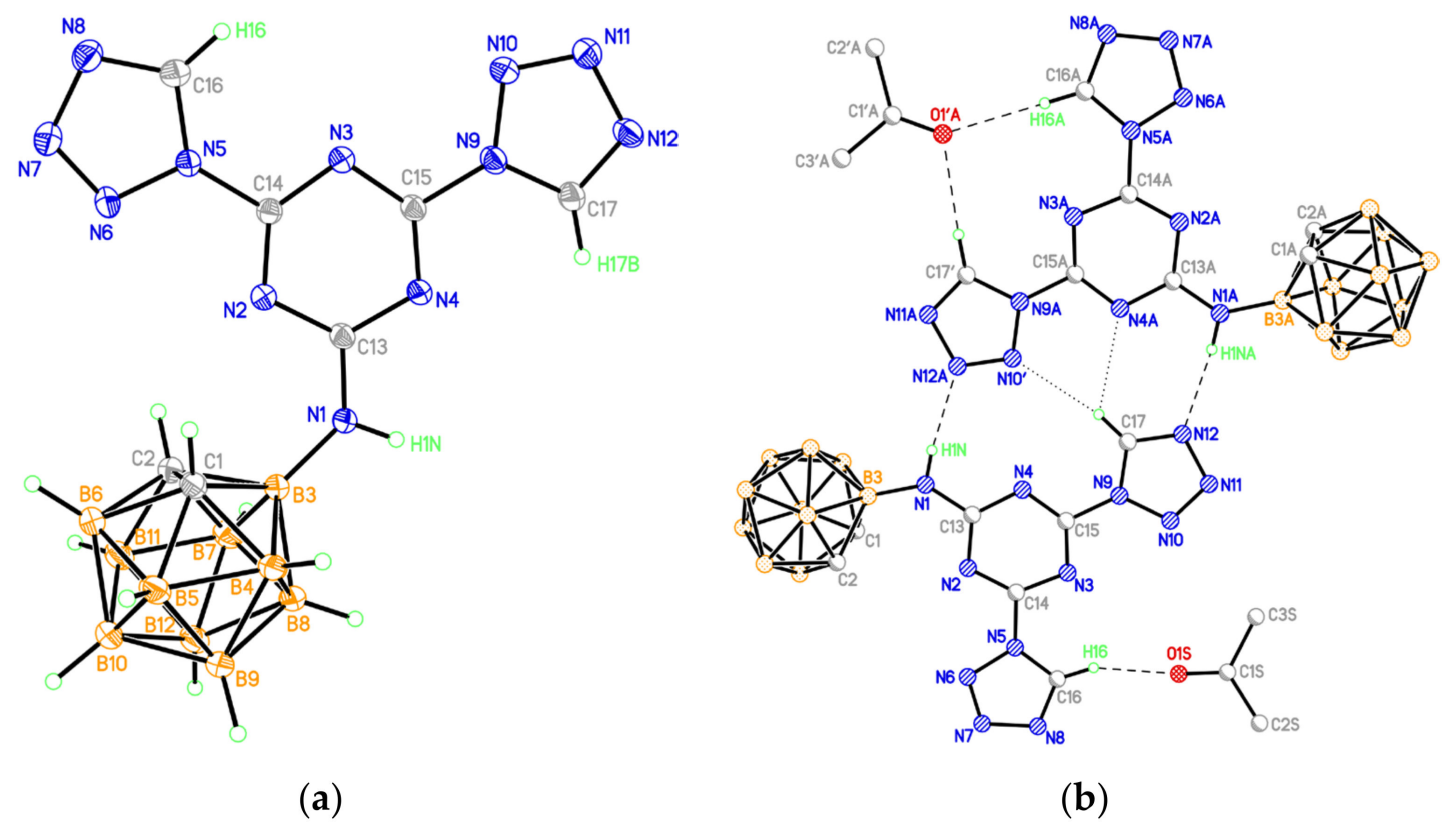
(**a**) Molecular structure of compound **9** (thermal ellipsoids are drawn at the 50% probability level). (**b**) Hydrogen-bonded associate of two conformers of compound **9** (only those hydrogen atoms that are involved in hydrogen bonds are shown).

The structure of compound **9** was also supported by the X-ray diffraction study (Figure 3). This substance crystallizes with one acetone molecule and has a certain degree of disorder in the superposition of the tetrazole CH group and nitrogen atom. Despite the fact that the X-ray data of compound **9** are not completely unambiguous, according to the totality of information based on the NMR spectra and X-ray study and taking into account the data obtained by the X-ray study for **10**, it can be unequivocally stated that the observed splitting of the signal of protons of alkyl groups of substituted tetrazoles **10**, **11** and protons of the CH-group of the unsubstituted tetrazole ring of compound **9** is due to the hindrance rotation of the 3-amino-*o*-carborane fragment and tetrazoles relative to the central *s*-triazine ring.

### 2.3. X-ray Crystallography

**Figure 4:**
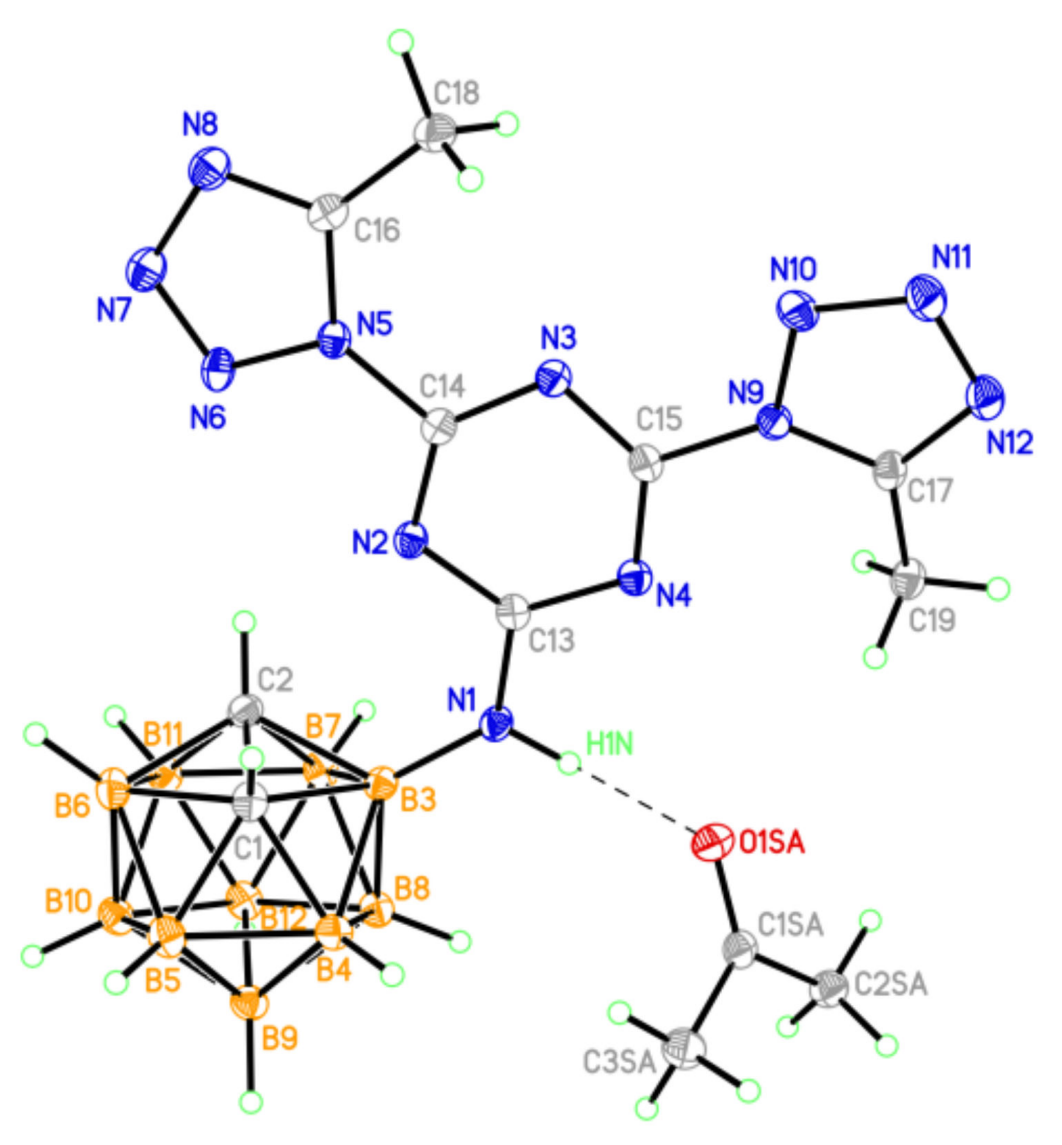
The general view of compound **10** in the crystal (thermal ellipsoids are drawn at the 50% probability level.

**Figure 5:**
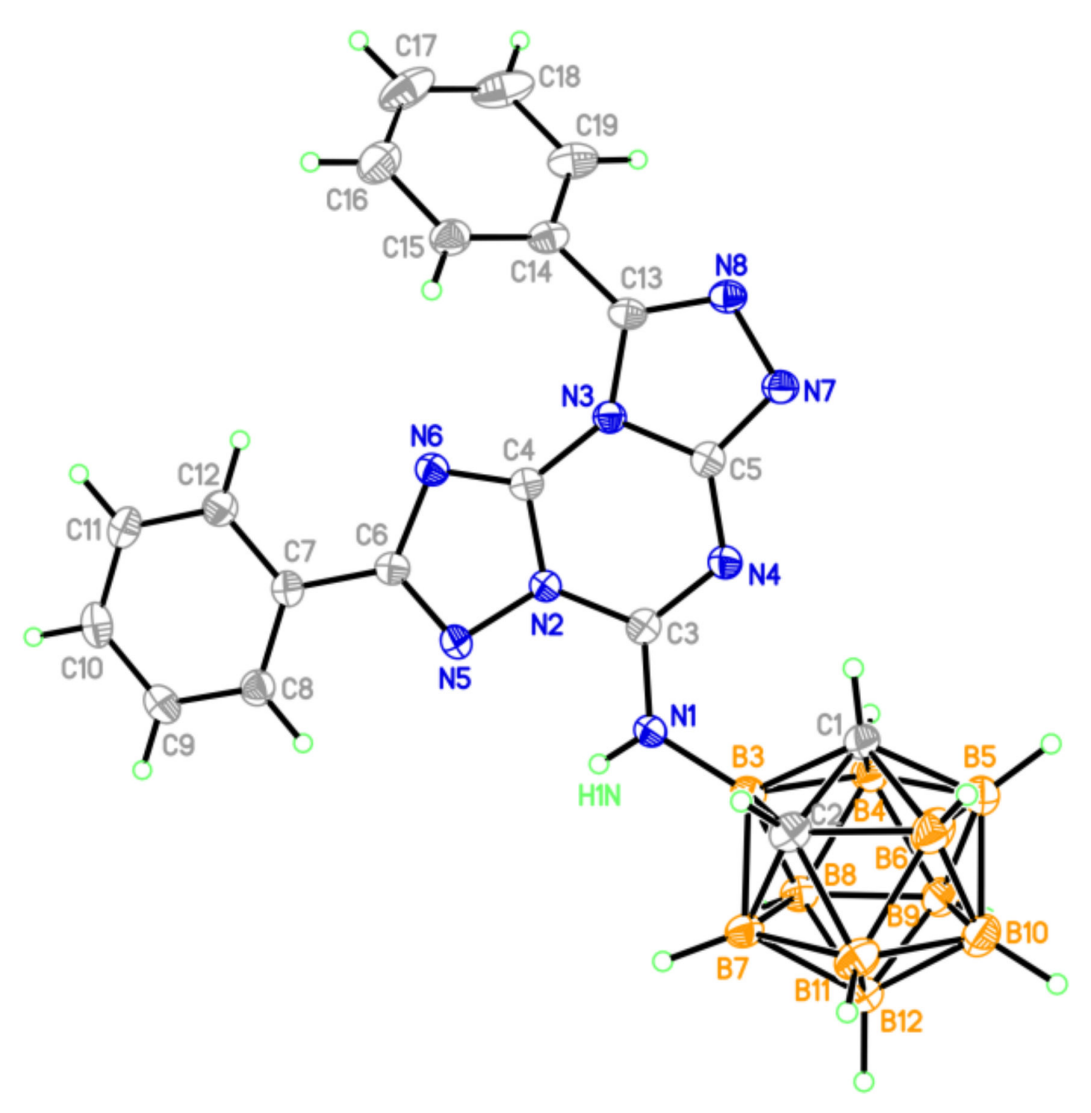
Molecular structure of compound **19** (thermal ellipsoids are drawn at the 50% probability level).

**Figure 6:**
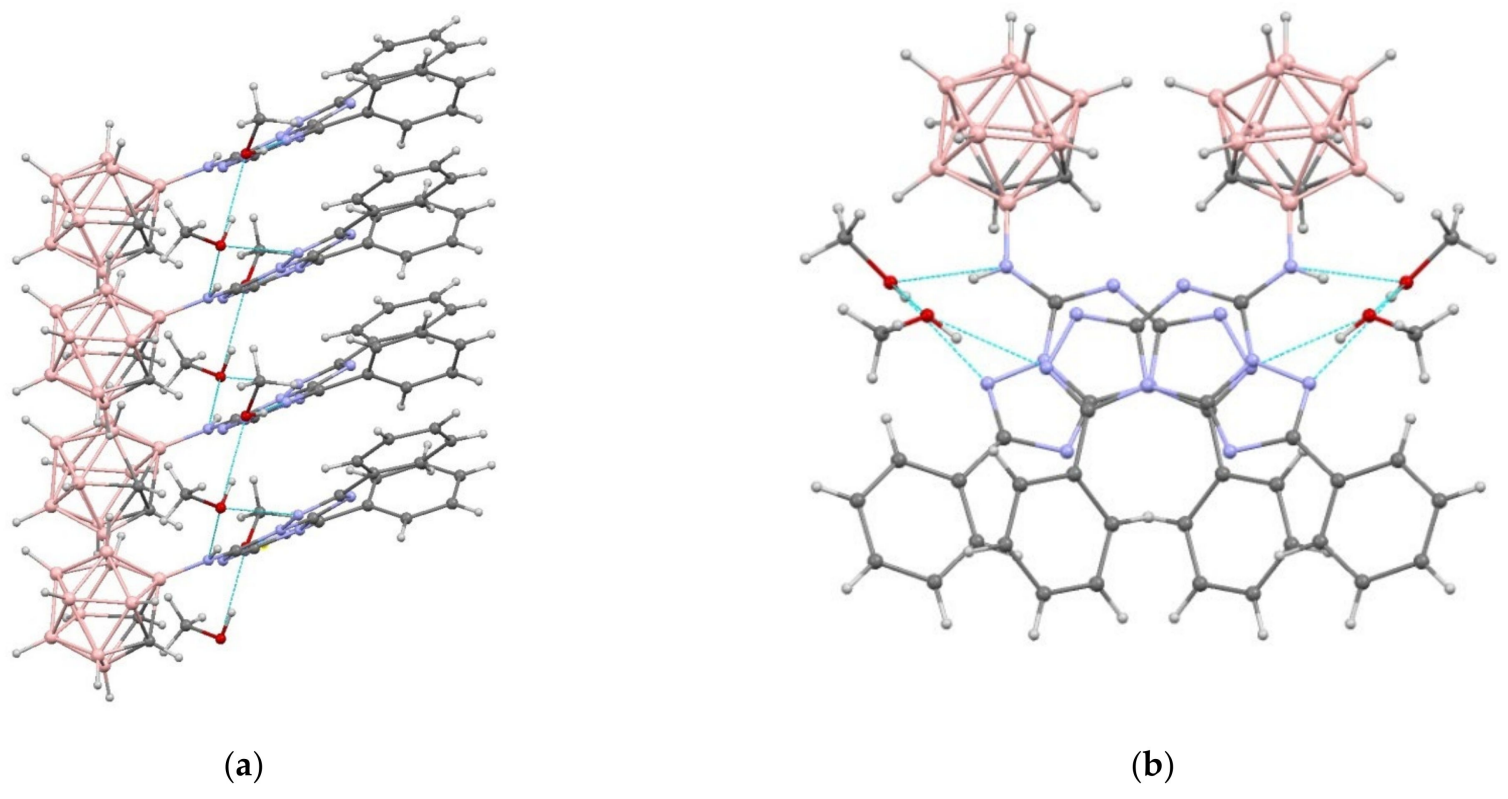
A fragment of an H-bonded stack in the crystal structure of 19 in two projections: (**a**) along the *a* axis of the crystal; and (**b**) along the *c* axis of the crystal.

**Figure 7:**
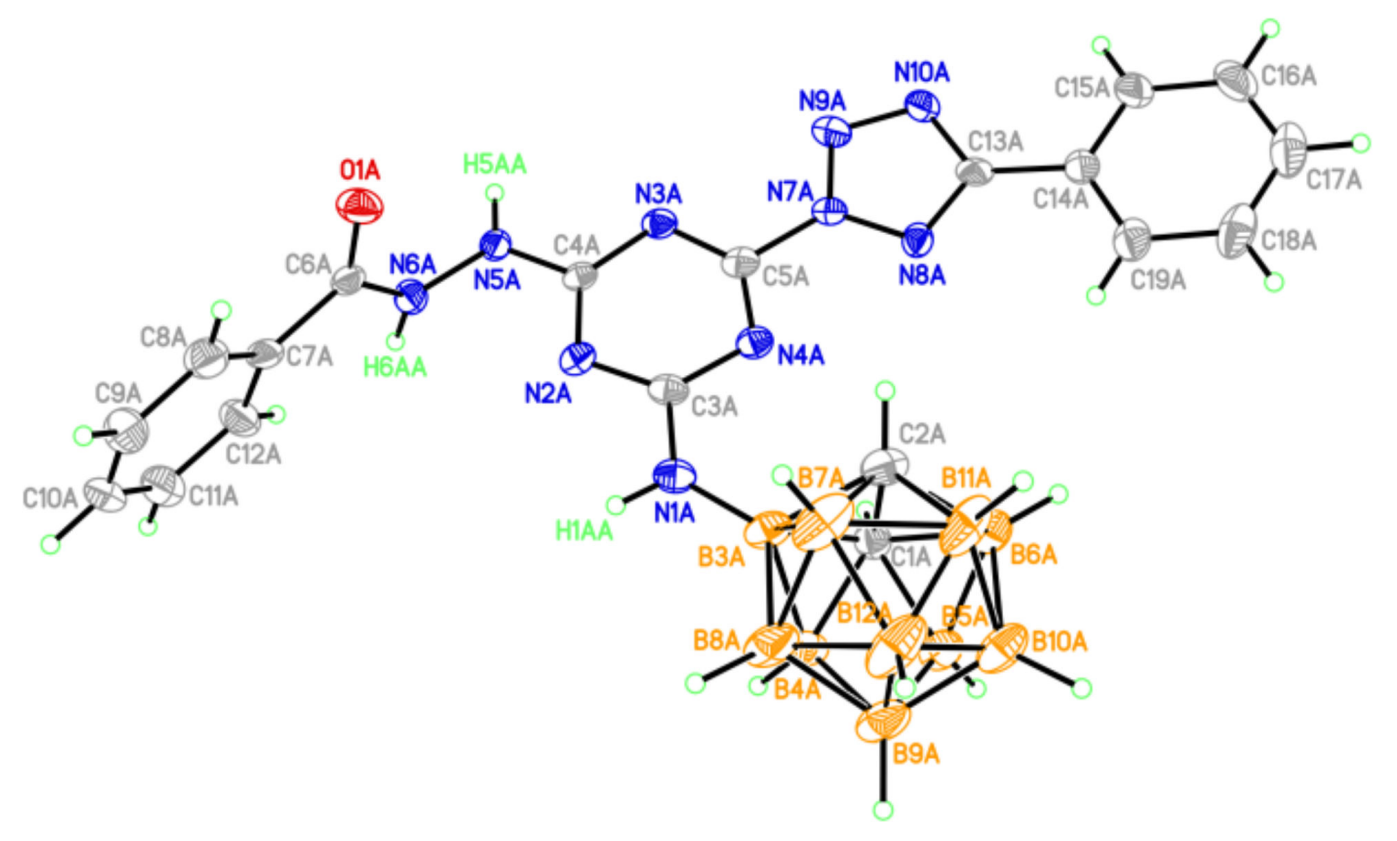
One of two crystallographically independent molecules (A) of compound **20** (thermal ellipsoids are drawn at the 50% probability level).

**Figure 8:**
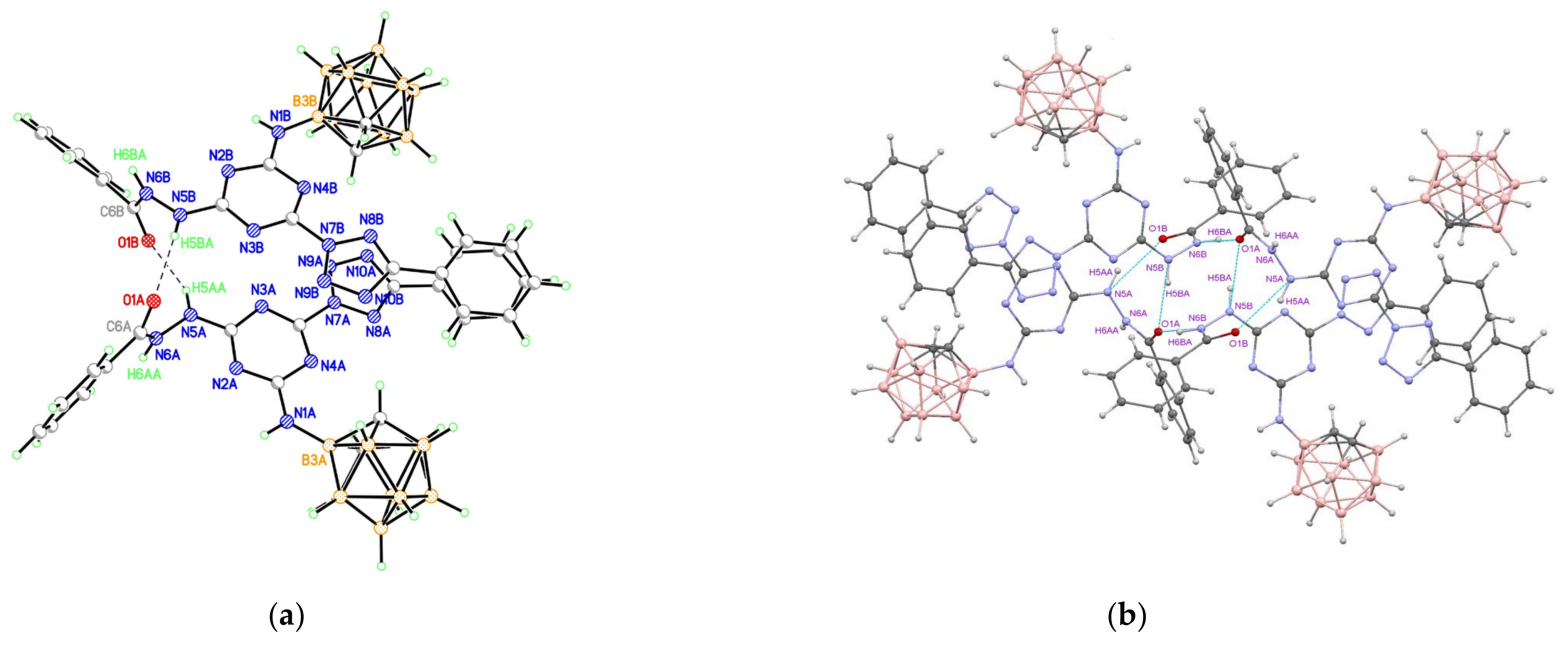
(**a**) H-Bonded dimer formed by two crystallographically independent molecules (A and B) of compound **20**; and (**b**) centrosymmetric H-bonded tetramers.

The crystals of compounds **9** and **10** suitable for X-ray diffraction were obtained by the slow evaporation of acetone at room temperature from the solution of the corresponding compound, whereas crystals of **19** and **20** were obtained by the slow evaporation of the solution system chloroform–methanol (10:1) for **19** and dichloromethane-ethyl acetate (8:1) for **20** at room temperature, respectively. The molecular structures of **9**, **10**, **19** and **20** were determined by means of the single crystal X-ray diffraction study (Figure 3, Figure 4, Figure 5, Figure 6, Figure 7 and Figure 8). Selected data and parameters of X-ray structures for **9**, **10**, **19** and **20** are given in the ESI (Tables S1–S9).

According to the X-ray data, both tetrazole rings in **9** are in the plane of the central *s*-triazine cycle (the dihedral angles between planes of the tetrazole and *s*-triazine units are 3.6 and 7.3°). However, the degree of delocalization in the heterocyclic π-system is low, as evidenced by the noticeable alternation of the lengths of C-N and N-N bonds, especially in five-membered tetrazole rings (Table S1). In the crystal, numerous stacking contacts between the planar heterocyclic systems of adjacent molecules are formed (the interplanar distances are 3.27 and 3.29 Å, the shortest interatomic contacts are C(13)⋯N(7)_−1+x,y,z_ 3.016(2) Å, C(15)⋯N(8)_−1+x,y,z_ 3.186(2) Å, C(15)⋯C(15)_−x,1−y,1−z_ 3.293(2) Å, C(13)⋯N(10)_−x,1−y,1−z_ 3.209(2) Å). The structure of the carborane polyhedron in **9** (bond lengths are C(1)-C(2) 1.650(2), C-B 1.692(2)-1.718(2), B-B 1.767(2)-1.795(2) Å), as in other studied compounds, corresponds to the standard geometry of the icosahedral *o*-C_2_B_10_H_12_ carborane. The positions of N(10) and C(17) atoms (Figure 3a) are occupied by carbon and nitrogen atoms with equal contribution; this observation implies the presence of two conformers of compound **9** in the crystal. One of the possible hydrogen-bonded associates of two conformers of compound **9** united by two N-H⋯N hydrogen bonds and C-H⋯N contacts is presented in Figure 3b.

A solvate acetone molecule is present in the independent part of the unit cell. These solvate molecules are bound to the molecules of compound **9** by either one or two C-H⋯O hydrogen bonds (Table S6). The H-bonded dimers of **9** form stacks along the [1 1 0] crystallographic direction, which, due to π–π interactions between terminal tetrazole rings (the shortest interatomic contact is N(6)⋯N(7)_1−x,2−y,1−z_ 3.042(2) Å), are combined into double layers parallel to *ab* plane (Figure S23). The layers are additionally stabilized due to weak C-H⋯N hydrogen bonds between the carborane fragments and the nitrogen atoms of the tetrazole rings of neighboring molecules (Table S6). Each layer is framed on the outer side by a hydrophobic surface of carborane fragments, and there are no shortened contacts between the layers.

Methyl substituents at the carbon atoms of the five-membered heterocycles in **10** (Figure 4) create steric hindrance to the formation of hydrogen-bonded dimers in the crystal, as observed in structure **9**. In the independent part of the unit cell, there is a solvate acetone molecule, the oxygen atom of which serves as an acceptor of the H-bond with molecule **10** (Table S7).

The methyl substituents lead to a slight violation of the planarity of the heterocyclic system (the dihedral angles between the planes of the tetrazole and *s*-triazine units are 10.3 and 14.6°). Nevertheless, in structure **10**, as in structure **9**, stacking contacts between neighboring molecules are also observed, although with looser stacking (interplanar distances are increased to 3.59 Å). The s-triazine rings in **9** and **10** are flat with an accuracy of 0.02 Å.

In crystal **10**, the supramolecular assembling is close to that observed in **9**. The layered packing with shortened stacking contacts between heterocyclic systems and no specific interactions between layers is observed (Figure S24).

In compound **19** (Figure 5), six and five fused membered rings in the tricyclic fragment have a planar structure (the maximum deviation from the plane of the triazine ring is 0.19 Å for the N8 atom) with phenyl substituents twisted from this plane by 17.3° (at C(6) atom) and 38.6° (at C(13) atom).

Fused heterocycles form compact stacks (Figure 6) extended along the *c* axis of the crystal with interplanar separations of 3.3 Å and shortest interatomic contacts of C(6)⋯N(8)_0.5−x,y, −0.5+z_ 3.122(4) Å and N(6)⋯C(13)_0.5−x,y, −0.5+z_ 3.107(4) Å.

In the independent part of the unit cell of **19**, there are two solvate methanol molecules that additionally stabilize the stack by linking neighboring molecules with a network of H-bonds (Table S8). These stacks are joined in the crystal exclusively due to weak van der Waals interactions, i.e., C-H⋯C, C-H⋯O, and H⋯H. However, in this structure, as in **9** and **10**, layers containing either heterocyclic or carborane fragments of molecules can be distinguished (Figure S25).

In the independent part of the unit cell of **20,** there are two crystallographically independent molecules (A and B) that have the same spatial structure (Figure 7).

In both independent molecules of compound **20**, the tetrazole ring and the phenyl substituent are in the plane of the central *s*-triazine ring (the dihedral angles between planes of these rings range from 2.4 to 11.0°). The benzoylhydrazinyl group is almost orthogonal to the plane of the triazine ring (the C(4)-N(5)-N(6)-C(6) torsion angle is 87.7(4) and 81.8(4)° in A and B, respectively). Similarly to what was observed in structures **9**, **10**, and **19**, in the crystal of **20,** the set of specific intermolecular interactions namely, hydrogen bonds and π–π stacking interactions, were realized. Two independent molecules of compound **20** form centrosymmetric H-bonded tetramers (Figure 8 and Table S9), which are joined in the crystal due to π–π stacking interactions (the interplanar distance is 3.2 Å, the shortest interatomic contacts are C(5A)⋯C(5A)_1−x,1−y,1−z_ 3.223(7) Å, C(3A)⋯N(9A)_1−x,1−y,1−z_ 3.235(7) Å, C(5A)⋯N(9B) 3.222(7) Å). The crystal packing of **20** has a layered structure with alternating heterocyclic and carborane fragments (Figure S26).

#### Hirshfeld Surface Analysis

Hirshfeld surface (HS) analysis is documented as an authoritative tool to qualitatively assess the nature of intermolecular interactions within crystal structures and thoroughly identify the interactions throughout the surface around the molecules [51,52,53,54].

Hirshfeld surface, overall interactions and individual interatomic contacts were computed using the CrystalExplorer17 software [55] for all the compounds studied by X-ray. The obtained data agree well with the X-ray data presented above for molecules **9**, **10**, **19,** and **20** and add a quantification of intermolecular interactions.

Contacts H⋯H contribute the most to the crystal packing of compound **9**, **10**, **19**, **20**, with a percentage contribution from 45.5% to 60.5%. These are the most crucial and significant interatomic contacts. In addition to the H⋯H contacts, the N⋯H and C⋯H contacts are also crucial in defining the crystal packing. Hirshfeld surface analysis showed that important contributions to the crystal packing of compounds **9** (Figures S27 and S28) and **10** (Figures S29 and S30) are from N⋯H interactions (29.2% and 28.5%, respectively), while C⋯H interactions are less essential (less 4%). On the contrary, we found for compounds **19** (Figures S31 and S32) and **20** (Figures S33–S35) that intermolecular C⋯H contacts (14.4% and ~16%, respectively) make the largest contributions to crystal packing, while N⋯H contacts (10.5% and ~12%, respectively) are slightly inferior. Additionally, the notable contribution of N⋯N and C⋯N contacts (7–10%) which corresponds to π–π interactions between flat heterocycle fragments of the molecules should also be noted in all the structures.

In spite of a notable difference in the values of individual interatomic contacts in pairs **9**, **10** and **19**, **20**, the supramolecular packing of all the compounds studied was characterized by common features, namely the alternation of hydrophobic fragments formed by the interaction of boron clusters where H⋯H contacts dominate and fragments with a domination of N⋯H contacts (H-bonds) where heterocycle fragments are grouped. (Figures S36 and S37).

### 2.4. Theoretical Studies of Carborane-Substituted Bis(Tetrazolyl)-s-triazines (***9***–***11***, ***15***–***17***)

#### 2.4.1. Computational Details

The calculations of compounds **9**–**11**, **15**–**17** were performed according to density functional theory (DFT) [56]. The Becke–Lee–Yang–Parr hybrid functional (B3LYP) [57,58] was applied in basis 6-311 + G*. All calculations with a full optimization of the geometry of molecules and calculation of normal oscillation frequencies at 298 K and 1 atm were performed using the GAUSSIAN-09 program [59] under the LINUX operating system. If the imaginary frequencies of normal vibrations appeared, the optimization was repeated. The accuracy of the results of the calculated geometry optimization was supported by the comparison of the computed data with the experimental ones obtained for compounds **9** and **10** by X-ray single crystal analysis.

#### 2.4.2. Geometry and Electronic Structure

**Figure 9:**
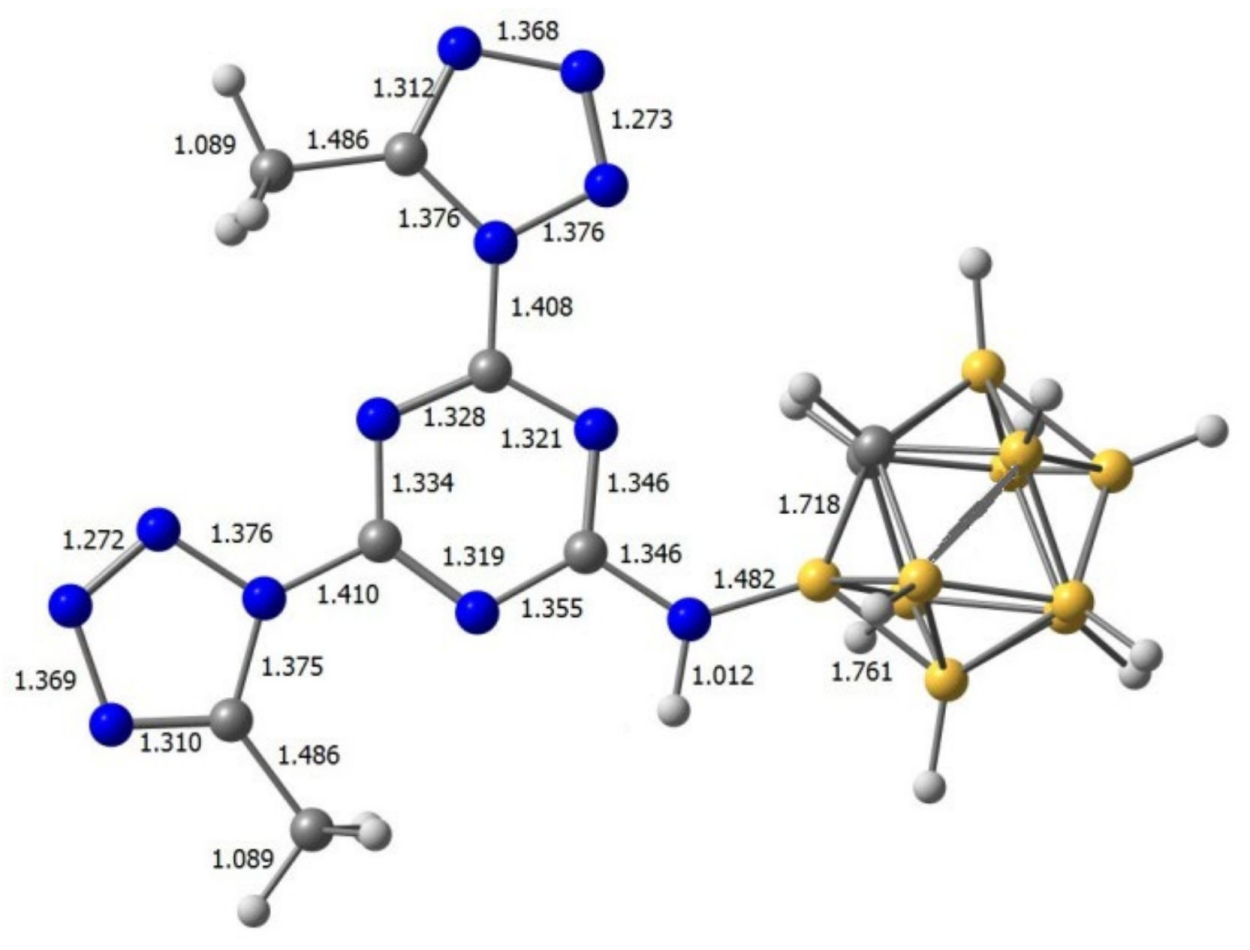
Calculated geometry of compound **10**.

The results of the geometry optimization for all prepared compounds are given in Table S10 (Supplementary Materials), while the geometry of molecule **10** is presented below (Figure 9).

A comparison of the experimental and theoretical interatomic distances of molecule **10** (Table 1) demonstrates that the calculations at the DFT B3LYP/6-311 + G* level can be considered as an adequate one for the geometry optimization of the carborane-substituted bis(tetrazolyl)-*s*-triazines.

The calculated values of the total energy and entropy of compounds **9**–**11**, **15**–**17** (6-311 + G* basis set) in the gas phase based on computed geometries are listed in Table S11.

The thermal stability and impact sensitivity of HEDM are of great importance during their manufacturing, storage and handling. The energy gaps (Δε) between HOMO and LUMO help characterize the chemical reactivity and kinetic stability of molecules [60,61,62,63]. It is quite expected that the variation of the nature of substituents, their position and number can change the value of the HOMO–LUMO gap which thus affects the reactivity and stability of the compounds.

In recent works [64,65], the B3LYP functional was successfully used for carborane systems and the accuracy of the applied DFT/B3LYP method was confirmed by calculations in the MRCC3 software package [64].

**Figure 10:**
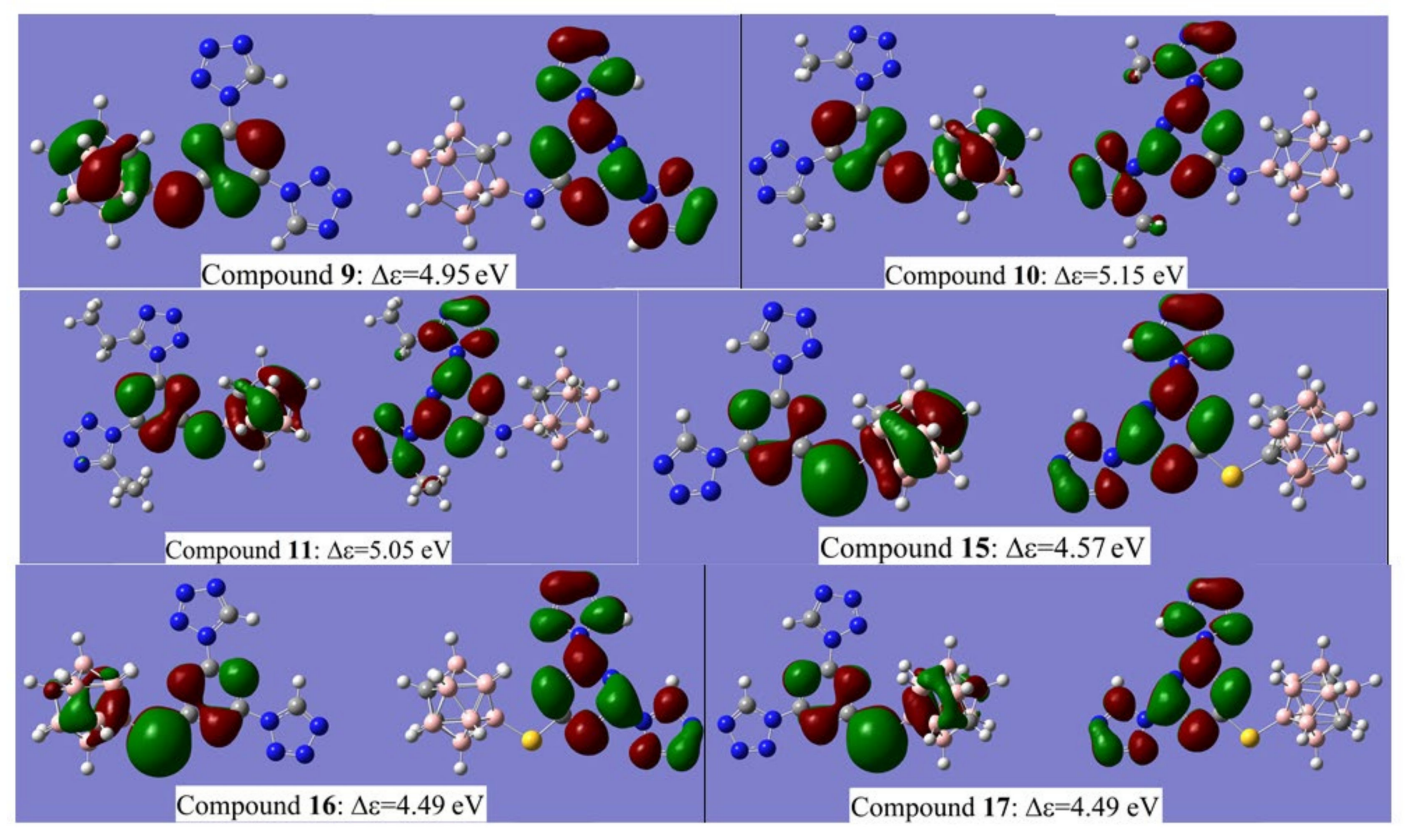
Plots of HOMO (left) and LUMO (right) *iso*-surfaces for compounds **9**–**11** and **15**–**17**.

The calculated values of HOMO and LUMO energies based on the DFT B3LYP/6-311 + G* level for the compounds **9–11** and **15–17** in a gas phase are presented in Table 2 while the plots of HOMO and LUMO iso-surfaces are provided in Figure 10.

The results of the visualization of HOMO and LUMO *iso*-surfaces demonstrate that for all molecules studied, the HOMO *iso*-surface is mainly localized on carborane and *s*-triazine moieties and either on the -NH- or -S- linker. The LUMO *iso*-surface can be found on tetrazole and *s*-triazine fragments. The substitution of the boron atom of carborane cages elevates both HOMO and LUMO levels, while on carbon atom reduces them. Methyl and ethyl substituents in tetrazole rings attached to *s*-triazine elevate the HOMO energy and reduce LUMO energy. The presence of sulfur linker elevates both HOMO and LUMO energy levels. Due to this complicated influence of the structural units, compounds **9**, **10** and **11** are characterized by the largest computed values of Δε. These are 116, 121 and 119 kcal/mol, respectively. However, magnitudes of Δε found for compounds **15**, **16** and **17** are less (107, 105 and 105 kcal/mol) compared to **9**, **10** and **11**, as these types of energetic materials are still less impact-sensitive than classical diaminotrinitrobenzene (DATB) and trinitroaniline (TNA), whose Δε values are 91 kcal/mol and 90 kcal/mol, respectively [60].

#### 2.4.3. The Evaluation of the Enthalpy of Formation (EOF) of the Carborane-Substituted Bis(Tetrazolyl)-s-triazines in a Gas Phase

EOF is an important parameter for the description of the energetic materials. As there are few experimental data on the EOFs of the high-energy carborane compounds studied in the literature, the application of quantum-chemical calculations becomes essential. The EOF estimation of the compounds requires the knowledge of their total energies and the total energies of the combining elements at the standard conditions.

For the estimation of the EOFs of HEMDs, the method of isodesmic reactions is used [66] and good results can be achieved in the reactions where atomic groups are kept invariable. However, only the precise experimental values for all the model compounds allow you to attain the high accuracy. To overcome the errors caused by the difference in the experimental magnitudes of the EOF of the model compound, it is needed to consider at least several isodesmic reactions with the various components. However, for some compounds under investigation, no experimental data on EOFs even for the possible model compounds were found.

**Figure 11:**
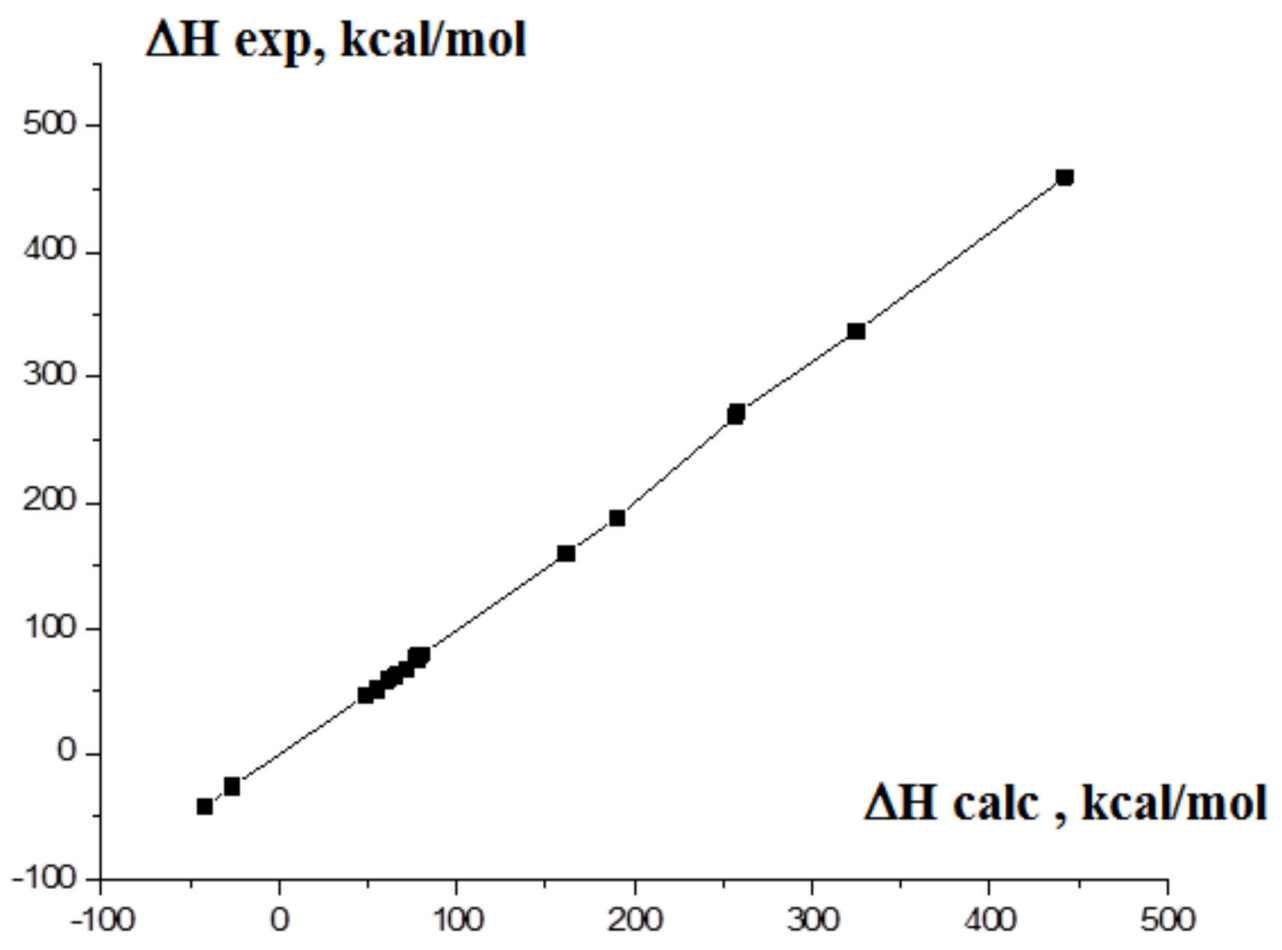
The interdependence of the calculated and experimental EOF values of tested compounds.

As a result, the EOF values based on DFT B3LYP/6-311 + G* calculations for the molecules in a gas phase were used in the present work. As in the case of graphite and crystalline boron, such computations are inapplicable, and the assumption about the equality of the calculated and experimental EOF values for the gas molecules of 2H-tetrazole and o-carborane was applied [38,67,68]. The total energies with the correction on zero vibrations E_zpc_ were used. These computed parameters are E′(C) = −38.12527664 a.u., E′(B) = −24.867864 a.u., E′(S) = −398.2066 a.e. To confirm the reliability of the data found for carbon, boron and sulfur, the calculations for similar molecules were carried out. The theoretical results compared with the experimental ones are presented in Table S7. The data presented in Table S7 demonstrate that this method of the estimation of EOF gives a deviation of the calculated values from the experimental ones on 4–5%. The plot of the interdependence of our calculated EOF magnitudes and the experimental ones for the test compounds is shown on Figure 11.

Using the values of parameters [32] for the crystalline carbon and boron, the EOF magnitudes of compounds **9**–**11** and **15**–**17** were calculated (see Table 3).

According to the data of Table 3, the lowest EOF values can be found in the case of molecules with an NH-linker which indicates their greater stability among the studied compounds. As for energetic properties, all of them are characterized by rather high EOF magnitudes and can be considered to be promising HEDMs as their EOF values are notably higher than those of trinitrotoluene (TNT) and hexogen (RDX, Research Department Explosive) (79.45 and 59.20 kcal/mol), respectively, calculated in [69].

#### 2.4.4. Predicted Detonation Performances

The detonation velocity and the pressure of detonation are important parameters in the field of explosives engineering. For the computation of the detonation properties of carborane-substituted bis(tetrazolyl)-*s*-triazines, the method given in [70,71] was applied. The detonation velocity (D) and the pressure of detonation (P) are described with Kamlet–Jacobs semi-empirical equations:

D = 1.01·(N·M^1/2^·Q^1/2^)^1/2^·(1 + 1.30 p_0_); P = 1.558·p_0_^2^·N·M^1/2^·Q^1/2^

where D is the velocity of detonation (km/s); P—pressure of detonation (kbar); N—a mole number of detonated gas per mole of the explosive material (EM); M—mole mass (g/mol) of the detonated gas; Q—heat of explosion EM (J/g); and p_0_—density of EM (g/cm^3^).

The computed values of the heat of explosion Q (J/g), the velocity of detonation D (km/s), and the pressure of detonation P (GPa) of molecules **9**–**11** and **15**–**17** in a gaseous phase are given in Table 4.

Based on the dataset of Table 3 and Table 4, all the studied carborane-substituted bis(tetrazolyl)-*s*-triazines can be considered to be promising candidates for HEDMs as the EOFs of all these compounds exceeds the EOF of the known explosive RDX (189.5 kJ/mol or 45.3 kcal/mol) [72,73]. The EOF values for the most energetic compounds **9**, **15** and **16** are 4.0, 4.7 and 3.9 times larger than the same parameters in the case of RDX. Moreover, the computed pressure of the detonation of the compounds **9**, **10**, **11** and **15** exceeds the pressure at the front of the RDX shock wave (approximately 30 GPa [74,75,76]) by 1.7-fold, 2.5-fold, 2.0-fold and 1.8-fold, respectively.

#### 2.4.5. Calculation of Terahertz Spectra of Compounds **9**–**11** and **15**–**17**

The development of technologies to perform the remote detection of dangerous substances is in very high demand in different fields. Recently, these methods have been complemented by impulse terahertz spectroscopy. Many compounds are characterized by the spectral fingerprints in the terahertz range (usually in the ~0.1–3.0 THz region [77]) which makes terahertz radiation suitable for their identification. Moreover, the transparency of many materials to THz radiation discovers the great possibility for the application of this technique in the security maintenance.

In this section of the paper, the computed frequencies (*v*; THz) and the corresponding intensities (H; A^4^/AMU, AMU—reduced masses) of the terahertz spectra (DFT B3LYP/6-311 + G*) of compounds **9**–**11** and **15**–**17** in the 0.1–3.0 THz range are presented in Table 5.

## 3. Experimental

### 3.1. Materials and Methods

^1^H and ^11^B NMR spectra were recorded on a Bruker Avance-400 spectrometer operating at 400.13 MHz for ^1^H NMR and 128.28 MHz for ^11^B NMR and Bruker AvanceTM 500 spectrometer (Bruker BioSpin AG, Zurich, Switzerland) (at 500.13, 125.47 MHz for ^1^H and ^13^C, respectively). Chemical shifts (δ) were referenced to the residual solvent peak acetone-D_6_, 2.05 ppm, acetonitrile-D_3_ 1.93 ppm, for ^1^H, external BF_3_ OEt_2_ for ^11^B. IR spectra were recorded on a Bruker FTIR spectrometer Tensor 37 in KBr pellets. Merck silica gel L0.040–0.080 mesh was used for column chromatography. The identities of new compounds were verified by TLC on Silufol plates.

Bruker AvanceTM 500 spectrometer (Germany) (at 500.13, 160.46, and 125.47 MHz for ^1^H, ^11^B and ^13^C, respectively). The ^1^H chemical shifts were measured relative to the signal of the solvents, acetonitrile-D_3_ and acetone-D_6_.

### 3.2. X-ray Crystallography

Single-crystal X-ray diffraction experiments were carried out with a Bruker SMART APEX II diffractometer (graphite monochromated Mo-K_a_ radiation, λ = 0.71073 Å, ω-scan technique). All structures were solved by the direct methods and refined by the full-matrix least squares technique against *F^2^* with anisotropic thermal parameters for all non-hydrogen atoms using the SHELXL software, version 2018/3, Bruker AXS Inc., Madison, WI, USA [78]. Figure 3a,b, Figure 4, Figure 5, Figure 7 and Figure 8a were made with an XP program included in SHELXL program package [78]. Figure 6a,b and Figure 8b were made with Mercury (version 4.3.1) [79]. Hirshfeld surfaces and the associated 2D fingerprint plots were calculated using the CrystalExplorer17 software [55]. In structures **9**, **10** and **19**, the hydrogen atoms of the carborane ligands, as well NH and OH groups were located from the Fourier syntheses and isotropically refined without restrictions. The remaining hydrogen atoms in these structures and all hydrogen atoms in structure **20** were placed geometrically and included in the structure factors calculation in the riding motion approximation. In structure **20**, the highly disordered solvate molecules (presumably, methylene chloride) were detected in the residual density. Unfortunately, these solvate molecules could not be successfully modeled even with restraints. The contribution of undefined electron density peaks was treated as diffusion scattering, and it was excluded using the SQUEEZE routine implemented in the PLATON program [80]. Crystal data and the parameters of the refinements for **9**, **10**, **19** and **20** are listed in Table S5. The crystallographic data for the structures for compounds **9**, **10**, **19** and **20** have been deposited at the Cambridge Crystallographic Data Centre (CCDC) as supplementary publications No. CCDC 2177931, 2177932, 2177933 and 2177934, respectively.

### 3.3. Method of Calculation

The evaluation of geometry optimization, enthalpies of formation (EOF), energetic properties, stability, the detonation properties and terahertz spectra of selected compounds were estimated based on DFT B3LYP/6-311 + G* [56,57,58] calculations using GAUSSIAN-09 [59] under LINUX (see Section 2.4.1).

### 3.4. General Procedure for the Preparation of 4,6-bis(5-Alkyltetrazol-1-yl)-2-[(o-Carborane-3-yl)Amino]-1,3,5-Triazines (***9***–***11***)

To a solution of 2,4,6-trichloro-1,3,5-triazine **1** (0.5 g, 2.7 mmol) in dry THF (8 mL), a mixture of DIPEA (1 mL, 0.74 g, 5.7 mmol) and the corresponding tetrazole **2**, **3** or **4** (5.43 mmol) in dry THF (5 mL) was added dropwise at −30 °C–−40 °C within 20 min under an argon atmosphere. Then, the resulting mixture was slowly (2–4 h) warmed to room temperature and stirred until the starting compounds disappeared (TLC control, CHCl_3_-Acetone 10:1) to form ditetrazolyl-1,3,5-triazines **5**–**7**. After that, a mixture of DIPEA (0.38 g, 2.86 mmol) and 3-amino-*o*-carborane **8** (0.44 g, 2.8 mmol) in THF (5 mL) was slowly added at −10 °C to the corresponding reaction mixture of compounds **5**, **6** or **7** and then stirred at ambient temperature until the carborane **8** disappeared (4 h for **9**, 8h for **10**, and 10 h for **11**, TLC control, toluene). Upon reaction completion, the solvents were evaporated in vacuo, the residue was dissolved in EtOAc (100 mL) and washed with water (3 × 100 mL) and the organic layer was dried over Na_2_SO_4_. EtOAc was evaporated to dryness and the residue of the prepared compound **9**, **10** or **11** was treated as indicated below.

#### 3.4.1. 2-[(*o*-Carborane-3-yl)amino]-4,6-di(1H-Tetrazol-1-yl)-1,3,5-triazine (**9**)

The residue was treated with Et_2_O (20 mL), the precipitate that formed was filtered off and washed with Et_2_O–CHCl_3_ (1: 2, 50 mL) mixture, and dried to yield compound **9** 0.85 g (84%); ^1^H NMR (acetone-D_6_, 400.13 MHz) *δ*: 10.15 (s, the rotamer 1, 1H, N_4_CH), 10.11 (s, the rotamer 2, 1H, N_4_CH), 10.13 (s, *J* = 14 Hz, 2H, N_4_CH),] 9.08 (br s, 1H, carborane NH), 5.33 (br s, 2H, carborane CH); ^13^C NMR (acetone-D_6_, 125.77 MHz) *δ*: 169.73 (s, 1C, **C**-2 1,3,5-triazine), 160.64 and 160.79 (s, 2C, **C** 4,6-position of 1,3,5-triazine *), 143.11 and 143.19 (s, 2C, **C**H-tetrazole *); 57.26 (s, 2C, **C**H-carborane, 29.72 (s, (**C**H_3_)_2_CO **); ^11^B NMR (acetone-D_6_, 128.28 MHz) *δ*: −4.4 (d, *J* = 144 Hz, 2B), −6.2 (s, 1B, B^3^), −10.1 (d, *J* = 149 Hz, 1B), −12.8 (d, *J* = 170 Hz, 2B), −14.3 (d, *J* = 172 Hz, 4B);. IR (KBr) ν: 3067 (carborane CH), 2598 (BH), 1614, 1585, 1472, 1451(N=N) cm^−1^.

* The doubling of the signals in these NMR spectra is due to the different shielding of the indicator signals of compound **9** in the two conformations

** Complex of acetone with compound **9**.

#### 3.4.2. 2-[(*o*-Carborane-3-yl)amino]-4,6-di(5-Methyl-1H-tetrazol-1-yl)-1,3,5-triazine (**10**)

The residue was treated with Et_2_O (20 mL), the precipitate that formed was filtered off and washed with Et_2_O–CHCl_3_ (2:1, 50 mL) mixture, and dried to yield compound **10** 0.77 g (70%); ^1^H NMR (acetone-D_6_, 400.13 MHz) *δ*: 8.99 (br s, 1H, NH), 5.32 (br s, 2H, carborane CH), 3.01 (s, the rotamer 1, 3H, CH_3_), 2.96 (s, the rotamer 2, 3H, CH_3_); ^11^B NMR (acetone-D_6_, 128.28 MHz), *δ*: −4.5 (d, *J* = 147 Hz, 2B), –6.1 (s, 1B, B^3^), –10.2 (d, *J* = 154 Hz, 1B), −12.8 (d, *J* = 177 Hz, 2B), −14.4 (d, *J* = 182 Hz, 4B). IR (KBr) ν: 3038 (carborane CH), 2587 (BH), 1609, 1574, 1549 (N=N) cm^−1^.

#### 3.4.3. 2-[(*o*-Carborane-3-yl)amino]-4,6-di(5-Ethyl-1H-tetrazol-1-yl)-1,3,5-triazine (**11**)

The residue was treated with hexane (20 mL), the precipitate that formed was filtered off and washed with Et_2_O (60 mL) and dried to yield compound **11** 0.70 g (59%); ^1^H NMR (acetone-D_6_, 400.13 MHz) *δ*: 9.01 (br s, 1H, NH), 5.33 (br s, 2H, carborane CH), 3.48 (q, the rotamer 1, *J* = 7.3 Hz, 2H, CH_2_), 3.43 (q, the rotamer 2, *J* = 7.3 Hz, 2H, CH_2_), 1.45 (t, the rotamer 1, *J* = 7.3 Hz, 3H, CH_3_), 1.42 (t, the rotamer 2, *J* = 7.3 Hz, 3H, CH_3_); ^11^B NMR (acetone-D_6_, 128.28 MHz) δ: −4.5 (d, *J* = 147 Hz, 2B), −6.0 (s, 1B, B^3^), −10.2 (d, *J* = 147 Hz, 1B), −12.8 (d, *J* = 170 Hz, 2B), −14.3 (d, *J* = 172 Hz, 4B). IR (KBr) ν: 3032 (carborane CH), 2588 (BH), 1607, 1577, 1551 (N=N) cm^−1^.

### 3.5. General Procedure for the Preparation of 2-[(Carborane-9-yl)tio]-4,6-di(1H-Tetrazol-1-yl)-1,3,5-triazines (***15***, ***16***, ***17***)

To a solution of ditetrazolyl-1,3,5-triazine **5** prepared from triazine **1** (0.5 g, 2.7 mmol) and tetrazole **2** (0.38 g, 5.43 mmol) in dry THF (8 mL) and DIPEA (1 mL, 0.74 g, 5.7 mmol) with stirring for 3 h, a mixture of DIPEA (0.38 g, 2.86 mmol) and 1-mercapto-o-carborane **13** (0.49 g, 2.8 mmol) in THF (5 mL) was slowly added at –10 °C. The reaction mixture was then stirred at ambient temperature for 3 h. THF was evaporated in vacuo, the residue was treated with hexane (20 mL) and the formed precipitate was filtered off and washed with Et_2_O (20 mL).

#### 3.5.1. 2-[(Nido-7,8-dicarbaundecaboran-7-yl)tio]-4,6-di(1H-tetrazol-1-yl)-1,3,5-triazinyl] Esium (**15**)

After the general procedure, the solid was washed with CHCl_3_ (20 mL). After that, the solid was dissolved in H_2_O–acetone mixture (10:1) and CsCl (0.67 g, 4 mmol) was added to the obtained solution. The acetone was removed in vacuo, the formed precipitate was filtered off, washed with water and crystallized (CHCl_3_– acetone 20:1) to yield compound **15** 0.89 g (50%); ^1^H NMR (acetone-D_6_, 400.13 MHz) *δ*: 8.95 (s, 2H, N_4_CH), 3.69 (br s, 1H, carborane CH), 3.16 (br s, 1H, carborane CH); ^11^B NMR (acetone-D_6_, 128.28 MHz) *δ*: −3.0 (d, *J* = 145 Hz, 1B), −9.2 (d, *J* = 144 Hz, 3B), −12.4 (d, *J* = 175 Hz, 2B), −18.8 (d, *J* = 149 Hz, 1B) −22.8 (d, *J* = 123 Hz, 1B), –34.4 (dd, *J* = 50.0, 123 Hz, 1B); IR (KBr) ν: 3070 (carborane CH), 2578 (BH), 1579, 1467(N=N) cm^−1^.

#### 3.5.2. 2-[(*o*-Carborane-9-yl)tio]-4,6-di(1H-tetrazol-1-yl)-1,3,5-triazine (**16**)

Yield: 0.77 g (70%); ^1^H NMR (acetone-D_6_, 400.13 MHz) *δ*: 10.15 (s, 2H, N_4_CH), 4.90 (br s, 1H, carborane CH), 4.84 (br s, 1H, carborane CH); ^11^B NMR (acetone-D_6_, 128.28 MHz) *δ*: 1.8 (s, 1B, B^9^), −2.6 (d, *J* = 149 Hz, 1B), −8.9 (d, *J* = 153 Hz, 2B), −13.3 (d, *J* = 156 Hz, 2B), −14.4 (d, *J* = 154 Hz, 4B); IR (KBr) ν: 3063 (carborane CH), 2613 (BH), 1580, 1465 (N=N) cm^−1^.

#### 3.5.3. 2-[(*m*-Carborane-9-yl)tio]-4,6-di(1H-tetrazol-1-yl)-1,3,5-triazine (**17**)

Yield: 0.88 g (79%). ^1^H NMR (acetone-D_6_, 400.13 MHz) *δ*: 10.17 (s, 2H, N_4_CH), 3.98 (br s, 2H, carborane CH); ^11^B NMR (acetone-D_6_, 128.28 MHz) *δ*: −4.4 (s, 1B, B^9^), −5.8 (d, *J* = 170 Hz, 2B), −10.2 (d, *J* = 151 Hz, 1B), −12.5 (d, *J* = 139 Hz, 2B), −13.5 (d, *J* = 153 Hz, 2B), −16.6 (d, *J* = 177 Hz, 1B), –17.5 (d, *J* = 150 Hz, 1B). IR (KBr) ν: 3053 (carborane CH), 2624 (BH), 1580, 1466 (N=N) cm^−1^.

### 3.6. General Procedure for the Preparation of Compounds ***19***–***21***

To a solution of 2,4,6-trichloro-1,3,5-triazine **1** (0.5 g, 2.7 mmol) in dry THF (8 mL), a mixture of DIPEA (1 mL, 0.74 g, 5.7 mmol) and 5-phenyltetrazole (0.78 g, 5.43 mmol) in dry THF (5 mL) was added dropwise at −50 °C within 20 min under an argon atmosphere. Then, the resulting mixture was slowly (18 h) warmed to room temperature and stirred until the starting compounds disappeared (TLC control, CHCl_3_–acetone 10:1). After that, a mixture of DIPEA (0.38 g, 2.86 mmol) and 3-amino-*o*-carborane **8** (0.44 g, 2.8 mmol) in THF (5 mL) was slowly added at −20 °C to the reaction and then stirred at ambient temperature until carborane **8** disappeared (15 h, TLC control, toluene). Upon the reaction completion, the solvents were evaporated in vacuo, the residue was dissolved in EtOAc (100 mL), washed with water (3 × 100 mL), the organic layer was dried over Na_2_SO_4_, evaporated to dryness and the residue was treated with hexane (15 mL) and Et_2_O (15 mL), the formed solid was purified by column chromatography on SiO_2_ using hexane-EtOAc 5:1 and 1:2 systems as an eluent.

#### 3.6.1. 5-(*o*-Carborane-3-yl)amino-2,9-diphenyl-bis[1,2,4]triazolo[1,5-a:1′,5′-c][1,3,5]triazine (**19**)

Yield: 0.23 g (18%); ^1^H NMR (acetone-D_6_, 400.13 MHz) *δ*: 9.09 (br s, 1H, NH), 8.25 (m, 4H, Ph), 7.61 (m, 6H, Ph), 5.48 (br s, 2H, carborane CH); ^11^B NMR (acetone-D_6_, 128.28 MHz) *δ*: −4.5 (d, *J* = 141 Hz, 2B), −6.0 (s, 1B, B^3^), −10.2 (d, *J* = 161 Hz, 1B), −12.7 (d, *J* = 161 Hz, 2B), −14.5 (d, *J* = 158 Hz, 4B); IR (KBr) ν: 3072 (carborane CH), 2560 (BH) cm^−1^.

#### 3.6.2. 2-(2-Benzoylhydrazinyl)-4-(*o*-carborane-3-yl)amino-6-(5-phenyl-2H-tetrazol-2-yl)-1,3,5-triazine (**20**)

Yield: 0.14 g (10%); ^1^H NMR (acetone-D_6_, 400.13 MHz) *δ*: 9.39 (m, 1H, NH), 8.28 (m, 2H, Ph), 8.14 (m, 2H, Ph), 8.14 (m, 1H, Ph), 8.02 (m, 1H, Ph), 7.60 (m, 6H, Ph), 5.39 (m, 2H, carborane CH); ^11^B NMR (Acetone-D_6_, 128.28 MHz), *δ*: −4.9 (d, *J* = 141 Hz, 3B), −10.3 (d, *J* = 141 Hz, 1B), −12.9 (d, *J* = 163 Hz, 2B), −14.9 (d, *J* = 170 Hz, 4B); IR (KBr) ν: 3072 (carborane CH), 2565 (BH) cm^−1^.

#### 3.6.3. 3,7,11-Triphenyl[tris([1,2,4]triazolo)[4,3-a:4′,3′-c:4″,3″-e][1,3,5]triazine (**21**) [47,49,50]

Yield: 70 mg (6%); ^1^H NMR (acetone-D_6_, 400.13 MHz) δ: 8.05 (dd, *J* =1.9, 8.1 Hz, 2H,), 7.65 (m, 3H).

## 4. Conclusions

Recently, more attention has been focused on the design and synthesis of modern high-energy compounds based on nitrogen heterocycles and other energetic entities having energy characteristics suitable for the potential replacement of the traditional energetic materials.

In this article, a series of new high-energy compounds was successfully synthesized and fully characterized based on the one-stage functionalization of cyanuric chloride with nitrogen-rich 5-R-tetrazoles (R = H, Me, Et) and aminocarborane or mercaptocarboranes, reducing the number of reaction steps and therefore the amount of by-products in the preparation of these compounds. All the components of prepared compounds have been widely used while designing energetic materials. Synthesized compounds have high enthalpies of formation and excellent energetic properties such as heat of combustion and the heat of the explosion which makes these compounds promising alternatives for commonly used energetic compounds. Based on the DFT calculations, it was shown that compound **15** possessed the maximum value of the detonation velocity of all the considered compounds, and compound **10** demonstrated the maximum value of the detonation pressure. Theoretical terahertz frequencies have been calculated for potential high-energy density materials (HEDMs), enabling the remote detection of these compounds.

## Figures and Tables

**Table 1:** Comparison of the experimental and theoretical interatomic distances for molecule **10**.

Interatomic Distances	Experiment, Å	Calculations, Å
N=N (tetrazole)	1.281(2)–1.284(2)	1.272
N-N (tetrazole)	1.372(1)–1.380(2)	1.369, 1.376
C=N (tetrazole)	1.312(2)	1.312
C-N (tetrazole)	1.364(2)–1.366(2)	1.375
C-C (tetrazole-alkyl)	1.483(2)	1.486
N-C (triazine)	1.313(2)–1.362(2)	1.346
B-B (carborane)	1.761(2)–1.790(2)	1.761
C-B (carborane)	1.691(2)–1.726(2)	1.718
C-C (carborane)	1.645(2)	1.641

**Table 2:** Computed HOMO and LUMO energies and the frontier molecular orbital energy gap values for compounds **9**–**11** and **15**–**17** in gas phase.

Compound	HOMO, eV	LUMO, eV	Δε, eV
**9**	−8.35	−3.40	4.95
**10**	−8.20	−3.16	5.15
**11**	−8.17	−3.12	5.05
**15**	−8.33	−3.75	4.57
**16**	−7.46	−2.97	4.49
**17**	−7.54	−3.05	4.49

**Table 3:** Calculated values of EOFs (∆H_298_) of compounds **9**–**11** and **15**–**17** (B3LYP 6-311 + G*).

Compound	∆H_298_, kcal/mol	Compound	∆H_298_, kcal/mol
**9**	181.90	**2** ^a^	198.09
**10**	165.70	**3** ^a^	181.78
**11**	165.11	**4** ^a^	131.44
**15**	217.07		
**16**	191.93		
**17**	176.69		

^a^ Magnitudes for **2**–**4** were taken from Yu. A. Borisov et al. [32].

**Table 4:** The calculated values of the detonation properties of compounds **9**–**11** and **15**–**17**.

Compound	N_g_	M_g,_ g/mol	Q, J/g	p_0_, g/cm^3^	D, km/s	P, GPa
**9**	13	376.24	2022.22	1.25	7.21	50.233
**10**	15	404.27	1716.54	1.53	7.61	76.286
**11**	17	432.30	1599.36	1.34	7.43	60.147
**15**	12	393.20	2311.36	1.36	12.14	54.32
**16**	12	393.20	2043.67	1.34	11.77	51.08
**17**	12	393.20	1881.40	1.36	11.53	49.01

**Table 5:** The calculated terahertz spectra of compounds **9**–**11** and **15**–**17**.

№	*ν*; THz	H; A^4^/AMU
**9**	0.6318, 0.8716, 1.2169, 1.4147, 1.4726, 2.1909, 2.5481, 3.0131	0.0013, 7.7105, 0.3268, 2.1826, 0.0605, 0.9840, 4.8842, 0.1518
**10**	0.4804, 0.5809, 0.7574, 0.8082, 1.371, 2.2101, 2.3605, 3.2013	0.0488, 3.4348, 7.2903, 1.1846, 2.0584, 1.2134, 4.6308, 0.2778
**11**	0.3168, 0.4199, 0.5698, 0.8613, 1.3083, 1.8885, 2.1009, 2.4997, 2.7895	0.8664, 0.5331, 2.6898, 4.5956, 1.7012, 2.2347, 2.0728, 1.4025, 0.3292
**15**	0.5478, 0.6870, 1.0455, 1.4581, 1.4918, 2.0758, 2.5671, 2.9889	0.4160, 0.2409, 4.6181, 0.5253, 0.7730, 0.6649, 5.1412, 0.1173
**16**	0.4267, 0.7548, 0.9538, 1.1091, 1.2381, 2.1343, 2.5848, 2.9406	0.4525, 0.2305, 0.8381, 10.9865, 1.6792, 4.3092, 4.8631, 0.0469
**17**	0.8451, 0.8923, 0.9935, 1.1842, 1.4842, 2.1501, 2.5904, 3.0237	0.3138, 0.0540, 0.4024, 11.5329, 1.3079, 3.5439, 4.9537, 0.0586

## Data Availability

CCDC 2177931, 2177932, 2177933 and 2177934 contain the supplementary crystallographic data for this paper. The data can be obtained free of charge from The Cambridge Crystallographic Data Centre via https://www.ccdc.cam.ac.uk/structures.

## Supplementary Materials

The following supporting information can be downloaded at: https://www.mdpi.com/article/10.3390/molecules27217484/s1, Figure S1. ^1^H NMR spectra of compound **9** in CD_3_CN; Figure S2. ^1^H NMR spectra of compound **9** in acetone-D_6_; Figure S3. ^1^H NMR spectra (500.13 MHz) of **9** in acetone-D_6_; Figure S4. ^1^H NMR spectra of **9** in acetone-D_6_ at various temperatures; Figure S5. ^13^C NMR spectra (125.76 MHz) of compound **9** in acetone-D_6_; Figure S6. ^11^B{^1^H} and ^11^B NMR spectra of compound **9** in acetone-D_6_; Figure S7. NOESY spectra of compound **9**; Figure S8. HMBC spectra of compound **9**; Figure S9. ^1^H NMR spectra of compound **10** in acetone-D_6_. Signals at δ = 1.02 ppm and δ = 3.39 ppm attributed to the protons of EtOAc; Figure S10. ^11^B{^1^H} and ^11^B NMR spectra of compound **10** in acetone-D_6_; Figure S11. ^1^H NMR of compound **11** in acetone-D_6_; Figure S12. ^11^B{^1^H} and ^11^B NMR spectra of compound **11** in acetone-D_6_; FigureS13. ^1^H NMR spectra of compound **19** in acetone-D_6_; Figure S14. ^11^B{^1^H} and ^11^B NMR spectra of compound **19** in acetone-D_6_; Figure S15. ^1^H NMR spectra of **20** in acetone-D_6_; Figure S16. ^11^B{^1^H} and ^11^B NMR spectra of compound **20** in acetone-D_6_; Figure S17. ^1^H NMR spectra of compound **15** in acetone-D_6_; Figure S18. ^11^B{^1^H} and ^11^B NMR spectra of compound **15** in acetone-D_6_; Figure S19. ^1^H NMR spectra of compound **16** in acetone-D_6_; Figure S20. ^11^B{^1^H} and ^11^B NMR spectra of compound **16** in acetone-D_6_; Figure S21. ^1^H NMR spectra of compound **17** in acetone-D_6_; Figure S22. ^11^B{^1^H} and ^11^B NMR spectra of compound **17** in acetone-D_6_; Table S1. X-ray data for compound **9**; Table S2. X-ray data for compound **10**; Table S3. X-ray data for compound **19**, Table S4. X-ray data for compound **20**; Table S5. Crystal data, data collection and structure refinement parameters for **9**, **10**, **19** and **20**; Figure S23. The fragment of double layer in the crystal of **9** (projection along the *b* axis); the shortened intermolecular contacts are shown with dotted lines. Table S6. Hydrogen bonds observed in the crystal of compound **9**; Table S7. Hydrogen bonds observed in the crystal of compound **10**; Figure S24. Fragment of crystal packing in **10** (projection along the *b* axis); Table S8. Hydrogen bonds observed in the crystal of compound **19**; Figure S25. Fragment of crystal packing in **19** (projection along the *c* axis); Table S9. Hydrogen bonds observed in the crystal of compound **20**; Figure S26. Fragment of crystal packing of **20** (projection along the *b* axis); Figure S27. Hirshfeld surfaces (a) and full fingerprint plots (b) of compound **9**; Figure S28. Individual interatomic contacts of compound **9** with percentage contribution in the crystal packing greater than 4.5%; Figure S29. Hirshfeld surfaces (a) and full fingerprint plots (b) of compound **10**; Figure S30. Individual interatomic contacts of compound **10** with a percentage contribution in the crystal packing greater than 4.5%; FigureS31. Hirshfeld surfaces (a) and full fingerprint plots (b) of compound **19**; Figure S32. Individual interatomic contacts of compound **19** with a percentage contribution in the crystal packing greater than 4.5%; Figure S33. Hirshfeld surfaces of compound **20**; Figure S34. Full fingerprint plots of compound **20**; Figure S35. Individual interatomic contacts of compound **20** (for two crystallographically independent molecules A and B) with a percentage contribution in the crystal packing greater than 4.5%; Figure S36. The supramolecular assembling of compound **9**. Hirshfeld surfaces mapped over d_norm_, generated from intermolecular H⋯H contacts. Figure S37. Supramolecular assembling of compound **9**. Hirshfeld surfaces mapped over d_norm_, generated from intermolecular N⋯H contacts or interactions; Table S10. Geometry structure of studied compounds; Table S11. The calculated values of total energy and entropy of compounds **9**–**11**, **15**–**17**, **19** and **25** (6-311 + G* basis set) in the gas phase; Table S12. Comparison of calculated and experimental EOF values of test molecules (kcal/mol).

## Sample Availability

Samples of the compounds are available from the authors.
