# Supplementary material for: Synthesis, Characterization and DFT Study of a New Family of High-Energy Compounds Based on s-Triazine, Carborane and Tetrazoles
Source: Molecules. 2022 Nov 2;27(21):7484. doi: 10.3390/molecules27217484 (PMC9656522; doi:10.3390/molecules27217484)

checkCIF/PLATON report

Structure factors have been supplied for datablock(s) a

THIS REPORT IS FOR GUIDANCE ONLY. IF USED AS PART OF A REVIEW PROCEDURE FOR PUBLICATION, IT SHOULD NOT REPLACE THE EXPERTISE OF AN EXPERIENCED CRYSTALLOGRAPHIC REFEREE.

No syntax errors found. CIF dictionary Interpreting this report

Datablock: a

```
Bond precision:   C-C = 0.0019 A          Wavelength=0.71073

Cell:            a=14.7936(5)    b=11.9606(4)         c=13.7459(5)
                 alpha=90        beta=111.6708(12)    gamma=90
Temperature:     100 K

                Calculated                        Reported
Volume          2260.30(14)                       2260.30(14)
Space group     P 21/c                            P 21/c
Hall group      -P 2ybc                           -P 2ybc
Moiety formula  C9 H18 B10 N12, C3 H6 O           C9 H18 B10 N12, C3 H6 O
Sum formula     C12 H24 B10 N12 O                 C12 H24 B10 N12 O
Mr              460.53                            460.53
Dx,g cm-3       1.353                             1.353
Z               4                                 4
Mu (mm-1)       0.086                             0.086
F000            952.0                             952.0
F000'           952.18
h,k,lmax        19,15,18                          19,15,18
Nref            5456                              5447
Tmin,Tmax       0.958,0.991                       0.854,0.991
Tmin'           0.958

Correction method= # Reported T Limits: Tmin=0.854 Tmax=0.991
AbsCorr = MULTI-SCAN

Data completeness= 0.998           Theta(max)= 27.997

R(reflections)= 0.0419( 4609)                      wR2(reflections)=
                                                   0.1038( 5447)
S = 1.047                  Npar= 368
```

The following ALERTS were generated. Each ALERT has the format
test-name_ALERT_alert-type_alert-level.
Click on the hyperlinks for more details of the test.

Alert level C

```
PLAT790_ALERT_4_C Centre of Gravity not Within Unit Cell: Resd.  #          1 Note
              C9 H18 B10 N12
PLAT906_ALERT_3_C Large K Value in the Analysis of Variance ......      2.460 Check
PLAT911_ALERT_3_C Missing FCF Refl Between Thmin & STh/L=    0.600          9 Report
PLAT913_ALERT_3_C Missing # of Very Strong Reflections in FCF ....          6 Note
```

Alert level G

```
PLAT343_ALERT_2_G Unusual sp?      Angle Range in Main Residue for         C1 Check
PLAT343_ALERT_2_G Unusual sp?      Angle Range in Main Residue for         C2 Check
PLAT367_ALERT_2_G Long?  C(sp?)-C(sp?) Bond  C1       - C2       .       1.64 Ang.
PLAT720_ALERT_4_G Number of Unusual/Non-Standard Labels ..........          6 Note
PLAT910_ALERT_3_G Missing # of FCF Reflection(s) Below Theta(Min).          1 Note
PLAT941_ALERT_3_G Average HKL Measurement Multiplicity ...........        4.2 Low
PLAT965_ALERT_2_G The SHELXL WEIGHT Optimisation has not Converged     Please Check
PLAT967_ALERT_5_G Note: Two-Theta Cutoff Value in Embedded .res ..       56.0 Degree
PLAT978_ALERT_2_G Number C-C Bonds with Positive Residual Density.          4 Info
```

0 **ALERT level A** = Most likely a serious problem - resolve or explain
0 **ALERT level B** = A potentially serious problem, consider carefully
4 **ALERT level C** = Check. Ensure it is not caused by an omission or oversight
9 **ALERT level G** = General information/check it is not something unexpected

0 ALERT type 1 CIF construction/syntax error, inconsistent or missing data
5 ALERT type 2 Indicator that the structure model may be wrong or deficient
5 ALERT type 3 Indicator that the structure quality may be low
2 ALERT type 4 Improvement, methodology, query or suggestion
1 ALERT type 5 Informative message, check

It is advisable to attempt to resolve as many as possible of the alerts in all categories. Often the minor alerts point to easily fixed oversights, errors and omissions in your CIF or refinement strategy, so attention to these fine details can be worthwhile. In order to resolve some of the more serious problems it may be necessary to carry out additional measurements or structure refinements. However, the purpose of your study may justify the reported deviations and the more serious of these should normally be commented upon in the discussion or experimental section of a paper or in the "special_details" fields of the CIF. checkCIF was carefully designed to identify outliers and unusual parameters, but every test has its limitations and alerts that are not important in a particular case may appear. Conversely, the absence of alerts does not guarantee there are no aspects of the results needing attention. It is up to the individual to critically assess their own results and, if necessary, seek expert advice.

Publication of your CIF in IUCr journals

A basic structural check has been run on your CIF. These basic checks will be run on all CIFs submitted for publication in IUCr journals (*Acta Crystallographica*, *Journal of Applied Crystallography*, *Journal of Synchrotron Radiation*); however, if you intend to submit to *Acta Crystallographica Section C* or *E* or *IUCrData*, you should make sure that full publication checks are run on the final version of your CIF prior to submission.

Publication of your CIF in other journals

Please refer to the *Notes for Authors* of the relevant journal for any special instructions relating to CIF submission.

PLATON version of 18/05/2022; check.def file version of 17/05/2022

Datablock a - ellipsoid plot

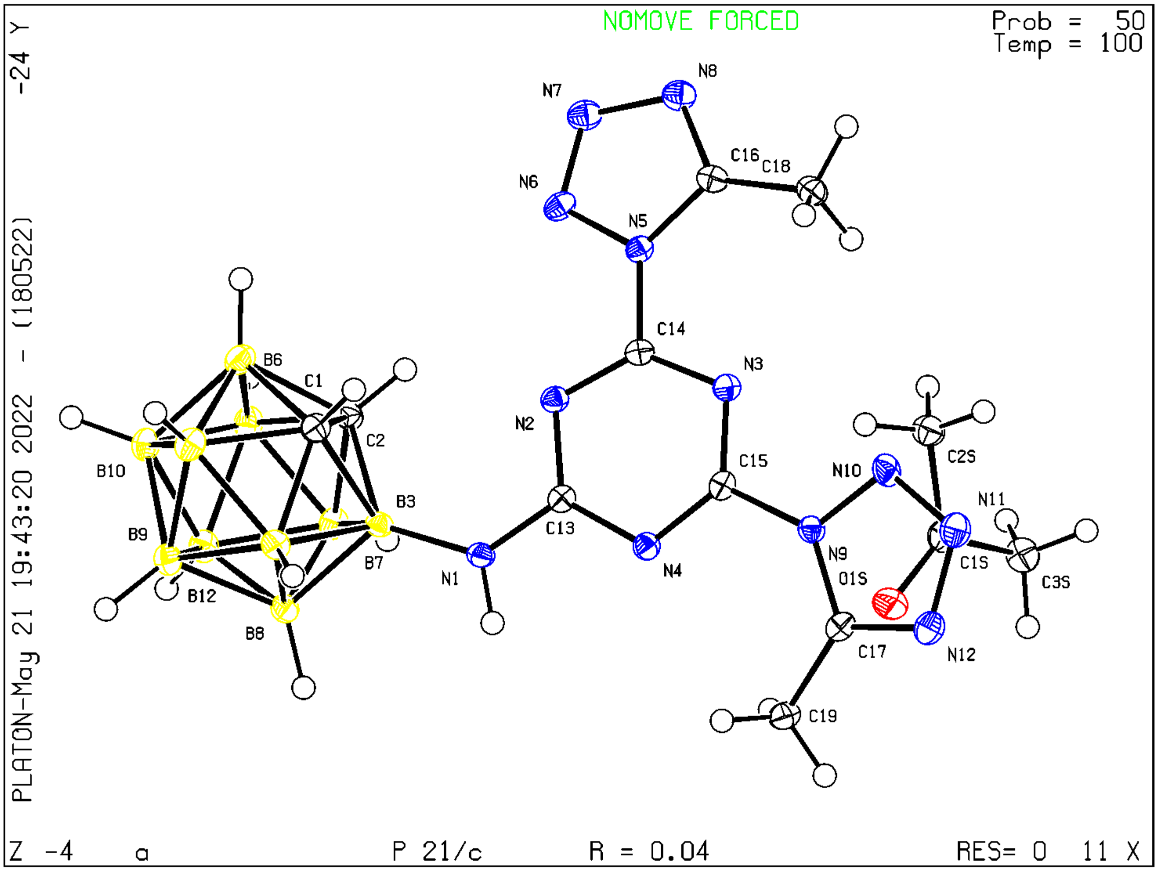

Supplement: Supplementary file 1 [file molecules-27-07484-s001.zip › checkcif_10.pdf]
