# Supplementary material for: Synthesis, Characterization and DFT Study of a New Family of High-Energy Compounds Based on s-Triazine, Carborane and Tetrazoles
Source: Molecules. 2022 Nov 2;27(21):7484. doi: 10.3390/molecules27217484 (PMC9656522; doi:10.3390/molecules27217484)

checkCIF/PLATON report

Structure factors have been supplied for datablock(s) abb

THIS REPORT IS FOR GUIDANCE ONLY. IF USED AS PART OF A REVIEW PROCEDURE FOR PUBLICATION, IT SHOULD NOT REPLACE THE EXPERTISE OF AN EXPERIENCED CRYSTALLOGRAPHIC REFEREE.

No syntax errors found.   CIF dictionary   Interpreting this report

Datablock: abb

```
Bond precision:   C-C = 0.0042 A          Wavelength=0.71073

Cell:               a=26.624(2)      b=28.969(2)      c=7.2719(6)
                    alpha=90         beta=90          gamma=90
Temperature:        120 K

                 Calculated                    Reported
Volume           5608.6(7)                     5608.4(8)
Space group      A b a 2                       A b a 2
Hall group       A 2 -2ac                      A 2 -2ac
Moiety formula   C19 H22 B10 N8, 2(C H4 O)     C19 H22 B10 N8, 2(C H4 O)
Sum formula      C21 H30 B10 N8 O2             C21 H30 B10 N8 O2
Mr               534.63                        534.63
Dx,g cm-3        1.266                         1.266
Z                8                             8
Mu (mm-1)        0.078                         0.078
F000             2224.0                        2224.0
F000'            2224.60
h,k,lmax         35,38,9                       35,38,9
Nref             6778[ 3657]                   6771
Tmin,Tmax        0.991,0.996                   0.865,0.996
Tmin'            0.958

Correction method= # Reported T Limits: Tmin=0.865 Tmax=0.996
AbsCorr = MULTI-SCAN

Data completeness= 1.85/1.00       Theta(max)= 27.998

R(reflections)= 0.0457( 5735)                          wR2(reflections)=
                                                       0.1046( 6771)
S = 1.075                  Npar= 428
```

The following ALERTS were generated. Each ALERT has the format
test-name_ALERT_alert-type_alert-level.
Click on the hyperlinks for more details of the test.

Alert level C

```
PLAT340_ALERT_3_C Low Bond Precision on  C-C Bonds ..............     0.0042 Ang.
```

Alert level G

```
PLAT343_ALERT_2_G Unusual sp?      Angle Range in Main Residue for         C1 Check
PLAT343_ALERT_2_G Unusual sp?      Angle Range in Main Residue for         C2 Check
PLAT367_ALERT_2_G Long?  C(sp?)-C(sp?) Bond  C1       - C2       .       1.63 Ang.
PLAT720_ALERT_4_G Number of Unusual/Non-Standard Labels .........          6 Note
PLAT883_ALERT_1_G No Info/Value for _atom_sites_solution_primary .     Please Do !
PLAT910_ALERT_3_G Missing # of FCF Reflection(s) Below Theta(Min).          3 Note
PLAT933_ALERT_2_G Number of HKL-OMIT Records in Embedded .res File          2 Note
PLAT960_ALERT_3_G Number of Intensities with I  <   - 2*sig(I) ...          3 Check
PLAT965_ALERT_2_G The SHELXL WEIGHT Optimisation has not Converged     Please Check
PLAT967_ALERT_5_G Note: Two-Theta Cutoff Value in Embedded .res ..       56.0 Degree
PLAT978_ALERT_2_G Number C-C Bonds with Positive Residual Density.          3 Info
```

0 **ALERT level A** = Most likely a serious problem - resolve or explain
0 **ALERT level B** = A potentially serious problem, consider carefully
1 **ALERT level C** = Check. Ensure it is not caused by an omission or oversight
11 **ALERT level G** = General information/check it is not something unexpected

1 ALERT type 1 CIF construction/syntax error, inconsistent or missing data
6 ALERT type 2 Indicator that the structure model may be wrong or deficient
3 ALERT type 3 Indicator that the structure quality may be low
1 ALERT type 4 Improvement, methodology, query or suggestion
1 ALERT type 5 Informative message, check

It is advisable to attempt to resolve as many as possible of the alerts in all categories. Often the minor alerts point to easily fixed oversights, errors and omissions in your CIF or refinement strategy, so attention to these fine details can be worthwhile. In order to resolve some of the more serious problems it may be necessary to carry out additional measurements or structure refinements. However, the purpose of your study may justify the reported deviations and the more serious of these should normally be commented upon in the discussion or experimental section of a paper or in the "special_details" fields of the CIF. checkCIF was carefully designed to identify outliers and unusual parameters, but every test has its limitations and alerts that are not important in a particular case may appear. Conversely, the absence of alerts does not guarantee there are no aspects of the results needing attention. It is up to the individual to critically assess their own results and, if necessary, seek expert advice.

Publication of your CIF in IUCr journals

A basic structural check has been run on your CIF. These basic checks will be run on all CIFs submitted for publication in IUCr journals (*Acta Crystallographica*, *Journal of Applied Crystallography*, *Journal of Synchrotron Radiation*); however, if you intend to submit to *Acta Crystallographica Section C* or *E* or *IUCrData*, you should make sure that full publication checks are run on the final version of your CIF prior to submission.

Publication of your CIF in other journals

Please refer to the *Notes for Authors* of the relevant journal for any special instructions relating to CIF submission.

PLATON version of 18/05/2022; check.def file version of 17/05/2022

Datablock abb - ellipsoid plot

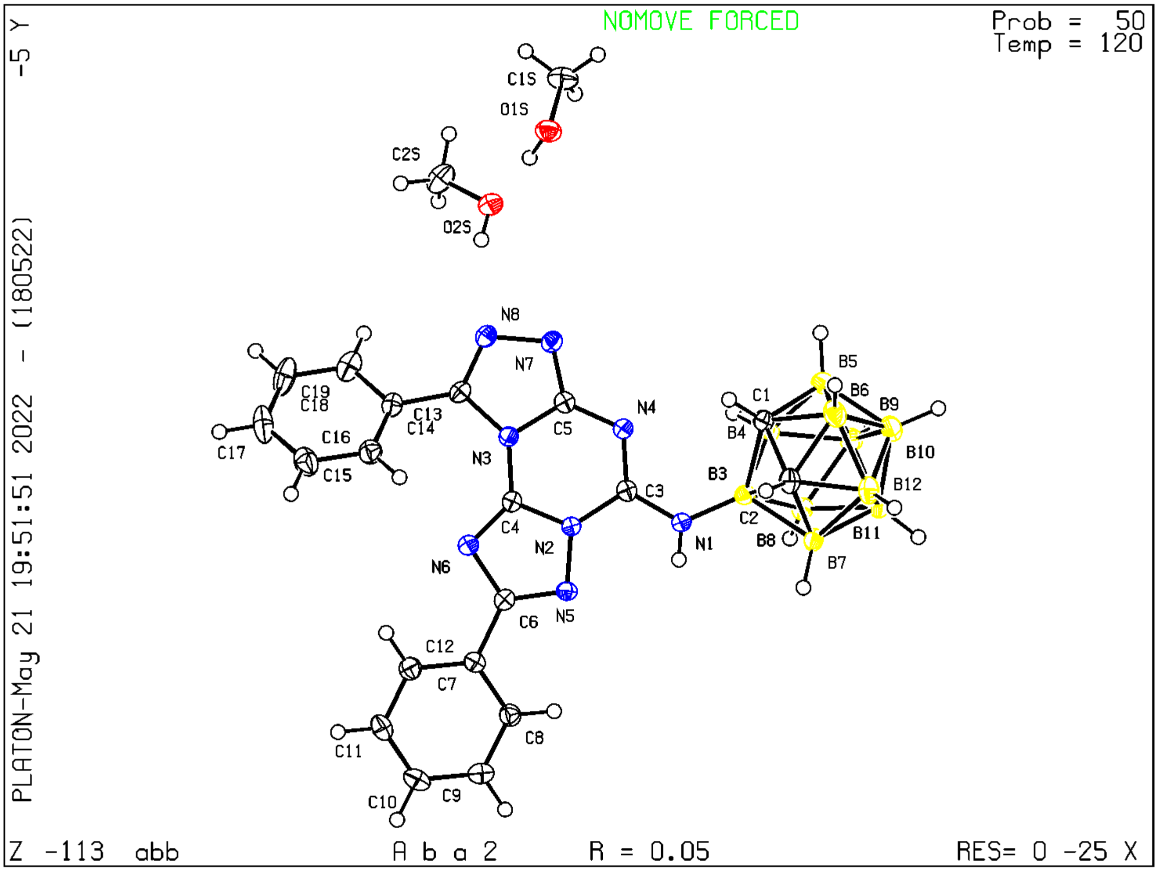

Supplement: Supplementary file 1 [file molecules-27-07484-s001.zip › checkcif_19.pdf]
