# Supplementary material for: Synthesis, Characterization and DFT Study of a New Family of High-Energy Compounds Based on s-Triazine, Carborane and Tetrazoles
Source: Molecules. 2022 Nov 2;27(21):7484. doi: 10.3390/molecules27217484 (PMC9656522; doi:10.3390/molecules27217484)

checkCIF/PLATON report

Structure factors have been supplied for datablock(s) aa_sq

THIS REPORT IS FOR GUIDANCE ONLY. IF USED AS PART OF A REVIEW PROCEDURE FOR PUBLICATION, IT SHOULD NOT REPLACE THE EXPERTISE OF AN EXPERIENCED CRYSTALLOGRAPHIC REFEREE.

No syntax errors found. CIF dictionary Interpreting this report

Datablock: aa_sq

```
Bond precision:   C-C = 0.0076 A          Wavelength=0.71073

Cell:           a=14.089(2)        b=15.442(3)      c=16.375(3)
                alpha=109.645(4)   beta=95.426(4)   gamma=116.641(4)
Temperature:    120 K
```

	Calculated	Reported
Volume	2868.9(9)	2868.8(9)
Space group	P -1	P -1
Hall group	-P 1	-P 1
Moiety formula	C19 H24 B10 N10 O [+ solvent]	C19 H24 B10 N10 O, C H2 Cl2
Sum formula	C19 H24 B10 N10 O [+ solvent]	C20 H26 B10 Cl2 N10 O
Mr	516.58	601.51
Dx,g cm-3	1.196	1.393
Z	4	4
Mu (mm-1)	0.074	0.264
F000	1064.0	1232.0
F000'	1064.25	
h,k,lmax	17,19,20	17,19,20
Nref	11277	11263
Tmin,Tmax	0.924,0.987	0.847,0.987
Tmin'	0.912	

```
Correction method= # Reported T Limits: Tmin=0.847 Tmax=0.987
AbsCorr = MULTI-SCAN

Data completeness= 0.999           Theta(max)= 25.999
```

R(reflections)= 0.0720(5302) wR2(reflections)= 0.2002(11263)

S = 0.984 Npar= 721

The following ALERTS were generated. Each ALERT has the format
test-name_ALERT_alert-type_alert-level.
Click on the hyperlinks for more details of the test.

Alert level C

```
RINTA01_ALERT_3_C  The value of Rint is greater than 0.12
            Rint given   0.120
PLAT026_ALERT_3_C Ratio Observed / Unique Reflections (too) Low ..        47% Check
PLAT213_ALERT_2_C Atom C10B             has ADP max/min Ratio .....        3.2 prolat
PLAT220_ALERT_2_C NonSolvent   Resd 2  C   Ueq(max)/Ueq(min) Range        4.6 Ratio
PLAT222_ALERT_3_C NonSolvent Resd 2  H   Uiso(max)/Uiso(min) Range        4.1 Ratio
PLAT234_ALERT_4_C Large Hirshfeld Difference C9B      --C10B     .       0.17 Ang.
PLAT241_ALERT_2_C High   'MainMol' Ueq as Compared to Neighbors of       C10B Check
PLAT340_ALERT_3_C Low Bond Precision on  C-C Bonds ..............     0.0076 Ang.
PLAT414_ALERT_2_C Short Intra D-H..H-X       H6AA      ..H12C     .       1.94 Ang.
                                                  x,y,z  =      1_555 Check
PLAT420_ALERT_2_C D-H Bond Without Acceptor  N1A      --H1AA     .     Please Check
PLAT420_ALERT_2_C D-H Bond Without Acceptor  N6A      --H6AA     .     Please Check
PLAT420_ALERT_2_C D-H Bond Without Acceptor  N1B      --H1BA     .     Please Check
PLAT905_ALERT_3_C Negative K value in the Analysis of Variance ...     -4.913 Report
PLAT910_ALERT_3_C Missing # of FCF Reflection(s) Below Theta(Min).          7 Note
PLAT911_ALERT_3_C Missing FCF Refl Between Thmin & STh/L=    0.600          8 Report
```

Alert level G

```
FORMU01_ALERT_2_G  There is a discrepancy between the atom counts in the
            _chemical_formula_sum and the formula from the _atom_site* data.
            Atom count from _chemical_formula_sum:C20 H26 B10 Cl2 N10 O1
            Atom count from the _atom_site data:  C19 H24 B10 N10 O1
CELLZ01_ALERT_1_G Difference between formula and atom_site contents detected.
CELLZ01_ALERT_1_G ALERT: Large difference may be due to a
            symmetry error - see SYMMG tests
           From the CIF: _cell_formula_units_Z    4
           From the CIF: _chemical_formula_sum  C20 H26 B10 Cl2 N10 O
           TEST: Compare cell contents of formula and atom_site data

           atom    Z*formula  cif sites diff
           C         80.00     76.00    4.00
           H        104.00     96.00    8.00
           B         40.00     40.00    0.00
           Cl         8.00      0.00    8.00
           N         40.00     40.00    0.00
           O          4.00      4.00    0.00
PLAT007_ALERT_5_G Number of Unrefined Donor-H Atoms ..............          6 Report
PLAT041_ALERT_1_G Calc. and Reported SumFormula    Strings  Differ     Please Check
PLAT051_ALERT_1_G Mu(calc) and Mu(CIF) Ratio Differs from 1.0 by .      72.11 %
PLAT154_ALERT_1_G The s.u.'s on the Cell Angles are Equal ..(Note)      0.004 Degree
PLAT343_ALERT_2_G Unusual sp?      Angle Range in Main Residue for        C1A Check
PLAT343_ALERT_2_G Unusual sp?      Angle Range in Main Residue for        C2A Check
PLAT343_ALERT_2_G Unusual sp?      Angle Range in Main Residue for        C1B Check
```

```
PLAT343_ALERT_2_G Unusual sp?      Angle Range in Main Residue for        C2B Check
PLAT367_ALERT_2_G Long?  C(sp?)-C(sp?) Bond  C1A      - C2A      .       1.64 Ang.
PLAT367_ALERT_2_G Long?  C(sp?)-C(sp?) Bond  C1B      - C2B      .       1.63 Ang.
PLAT606_ALERT_4_G Solvent Accessible VOID(S) in Structure .......           ! Info
PLAT720_ALERT_4_G Number of Unusual/Non-Standard Labels ..........         10 Note
PLAT869_ALERT_4_G ALERTS Related to the Use of SQUEEZE  Suppressed          ! Info
PLAT933_ALERT_2_G Number of HKL-OMIT Records in Embedded .res File         12 Note
PLAT941_ALERT_3_G Average HKL Measurement Multiplicity ..........         2.5 Low
PLAT960_ALERT_3_G Number of Intensities with I  <   - 2*sig(I) ...          8 Check
PLAT967_ALERT_5_G Note: Two-Theta Cutoff Value in Embedded .res ..       52.0 Degree
PLAT978_ALERT_2_G Number C-C Bonds with Positive Residual Density.          0 Info
```

```
 0 ALERT level A = Most likely a serious problem - resolve or explain
 0 ALERT level B = A potentially serious problem, consider carefully
15 ALERT level C = Check. Ensure it is not caused by an omission or oversight
21 ALERT level G = General information/check it is not something unexpected

 5 ALERT type 1 CIF construction/syntax error, inconsistent or missing data
16 ALERT type 2 Indicator that the structure model may be wrong or deficient
 9 ALERT type 3 Indicator that the structure quality may be low
 4 ALERT type 4 Improvement, methodology, query or suggestion
 2 ALERT type 5 Informative message, check
```

It is advisable to attempt to resolve as many as possible of the alerts in all categories. Often the minor alerts point to easily fixed oversights, errors and omissions in your CIF or refinement strategy, so attention to these fine details can be worthwhile. In order to resolve some of the more serious problems it may be necessary to carry out additional measurements or structure refinements. However, the purpose of your study may justify the reported deviations and the more serious of these should normally be commented upon in the discussion or experimental section of a paper or in the "special_details" fields of the CIF. checkCIF was carefully designed to identify outliers and unusual parameters, but every test has its limitations and alerts that are not important in a particular case may appear. Conversely, the absence of alerts does not guarantee there are no aspects of the results needing attention. It is up to the individual to critically assess their own results and, if necessary, seek expert advice.

Publication of your CIF in IUCr journals

A basic structural check has been run on your CIF. These basic checks will be run on all CIFs submitted for publication in IUCr journals (*Acta Crystallographica*, *Journal of Applied Crystallography*, *Journal of Synchrotron Radiation*); however, if you intend to submit to *Acta Crystallographica Section C* or *E* or *IUCrData*, you should make sure that full publication checks are run on the final version of your CIF prior to submission.

Publication of your CIF in other journals

Please refer to the *Notes for Authors* of the relevant journal for any special instructions relating to CIF submission.

PLATON version of 18/05/2022; check.def file version of 17/05/2022

Datablock aa_sq - ellipsoid plot

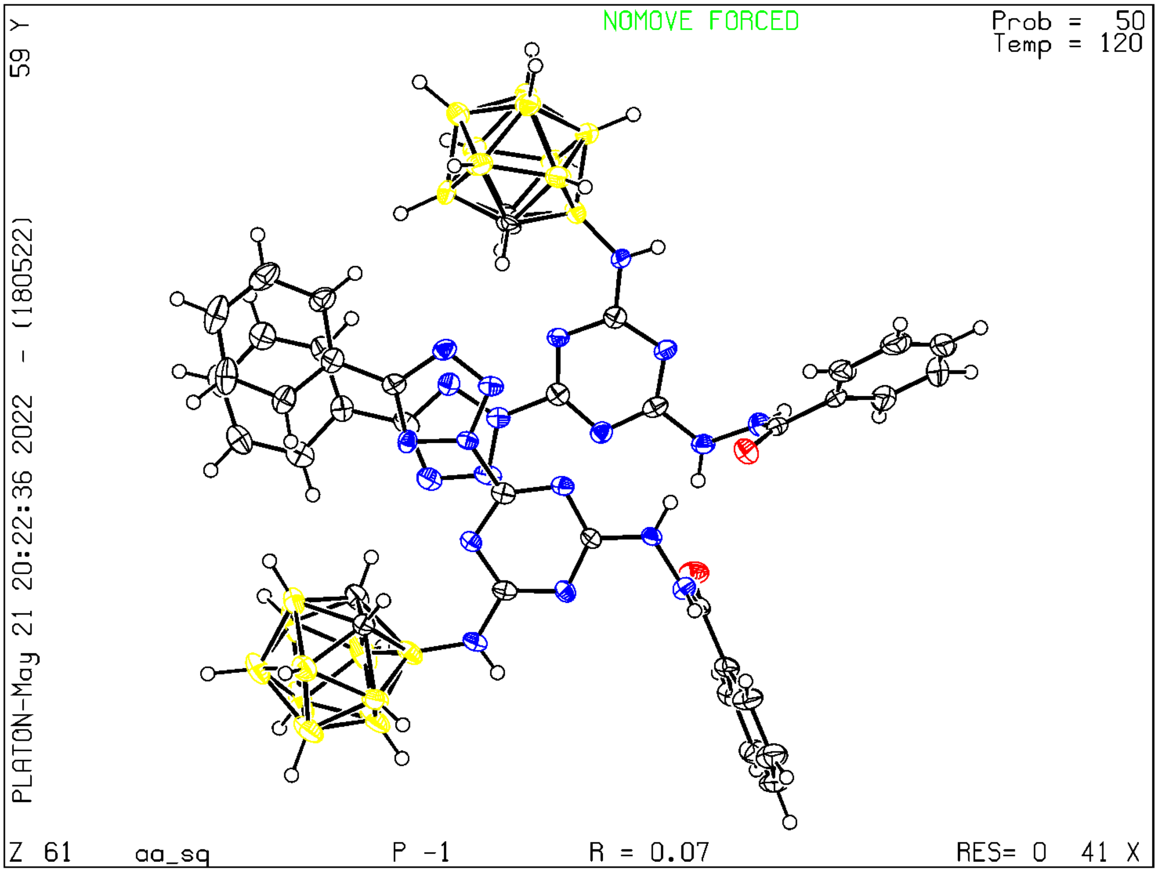

Supplement: Supplementary file 1 [file molecules-27-07484-s001.zip › checkcif_20.pdf]
