# Supplementary material for: Synthesis, Characterization and DFT Study of a New Family of High-Energy Compounds Based on s-Triazine, Carborane and Tetrazoles
Source: Molecules. 2022 Nov 2;27(21):7484. doi: 10.3390/molecules27217484 (PMC9656522; doi:10.3390/molecules27217484)
Supplement: Supplementary file 1 [file molecules-27-07484-s001.zip › molecules-1978746-supplementary.pdf]

molecules

Supplementary Materials

Synthesis, Characterization and DFT Study of a New Family of High Energy Compounds Based on *s*-Triazine, Carborane and Tetrazoles

Anton V. Makarenkov [1], Sergey S. Kiselev [1], Elena G. Kononova [1], Fedor M. Dolgushin [2], Alexander S. Peregudov [1], Yurii A. Borisov [1], Valentina A. Ol'shevskaya [1,*]

[1] A.N.Nesmeyanov Institute of Organoelement Compounds, Russian Academy of Sciences, 28, bld. 1 Vavilova street, 119334 Moscow, Russian
[2] N. S. Kurnakov Institute of General and Inorganic Chemistry, Russian Academy of Sciences, 31 Leninsky Prosp., 119071 Moscow, Russian
* Correspondence: olshevsk@ineos.ac.ru

Recommendation

All the prepared compounds are stable during the preparation and isolation but they are rather sensitive to the open fire.

Table of contents

1. NMR spectra
1.1. NMR spectra of compounds **9-11** 2-8
1.2. NMR spectra of compounds **19-20** 9-10
1.3 NMR spectra of compounds **15-17** 11-13
2. X-ray crystallographic data of compounds **9**, **10**, **19**, **20** 14-20
3. Hirshfeld surface analysis of compounds **9**, **10**, **19**, **20** 20-26
4. Calculated geometry and electronic structure for compounds **9-11**, **15-17**, **19** and **25** 27-30
5. Reference 30

NMR spectra of compounds 9-11, 19, 20.

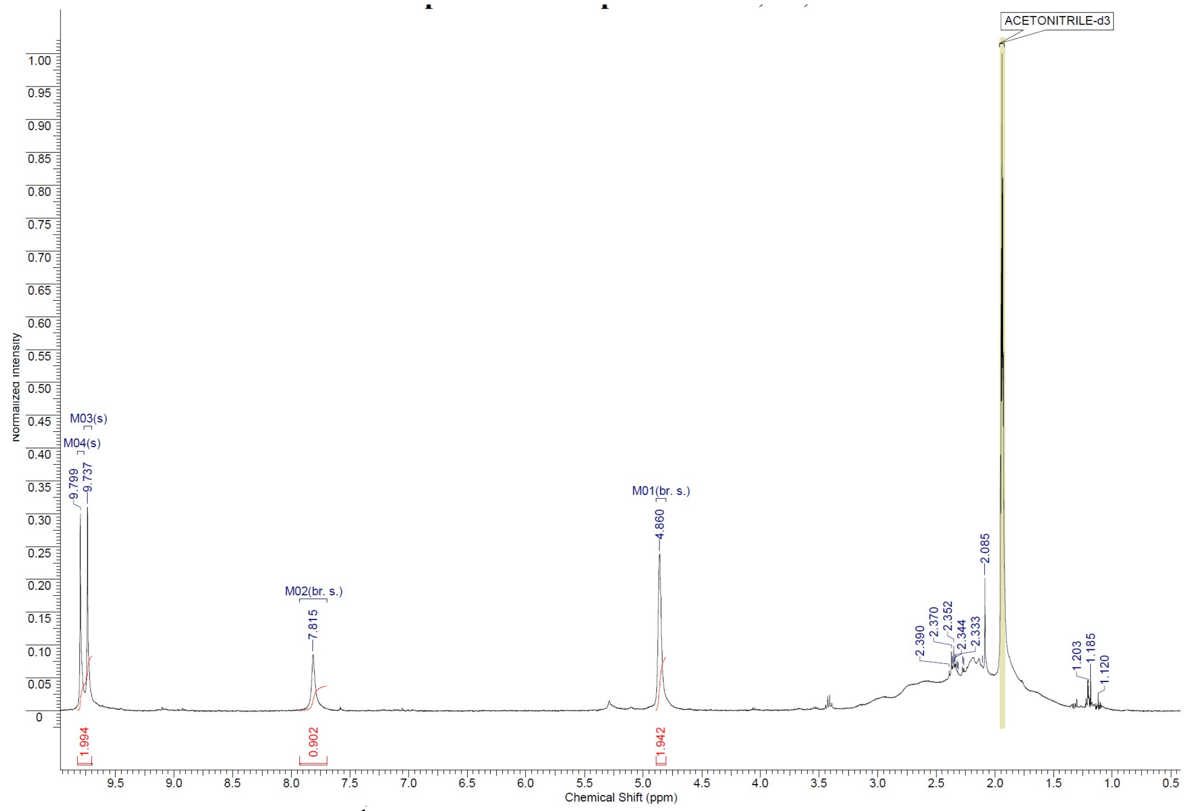

Figure S1. ^{1}H NMR spectra of compound **9** in acetonitrile-D_3

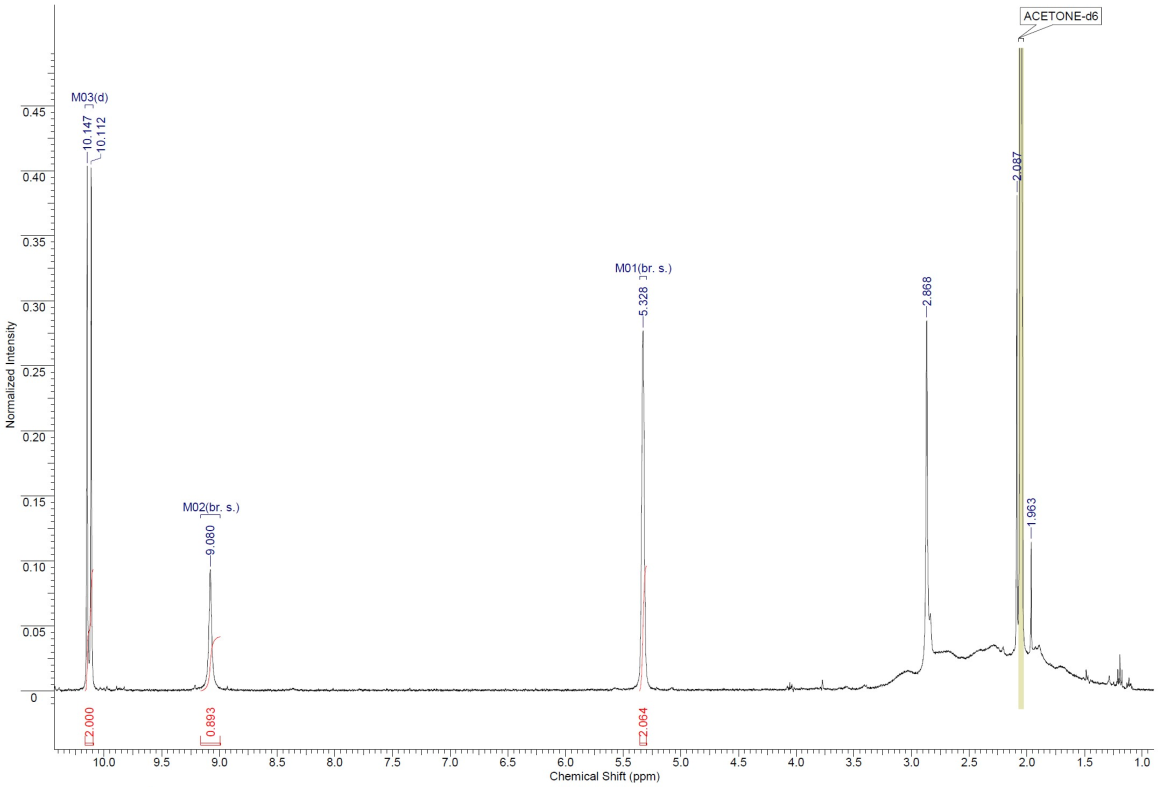

Figure S2. ^{1}H NMR spectra of compound **9** in acetone-D_6. The signal at $\delta = 2.88$ ppm belong to water protons.

After heating of the sample in the ampoule, a significant decrease in the integral intensity of the signal of NH proton was observed, apparently associated with the exchange processes between compound **9**, acetone and acetone-D_6 (Figure S3 and Figure S4).

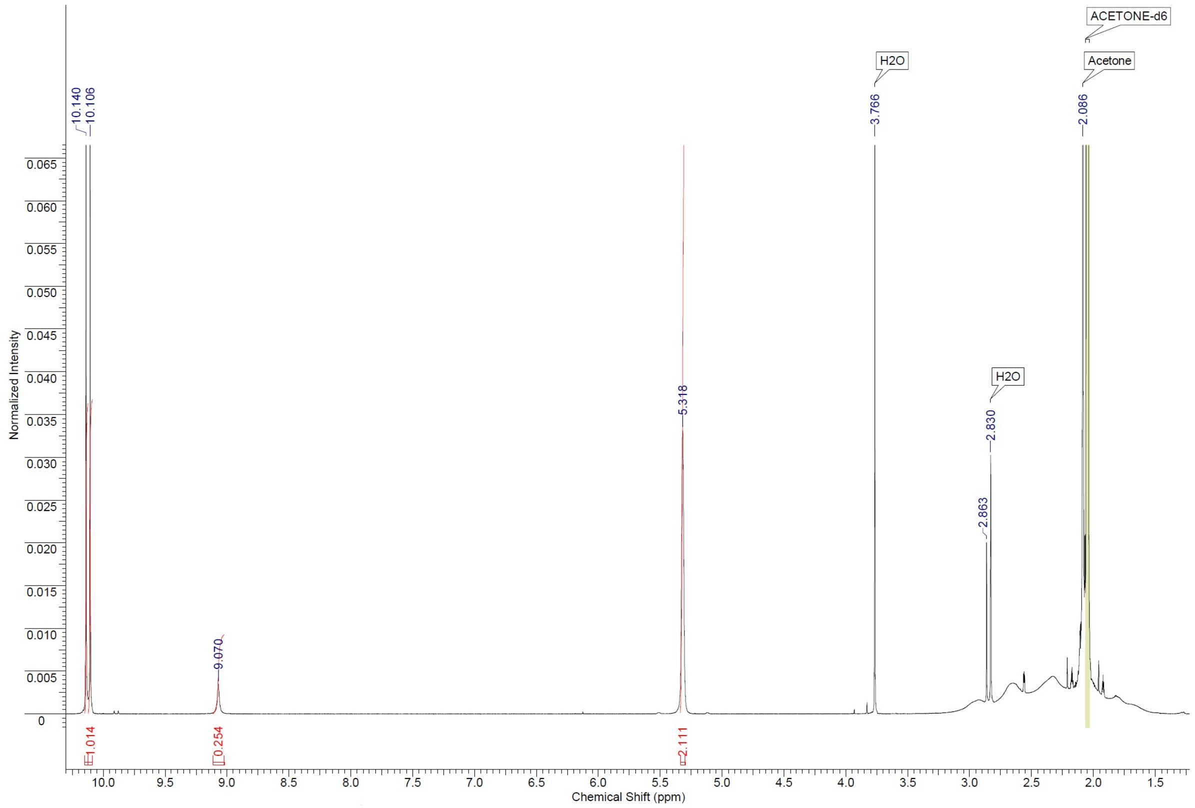

Figure S3. ^{1}H NMR Spectra (500.13 MHz) of **9** in acetone-D_6.

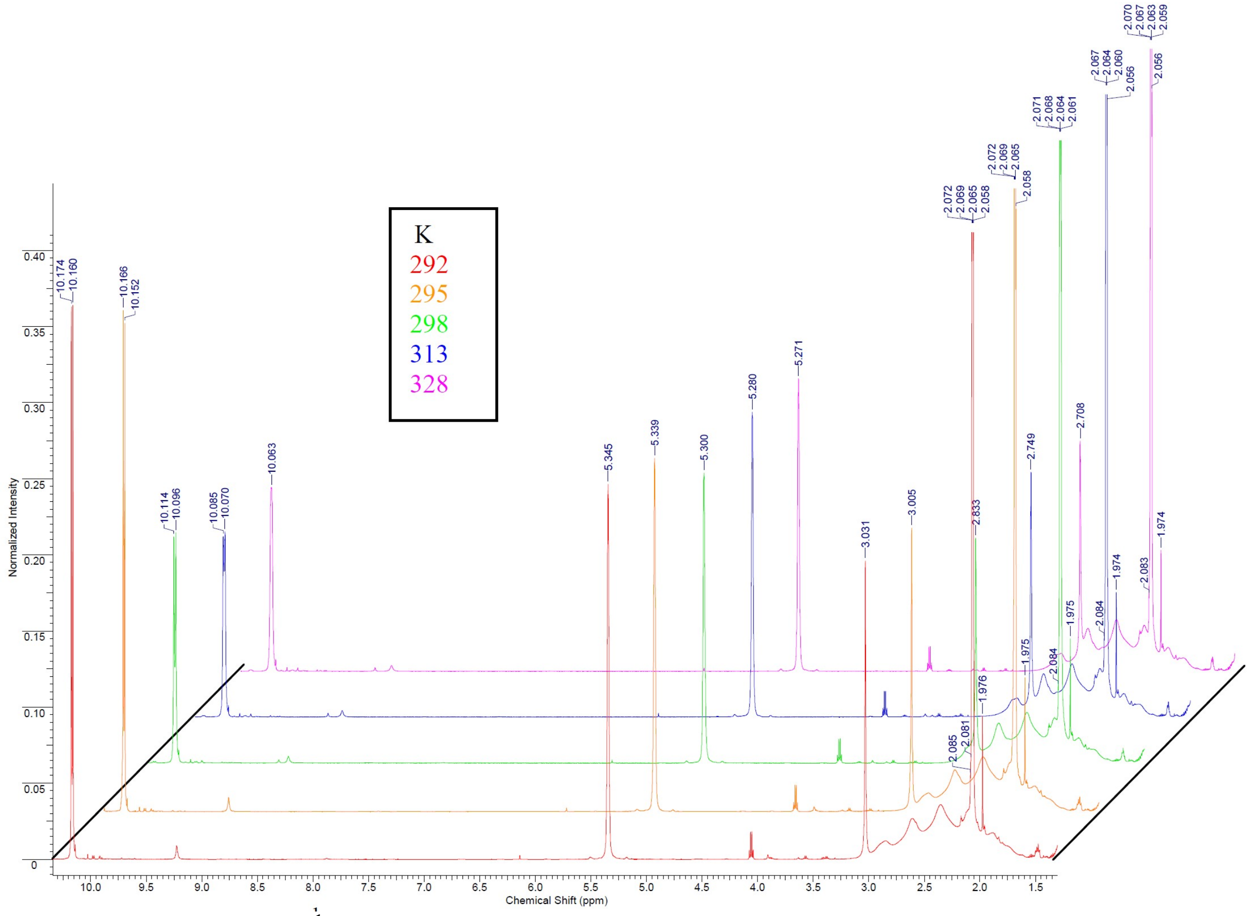

Figure S4. ^{1}H NMR spectra of **9** in acetone-D_6 at various temperatures.

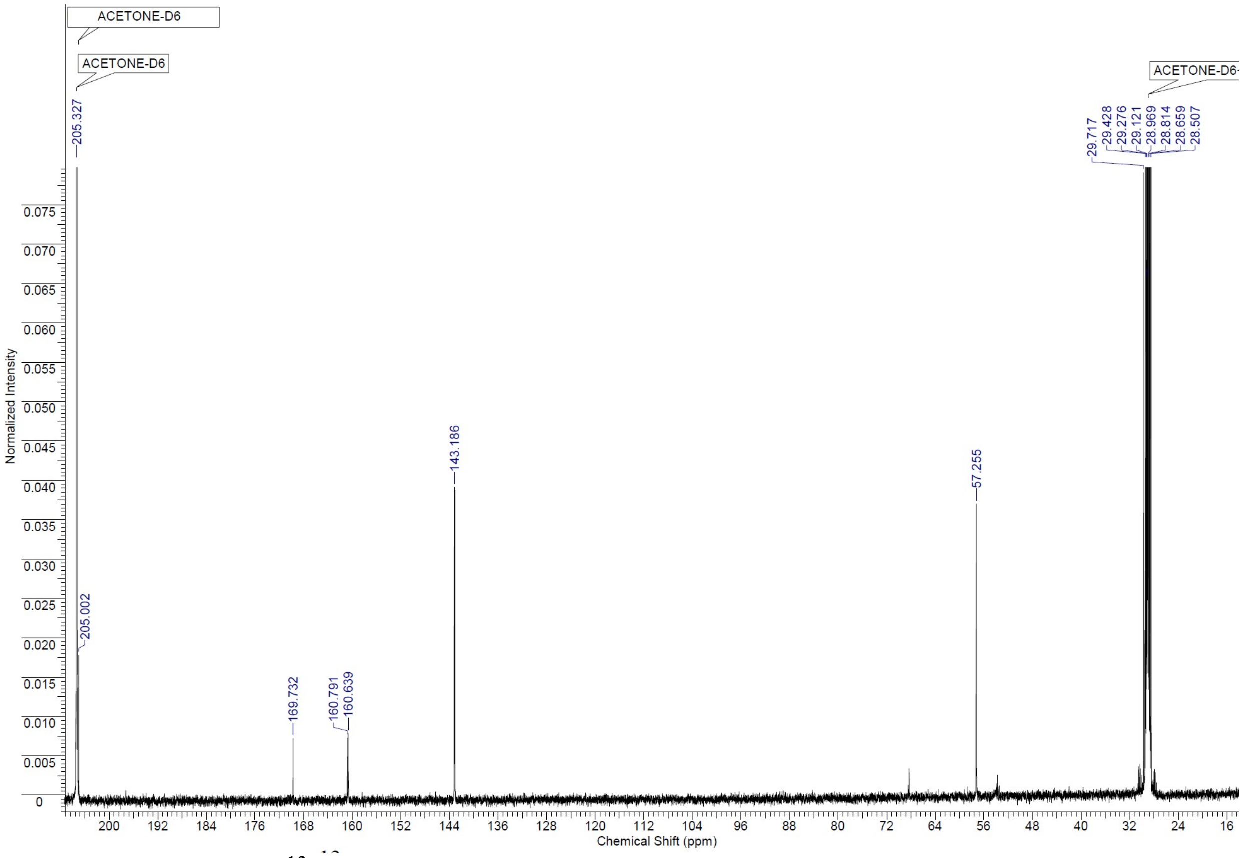

Figure S5. ^{13}C NMR Spectra (125.76 MHz) of compound **9** in acetone-D$_6$.

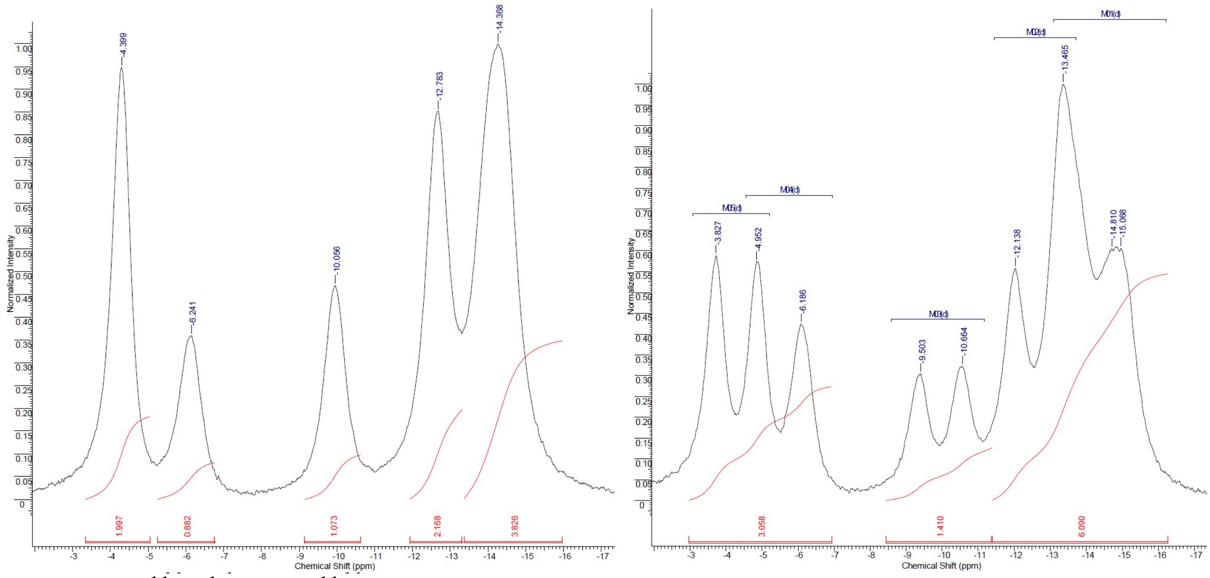

Figure S6. $^{11}B\{^1H\}$ and ^{11}B NMR spectra of compound **9** in acetone-D$_6$

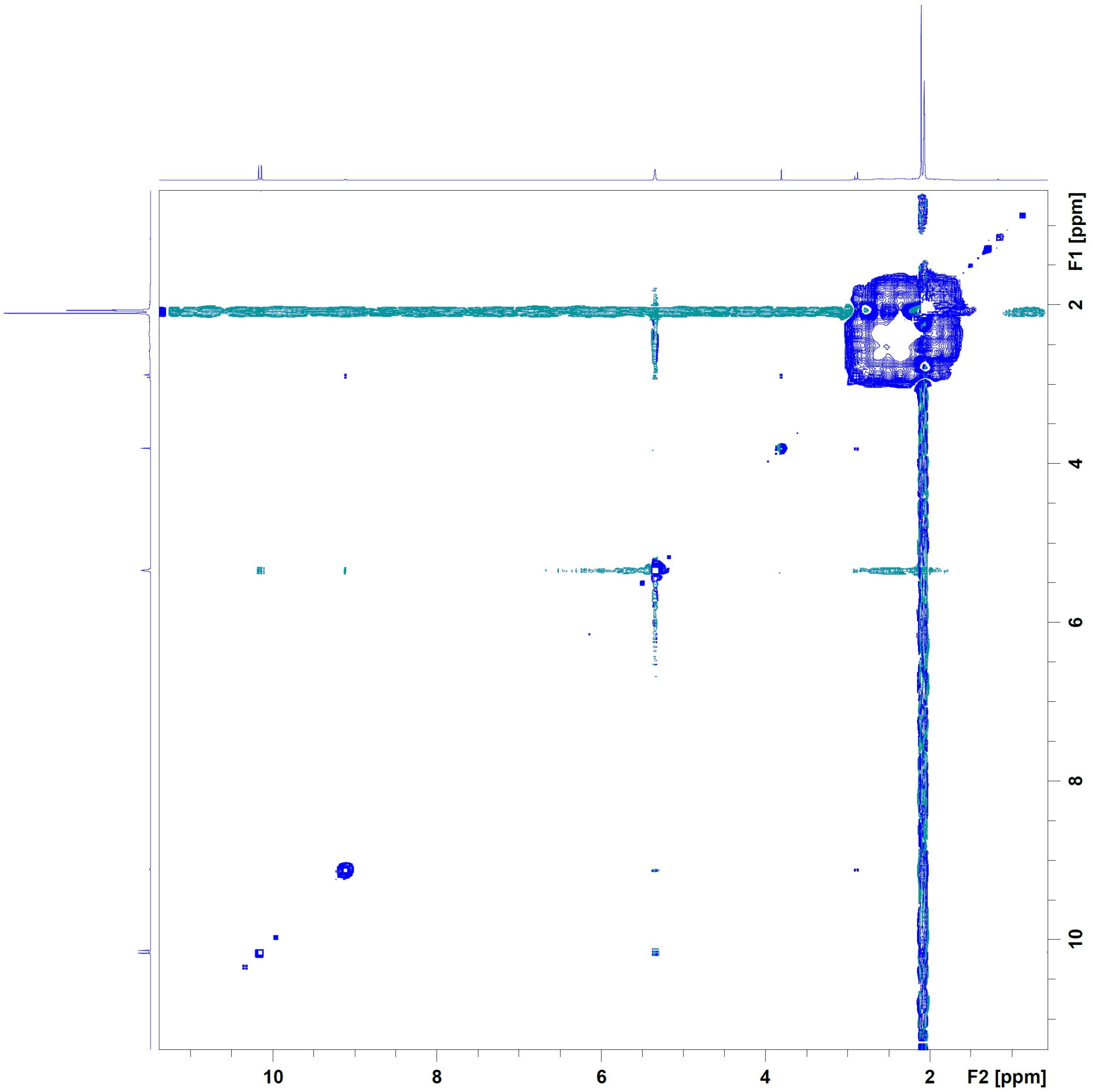

Figure S7. NOESY spectra of compound **9**.

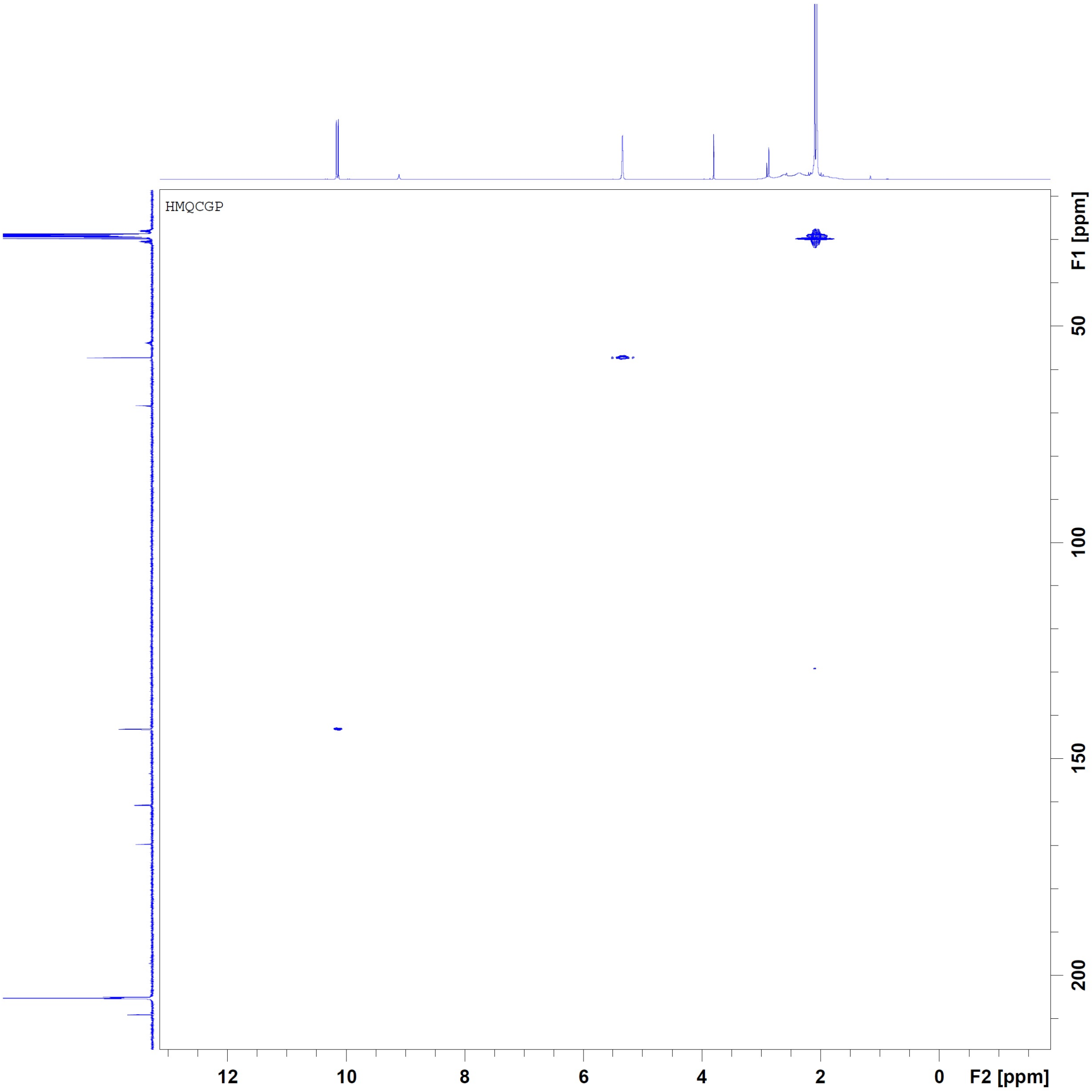

Figure S8. HMBC spectra of compound **9**.

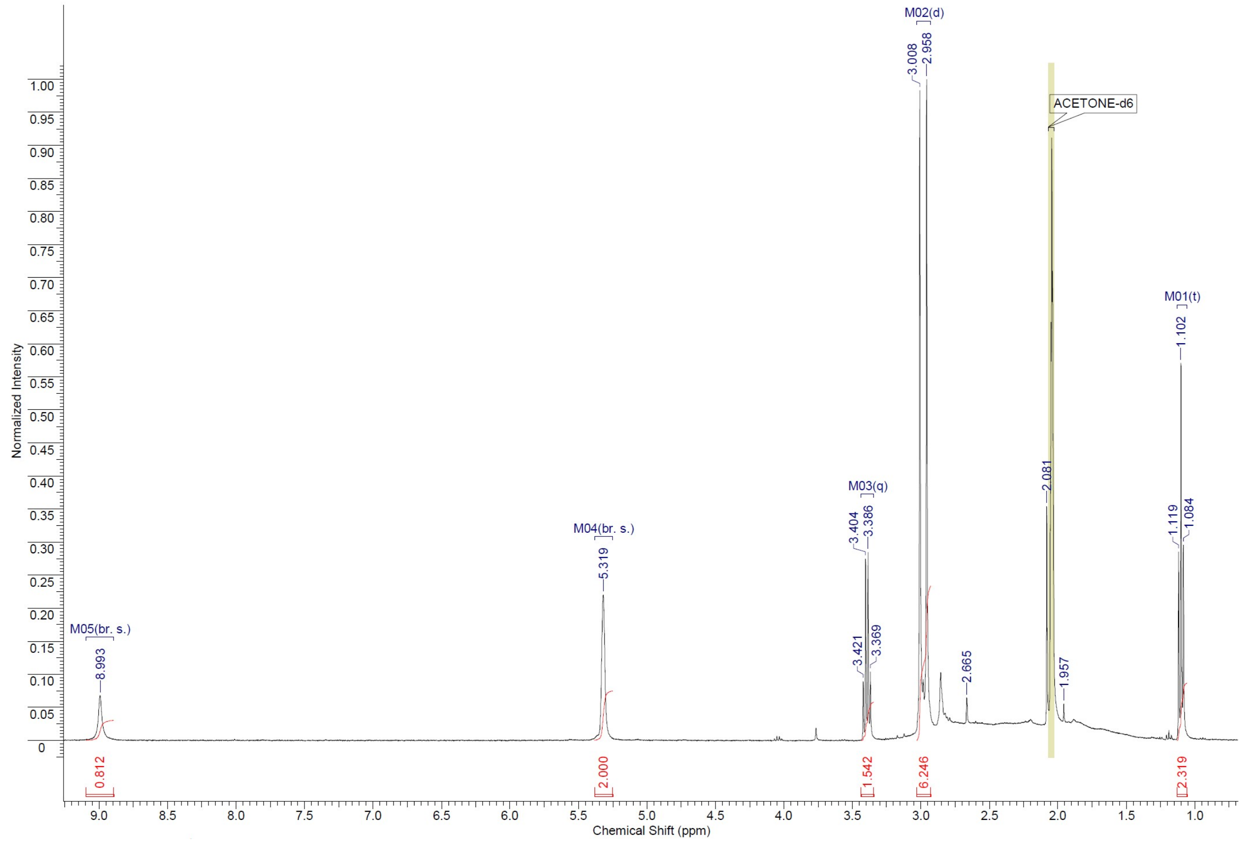

Figure S9. ^{1}H NMR spectra of compound **10** in acetone-D_6. Signals at δ = 1.02 ppm and δ = 3.39 ppm attributed to the protons of EtOAc.

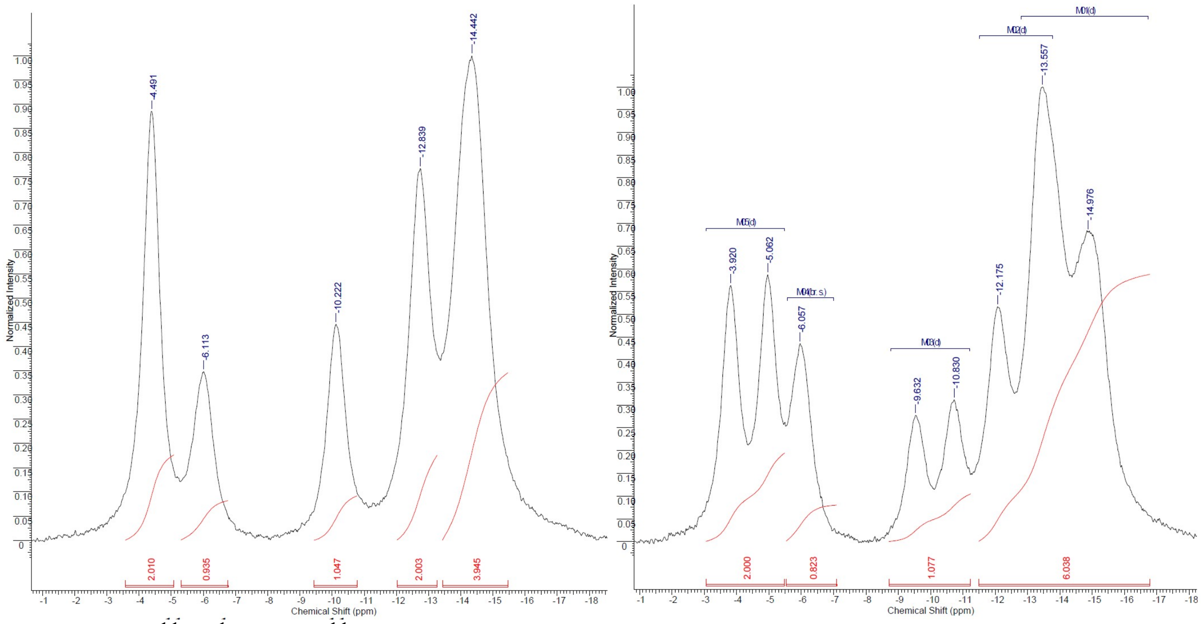

Figure S10. ^{11}B{^{1}H} and ^{11}B NMR Spectra of compound **10** in acetone-D_6.

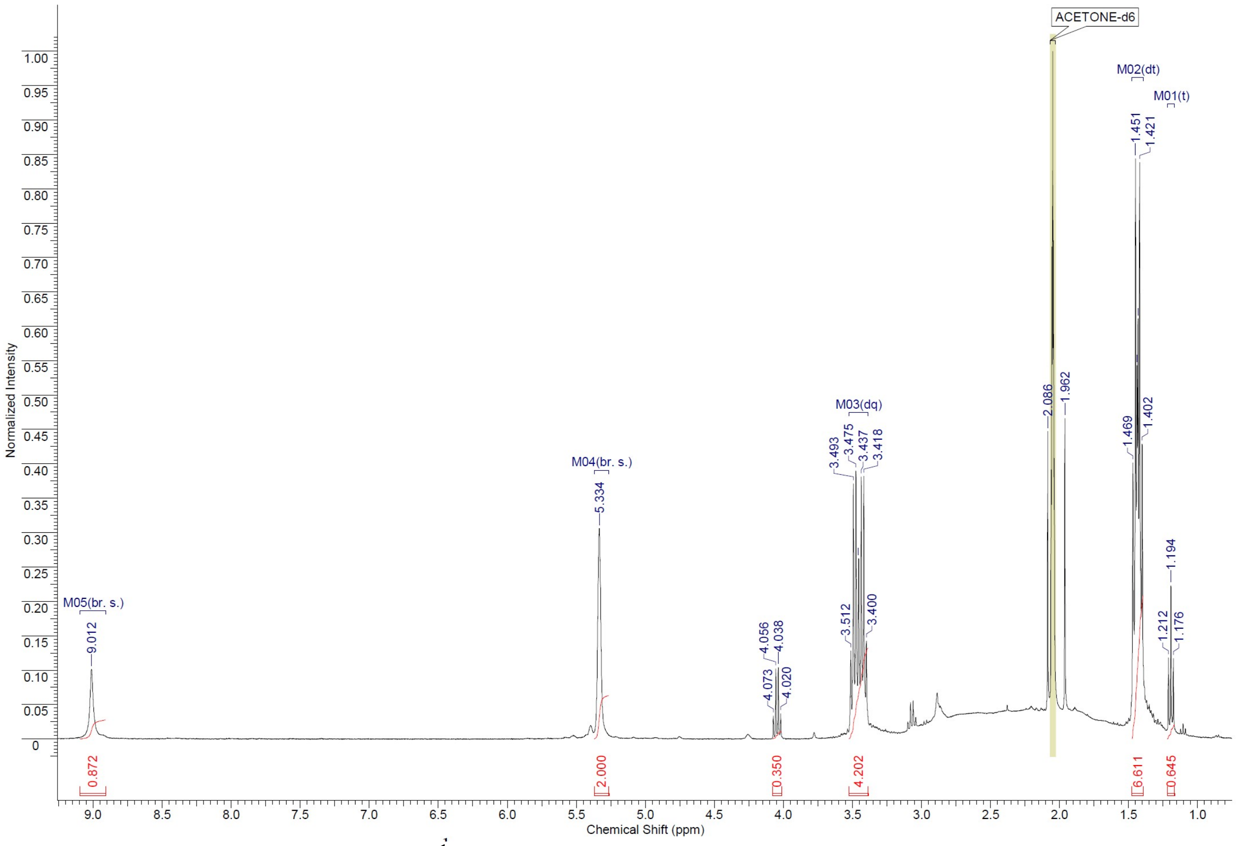

Figure S11. ^{1}H NMR of compound **11** in acetone-D$_6$.

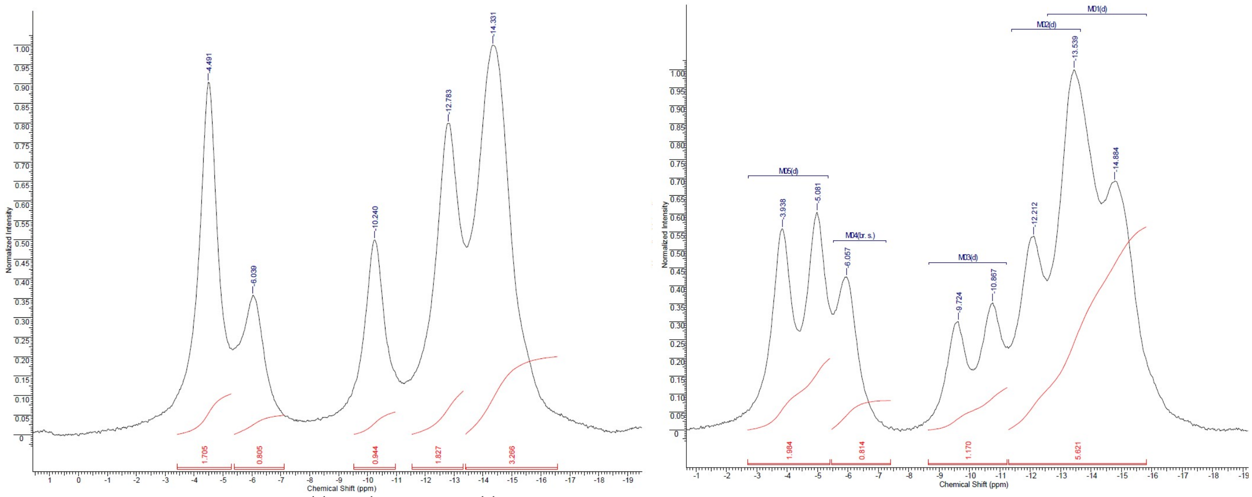

Figure S12. ^{11}B{^{1}H} and ^{11}B NMR Spectra of compound **11** in acetone-D$_6$.

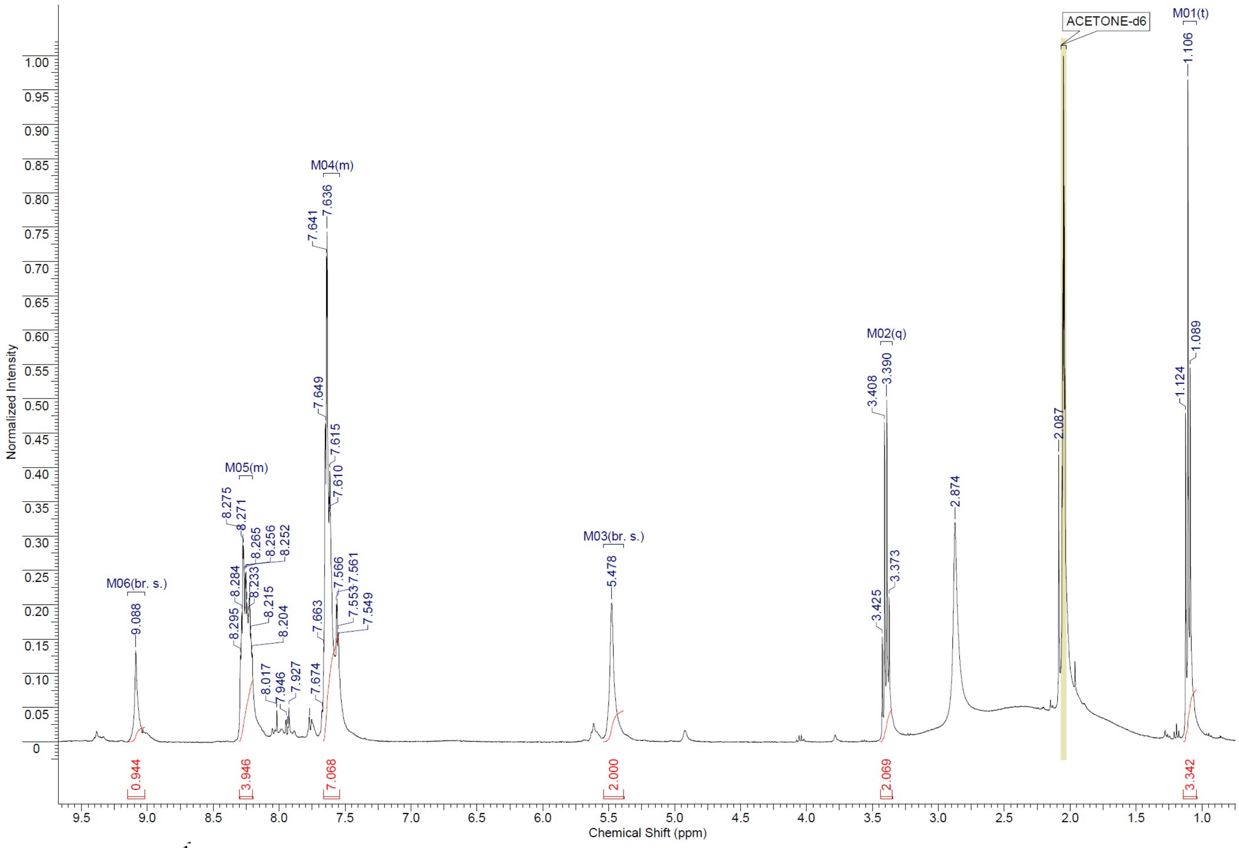

Figure S13. ^{1}H NMR spectra of compound **19** in acetone-D$_6$. Signals at δ = 1.02 ppm and δ = 3.39 ppm attributed to the protons of EtOAc which formed the stable complex (1:1) with compound **19**.

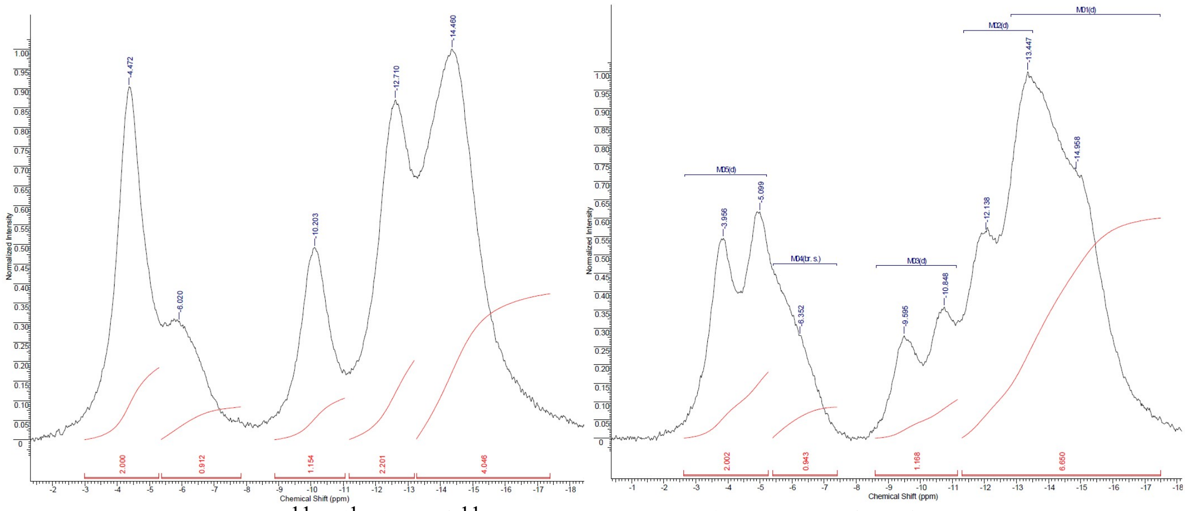

Figure S14. $^{11}B\{^{1}H\}$ and ^{11}B NMR spectra of compound **19** in acetone-D$_6$.

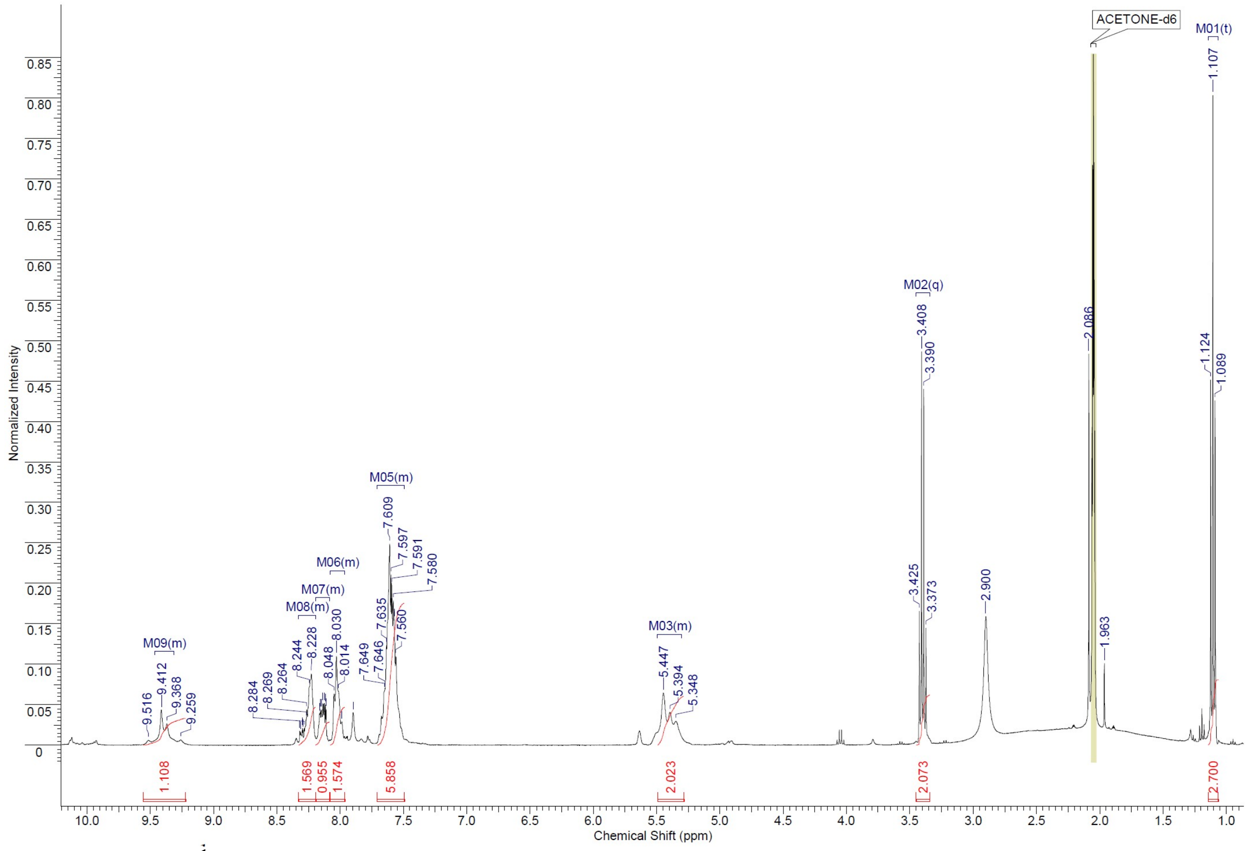

Figure S15. ^{1}H NMR spectra of **20** in acetone-D_6. Signals at δ = 1.02 ppm and δ = 3.39 ppm belong to the protons of EtOAc which forms a stable 1:1 complex with this compound.

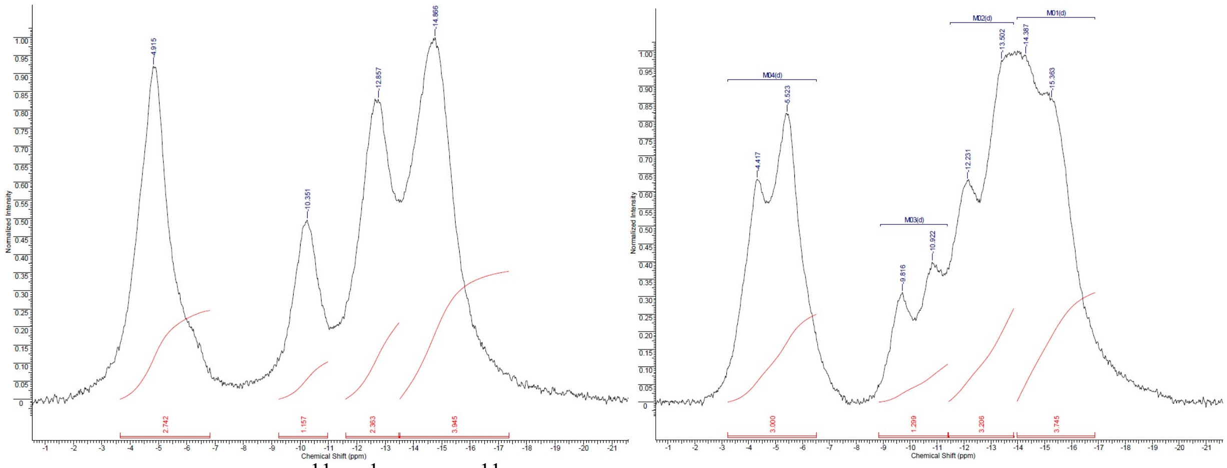

Figure S16. $^{11}B\{^{1}H\}$ and ^{11}B NMR spectra of compound **20** in acetone-D_6.

NMR spectra of compounds **15**-**17**.

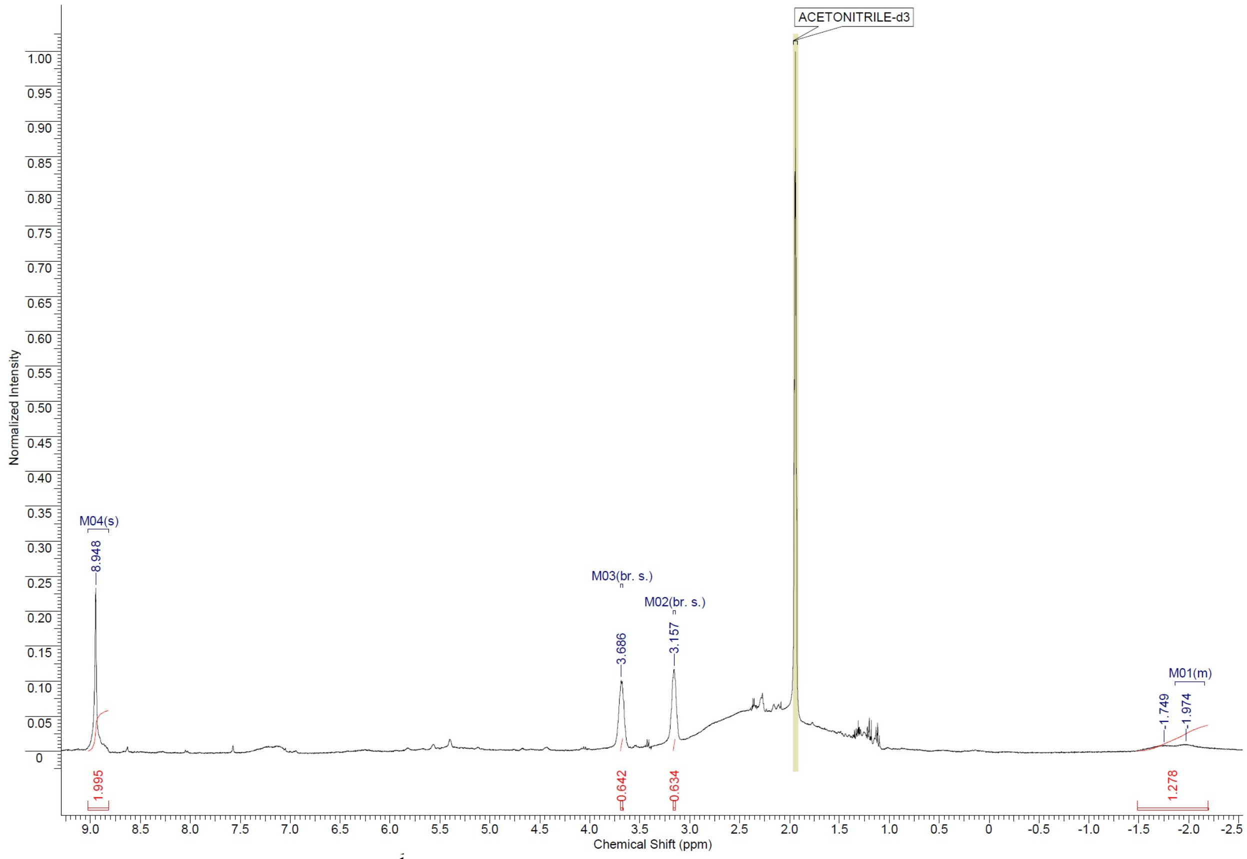

Figure S17. ^{1}H NMR spectra of compound **15** in acetone-D_6.

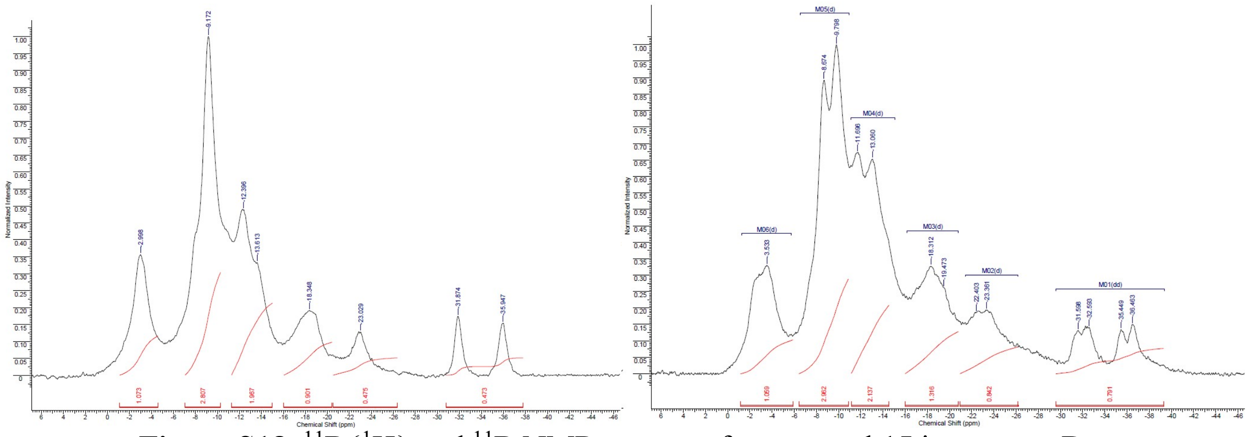

Figure S18. $^{11}B\{^{1}H\}$ and ^{11}B NMR spectra of compound **15** in acetone-D_6.

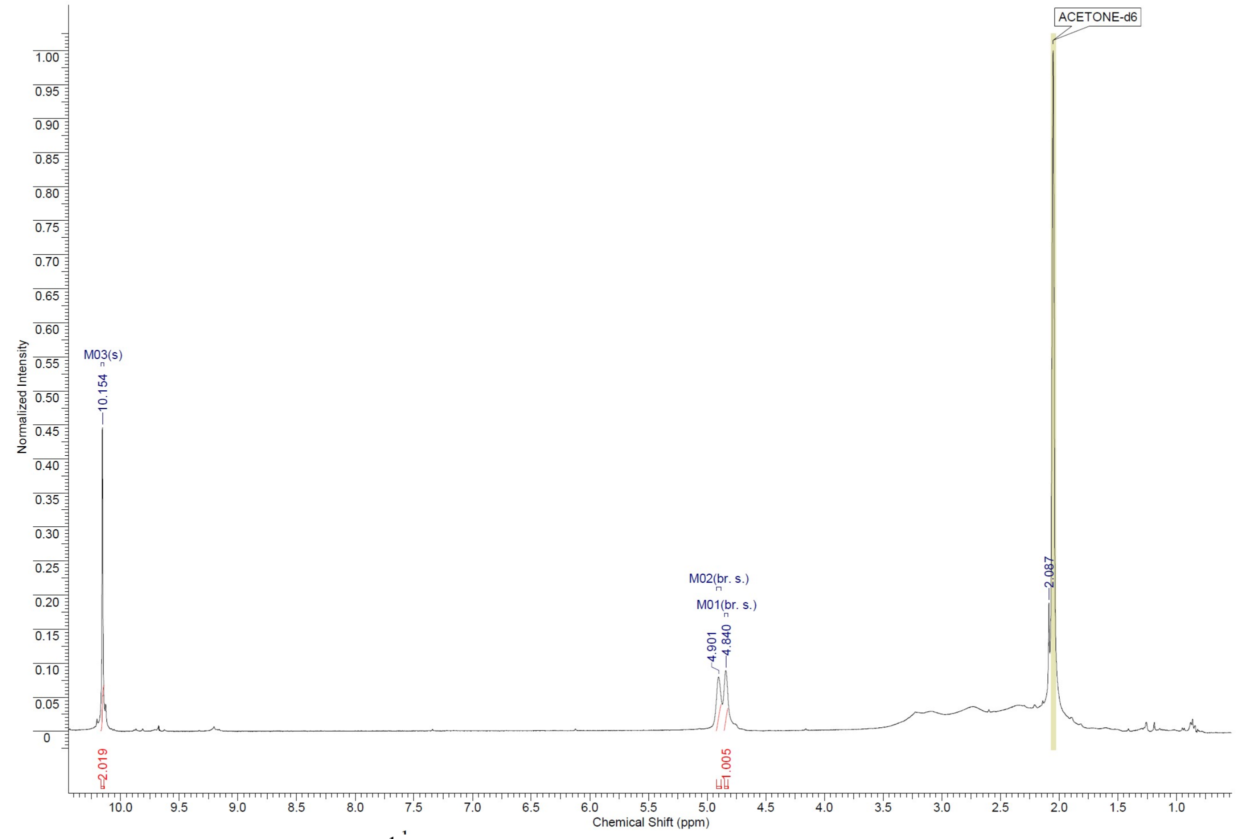

Figure S19. ^{1}H NMR spectra of compound **16** in acetone-D$_6$.

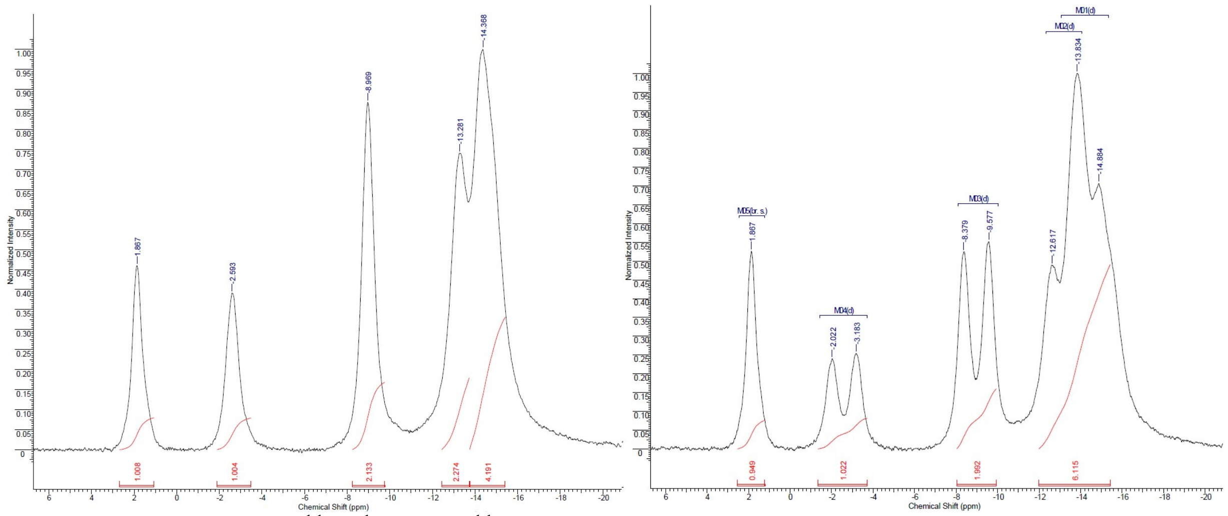

Figure S20. ^{11}B{^{1}H} and ^{11}B NMR spectra of compound **16** in acetone-D$_6$.

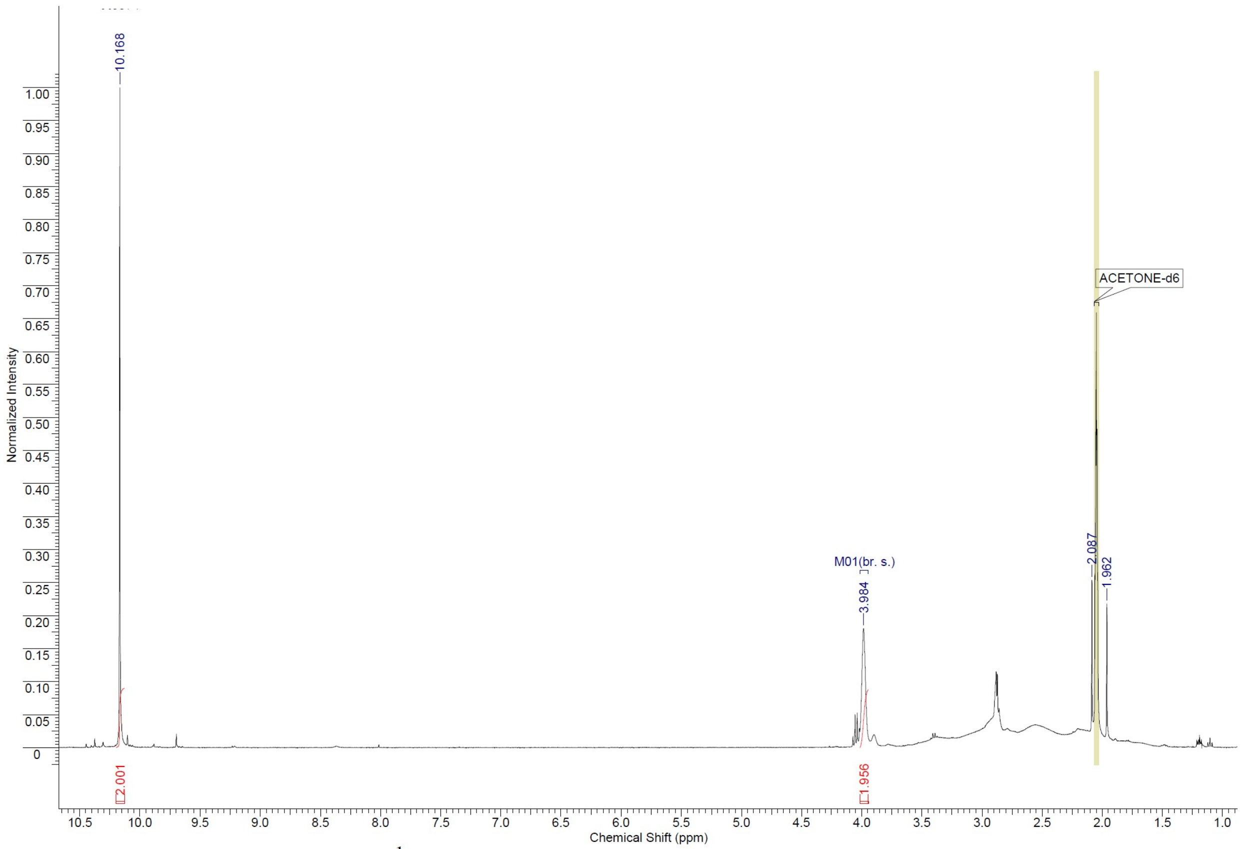

Figure S21. ^{1}H NMR spectra of compound **17** in acetone-D6.

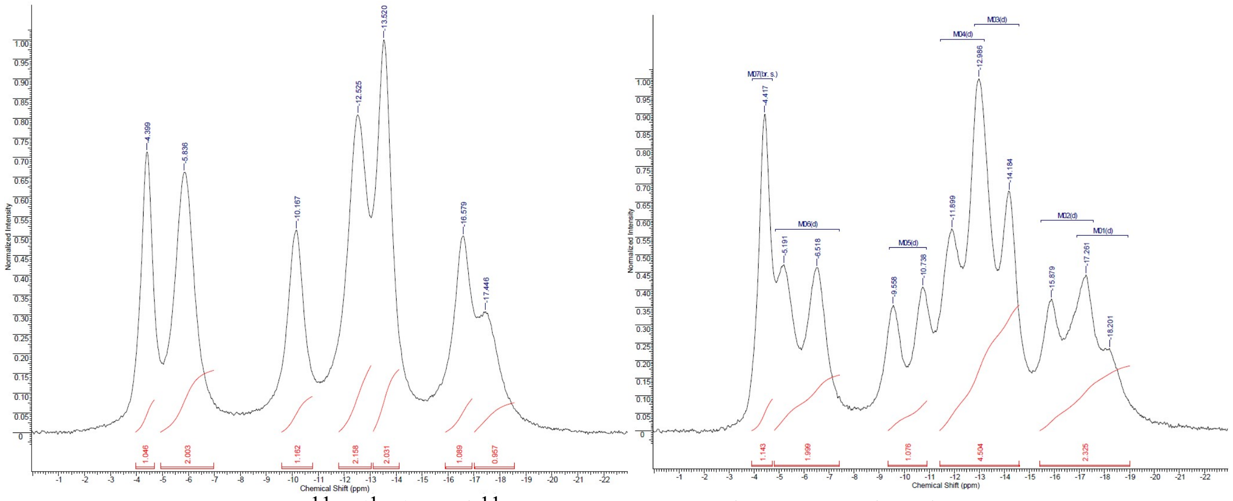

Figure S22. ^{11}B{^{1}H} and ^{11}B NMR spectra of compound **17** in acetone-D6.

X-ray crystallography

Table S1. X-ray data for compound **9**.

B(3)-N(1)	1.4717(15)	N(1)-C(13)-N(2)	119.41(10)
C(13)-N(1)	1.3315(14)	N(1)-C(13)-N(4)	116.99(10)
C(13)-N(2)	1.3625(14)	N(2)-C(13)-N(4)	123.59(10)
C(13)-N(4)	1.3646(14)	N(2)-C(14)-N(3)	129.17(10)
C(14)-N(2)	1.3142(14)	N(2)-C(14)-N(5)	117.16(10)
C(14)-N(3)	1.3372(14)	N(3)-C(14)-N(5)	113.65(10)
C(14)-N(5)	1.4143(14)	N(4)-C(15)-N(3)	129.47(10)
C(15)-N(4)	1.3119(14)	N(4)-C(15)-N(9)	115.03(10)
C(15)-N(3)	1.3299(14)	N(3)-C(15)-N(9)	115.48(10)
C(15)-N(9)	1.4214(14)	N(8)-C(16)-N(5)	108.39(11)
C(16)-N(8)	1.3117(16)	C(13)-N(1)-B(3)	129.41(10)
C(16)-N(5)	1.3549(15)	C(14)-N(2)-C(13)	113.57(10)
C(16)-H(16)	0.930(15)	C(15)-N(3)-C(14)	110.54(10)
N(1)-H(1N)	0.867(16)	C(15)-N(4)-C(13)	113.54(9)
N(5)-N(6)	1.3622(13)	C(16)-N(5)-N(6)	108.69(9)
N(6)-N(7)	1.2890(14)	C(16)-N(5)-C(14)	128.67(10)
N(7)-N(8)	1.3815(15)	N(6)-N(5)-C(14)	122.64(9)
N(9)-C(17)/N(10')	1.3531(14)	N(7)-N(6)-N(5)	105.82(9)
N(9)-N(10)/C(17')	1.3531(14)	N(6)-N(7)-N(8)	111.32(9)
N(11)-N(10)/C(17')	1.2997(15)	C(16)-N(8)-N(7)	105.79(10)
N(12)-C(17)/N(10')	1.2974(14)	C(17)-N(9)-N(10)	108.45(9)
N(11)-N(12)	1.3777(15)	N(10')-N(9)-C(17')	108.45(9)
C(17)-H(17B)	0.9500	N(10')-N(9)-C(15)	124.71(10)
C(17')-H(17A)	0.9500	C(17)-N(9)-C(15)	124.71(10)
		N(10)-N(9)-C(15)	126.75(10)
		C(17')-N(9)-C(15)	126.75(10)
		N(11)-N(10)-N(9)	107.51(10)
		N(11)-C(17')-N(9)	107.51(10)
		C(17')-N(11)-N(12)	108.12(10)
		N(10)-N(11)-N(12)	108.12(10)
		N(10')-N(12)-N(11)	108.60(10)
		C(17)-N(12)-N(11)	108.60(10)
		N(12)-C(17)-N(9)	107.32(10)
		N(12)-N(10')-N(9)	107.32(10)

Table S2. X-ray data for compound **10.**

C(1)-C(2)	1.6448(18)	N(5)-C(14)	1.4179(16)
B(3)-N(1)	1.4742(17)	N(6)-N(7)	1.2838(15)
N(1)-C(13)	1.3359(16)	N(7)-N(8)	1.3768(16)
N(2)-C(14)	1.3129(16)	N(8)-C(16)	1.3123(17)
N(2)-C(13)	1.3547(16)	N(9)-C(17)	1.3642(16)
N(3)-C(14)	1.3283(16)	N(9)-N(10)	1.3765(14)
N(3)-C(15)	1.3308(16)	N(9)-C(15)	1.4156(15)
N(4)-C(15)	1.3151(16)	N(10)-N(11)	1.2810(15)
N(4)-C(13)	1.3619(15)	N(11)-N(12)	1.3798(16)
N(5)-C(16)	1.3659(16)	N(12)-C(17)	1.3120(17)
N(5)-N(6)	1.3722(14)		

Table S3. X-ray data for compound **19.**

N(1)-C(3)	1.338(3)	N(4)-C(3)	1.315(3)
N(1)-B(3)	1.471(4)	N(4)-C(5)	1.360(3)
N(1)-H(1N)	0.80(4)	N(5)-C(6)	1.328(4)
N(2)-C(4)	1.361(3)	N(6)-C(4)	1.305(3)
N(2)-N(5)	1.377(3)	N(6)-C(6)	1.388(4)
N(2)-C(3)	1.397(3)	N(7)-C(5)	1.320(3)
N(3)-C(4)	1.380(3)	N(7)-N(8)	1.397(3)
N(3)-C(13)	1.396(3)	N(8)-C(13)	1.315(4)
N(3)-C(5)	1.397(3)		
C(3)-N(1)-B(3)	126.3(2)	N(4)-C(3)-N(1)	123.4(2)
C(3)-N(1)-H(1N)	116(3)	N(4)-C(3)-N(2)	119.4(2)
B(3)-N(1)-H(1N)	117(3)	N(1)-C(3)-N(2)	117.2(2)
C(4)-N(2)-N(5)	108.7(2)	N(6)-C(4)-N(2)	112.2(2)
C(4)-N(2)-C(3)	124.5(2)	N(6)-C(4)-N(3)	132.0(2)
N(5)-N(2)-C(3)	126.6(2)	N(2)-C(4)-N(3)	115.7(2)
C(4)-N(3)-C(13)	135.2(2)	N(7)-C(5)-N(4)	126.2(2)
C(4)-N(3)-C(5)	118.3(2)	N(7)-C(5)-N(3)	109.9(2)
C(13)-N(3)-C(5)	105.6(2)	N(4)-C(5)-N(3)	123.9(2)
C(3)-N(4)-C(5)	117.7(2)	N(5)-C(6)-N(6)	115.6(2)
C(6)-N(5)-N(2)	101.9(2)	N(5)-C(6)-C(7)	123.4(2)
C(4)-N(6)-C(6)	101.5(2)	N(6)-C(6)-C(7)	120.9(2)
C(5)-N(7)-N(8)	106.5(2)	N(8)-C(13)-N(3)	107.9(2)
C(13)-N(8)-N(7)	110.1(2)	N(8)-C(13)-C(14)	126.0(2)
		N(3)-C(13)-C(14)	125.9(2)

Table S4. X-ray data for compound **20.**

O(1A)-C(6A)	1.221(4)	O(1B)-C(6B)	1.235(5)
N(1A)-C(3A)	1.349(5)	N(1B)-C(3B)	1.359(5)
N(1A)-B(3A)	1.470(6)	N(1B)-B(3B)	1.457(6)
N(2A)-C(4A)	1.340(4)	N(2B)-C(4B)	1.341(5)
N(2A)-C(3A)	1.345(5)	N(2B)-C(3B)	1.343(5)
N(3A)-C(5A)	1.327(5)	N(3B)-C(5B)	1.302(5)
N(3A)-C(4A)	1.358(5)	N(3B)-C(4B)	1.355(5)
N(4A)-C(5A)	1.309(5)	N(4B)-C(5B)	1.318(5)
N(4A)-C(3A)	1.353(5)	N(4B)-C(3B)	1.355(5)
N(5A)-C(4A)	1.349(5)	N(5B)-C(4B)	1.356(5)
N(5A)-N(6A)	1.381(4)	N(5B)-N(6B)	1.372(4)
N(6A)-C(6A)	1.364(5)	N(6B)-C(6B)	1.359(5)
N(7A)-N(9A)	1.339(4)	N(7B)-N(8B)	1.332(4)
N(7A)-N(8A)	1.339(4)	N(7B)-N(9B)	1.341(4)
N(7A)-C(5A)	1.442(5)	N(7B)-C(5B)	1.447(5)
N(8A)-C(13A)	1.331(5)	N(8B)-C(13B)	1.327(5)
N(9A)-N(10A)	1.313(4)	N(9B)-N(10B)	1.313(5)
N(10A)-C(13A)	1.377(5)	N(10B)-C(13B)	1.364(5)
C(3A)-N(1A)-B(3A)	128.3(4)	C(3B)-N(1B)-B(3B)	129.5(3)

C(4A)-N(2A)-C(3A)	114.1(3)	C(4B)-N(2B)-C(3B)	113.1(3)
C(5A)-N(3A)-C(4A)	110.6(3)	C(5B)-N(3B)-C(4B)	110.7(3)
C(5A)-N(4A)-C(3A)	112.9(3)	C(5B)-N(4B)-C(3B)	111.5(3)
C(4A)-N(5A)-N(6A)	119.4(3)	C(4B)-N(5B)-N(6B)	119.9(3)
C(6A)-N(6A)-N(5A)	122.0(3)	C(6B)-N(6B)-N(5B)	120.0(3)
N(9A)-N(7A)-N(8A)	114.6(3)	N(8B)-N(7B)-N(9B)	113.6(3)
N(9A)-N(7A)-C(5A)	123.6(3)	N(8B)-N(7B)-C(5B)	123.1(3)
N(8A)-N(7A)-C(5A)	121.8(3)	N(9B)-N(7B)-C(5B)	123.3(3)
C(13A)-N(8A)-N(7A)	101.2(3)	C(13B)-N(8B)-N(7B)	102.0(3)
N(10A)-N(9A)-N(7A)	105.5(3)	N(10B)-N(9B)-N(7B)	105.7(3)
N(9A)-N(10A)-C(13A)	106.6(3)	N(9B)-N(10B)-C(13B)	106.6(3)
N(2A)-C(3A)-N(1A)	118.2(3)	N(2B)-C(3B)-N(4B)	125.9(4)
N(2A)-C(3A)-N(4A)	124.9(3)	N(2B)-C(3B)-N(1B)	117.3(3)
N(1A)-C(3A)-N(4A)	116.9(4)	N(4B)-C(3B)-N(1B)	116.8(3)
N(2A)-C(4A)-N(5A)	117.5(3)	N(2B)-C(4B)-N(3B)	127.2(4)
N(2A)-C(4A)-N(3A)	126.8(3)	N(2B)-C(4B)-N(5B)	118.2(4)
N(5A)-C(4A)-N(3A)	115.6(3)	N(3B)-C(4B)-N(5B)	114.6(3)
N(4A)-C(5A)-N(3A)	130.6(4)	N(3B)-C(5B)-N(4B)	131.4(4)
N(4A)-C(5A)-N(7A)	113.0(3)	N(3B)-C(5B)-N(7B)	114.9(3)
N(3A)-C(5A)-N(7A)	116.4(3)	N(4B)-C(5B)-N(7B)	113.7(3)
O(1A)-C(6A)-N(6A)	120.8(4)	O(1B)-C(6B)-N(6B)	121.5(4)
O(1A)-C(6A)-C(7A)	123.8(4)	O(1B)-C(6B)-C(7B)	122.5(4)
N(6A)-C(6A)-C(7A)	115.4(3)	N(6B)-C(6B)-C(7B)	115.9(4)
N(8A)-C(13A)-N(10A)	112.2(3)	N(8B)-C(13B)-N(10B)	112.0(4)
N(8A)-C(13A)-C(14A)	123.6(4)	N(8B)-C(13B)-C(14B)	124.1(4)
N(10A)-C(13A)-C(14A)	124.2(3)	N(10B)-C(13B)-C(14B)	123.9(4)

Table S5. Crystal Data, Data Collection and Structure Refinement Parameters for **9, 10, 19**, and **20**.

Identification code	**9**	**10**	**19**	**20**
CCDC No	2177931	2177932	2177933	2177934
Empirical formula	$C_7H_{14}B_{10}N_{12}$ $(CH_3)_2CO$	$C_9H_{18}B_{10}N_{12}$ $(CH_3)_2CO$	$C_{19}H_{22}B_{10}N_8$ $2(CH_3OH)$	$C_{19}H_{24}B_{10}N_{10}O$, CH_2Cl_2
Molecular weight	432.48	460.53	534.63	601.51
Crystal size (mm)	0.42×0.36×0.06	0.50×0.50×0.10	0.55×0.10×0.05	0.35×0.25×0.05
Temperature (K)	120(2)	100(2)	120(2)	120(2)
Crystal system	triclinic	monoclinic	orthorhombic	triclinic
Space group	*P*-1	$P2_1$/c	*A*ba2	*P*-1
a (Å)	6.6639(2)	14.7936(5)	26.624(2)	14.089(2)
b (Å)	10.5197(4)	11.9606(4)	28.969(2)	15.442(3)
c (Å)	16.0485(5)	13.7459(5)	7.2719(6)	16.375(3)
α (deg)	108.8447(7)	90	90	109.645(4)
β (deg)	92.4433(8)	111.6708(12)	90	95.426(4)
γ (deg)	98.1904(8)	90	90	116.641(4)
V (Å^3)	1049.14(6)	2260.30(14)	5608.4(8)	2868.8(9)
Z	2	4	8	4
d_{calcd} (g·cm^{-3})	1.369	1.353	1.266	1.393
linear absorption μ (cm^{-1})	0.088	0.086	0.078	0.264

$2\theta_{max}$ (deg)	60	56	56	52
Reflections collected	23672	23119	38136	28302
Independent reflections (R_{int})	6138 (0.0379)	5447 (0.0322)	6771 (0.0630)	11263 (0.1204)
Observed reflections ($I>2\sigma(I)$)	4609	4609	5735	5302
Number of parameters	392	368	428	721
R_1 (on F for $I>2\sigma(I)$)[a]	0.0426	0.0419	0.0457	0.0720
wR_2 (on F^2 for all data)[b]	0.1184	0.1038	0.1046	0.2002
GOOF	1.010	1.047	1.075	0.984
Largest diff. peak/hole (e Å^{-3})	0.344/-0.227	0.275/-0.218	0.247/-0.205	0.270/-0.288

[a] $R_1 = \Sigma||F_o| - |F_c||/\Sigma|F_o|$

[b] $wR_2 = \{\Sigma[w(F_o^2 - F_c^2)^2]/\Sigma w(F_o^2)^2\}^{1/2}$

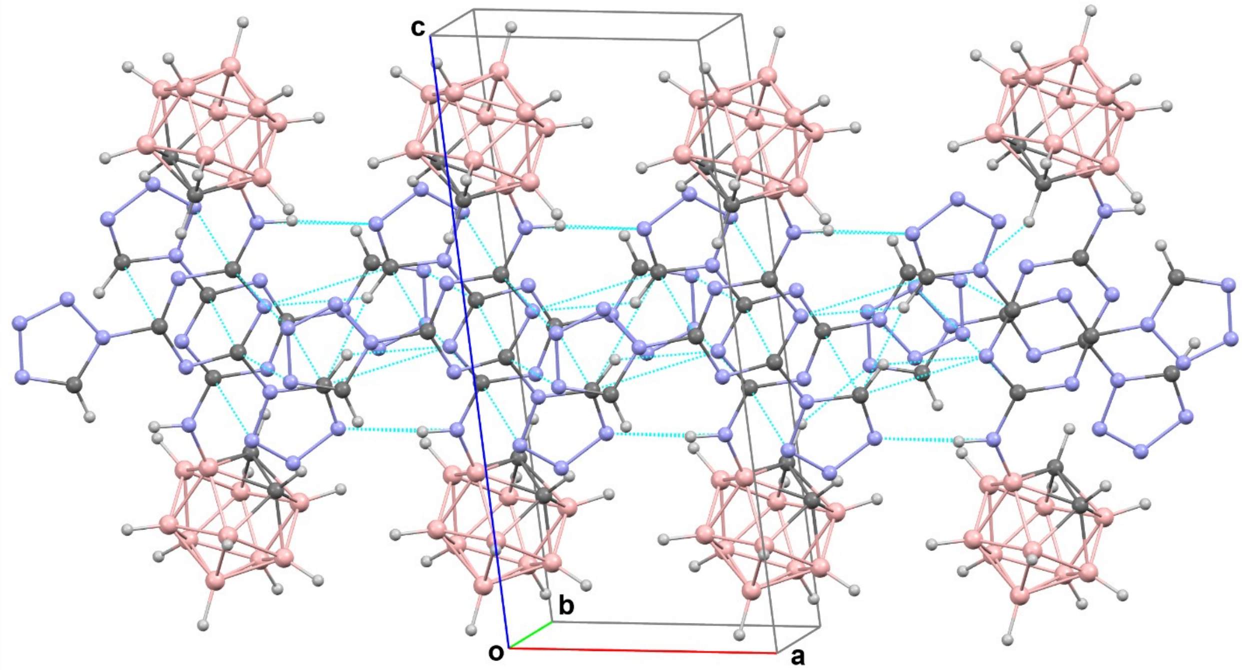

Figure S23. The fragment of double layer in the crystal of **9** (projection along *b* axis). The shortened intermolecular contacts are shown with dotted lines.

Table S6. Hydrogen bonds observed in the crystal of compound **9**.

D-H···A	d(D-H), Å	d(H···A), Å	d(D···A), Å	<(DHA), deg.
N(1)-H(1N)···N(12A)#1	0.868(15)	2.146(15)	3.0101(15)	173.9(15)
C(17)-H(17B)···N(10')#1	0.95	2.44	3.3789(15)	170
C(17)-H(17B)···N(4A)#1	0.95	2.60	3.2285(15)	124
C(16)-H(16)···O(1S)	0.929(15)	2.099(16)	2.982(4)	158.3(13)
C(16)-H(16)···O(1')	0.929(15)	2.403(16)	3.316(3)	167.3(14)
C(17')-H(17A)···O(1')	0.95	2.30	3.245(4)	178
C(1)-H(1)…N(8)#2	0.998(15)	2.405(15)	3.2702(17)	144.6(12)

Symmetry transformations used to generate equivalent atoms: #1 -1-x,1-y,1-z; #2. 1-x,2-y,1-z.

Table S7. Hydrogen bonds observed in the crystal of compound **10**.

D-H···A	d(D-H), Å	d(H···A), Å	d(D···A), Å	<(DHA), deg.
N(1)-H(1N)...O(1S)$^{\#1}$	0.929(19)	2.017(19)	2.9363(14)	170.1(16)
C(1)-H(1)…N(12)$^{\#2}$	0.950(17)	2.405(16)	3.3098(17)	159.0(13)
C(2)-H(2)…N(2)	0.990(18)	2.443(17)	3.0093(17)	115.8(12)

Symmetry transformations used to generate equivalent atoms: #1. -x,-y,-z; #2 -x,1/2+y,1/2-z

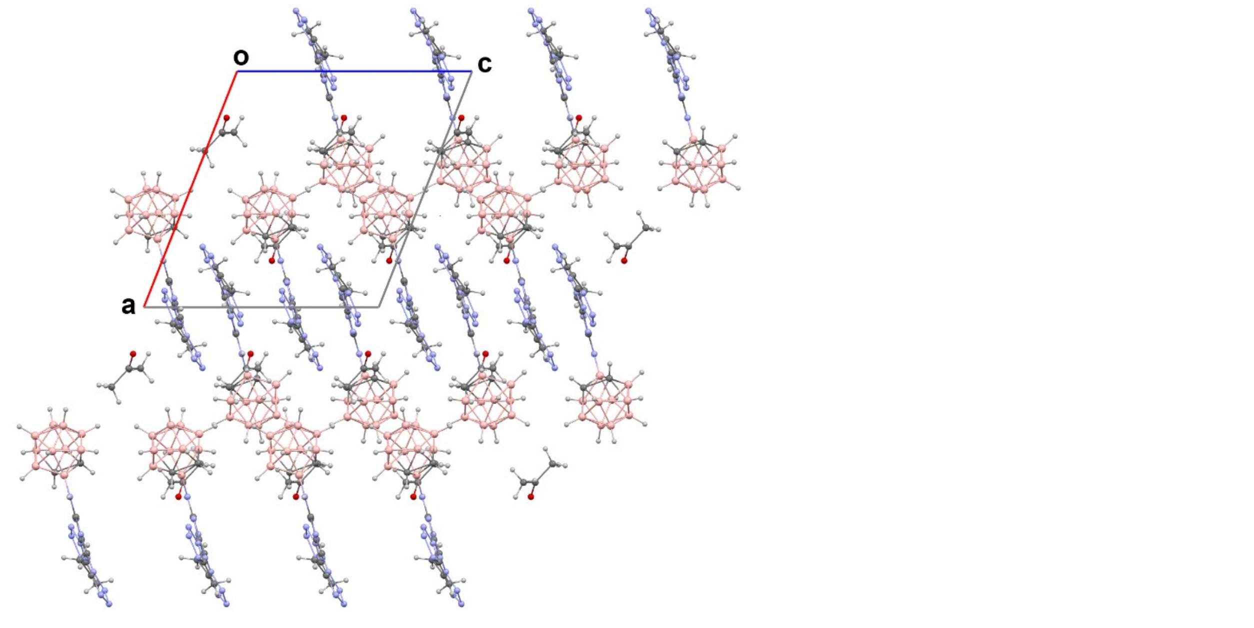

Figures S24. Fragment of crystal packing in **10** (projection along *b* axis).

Table S8. Hydrogen bonds observed in the crystal of compound **19**.

D-H···A	d(D-H), Å	d(H···A), Å	d(D···A), Å	<(DHA), deg.
N(1)-H(1N)···O(1S)$^{\#1}$	0.80(4)	2.12(4)	2.892(3)	162(4)
O(1S)-H(1S)···O(2S)	0.85(5)	1.92(5)	2.760(3)	168(4)
O(2S)-H(2S)···N(8)	0.79(4)	2.05(4)	2.815(3)	161(4)
C(1)-H(1)…N(7)$^{\#2}$	0.95(3)	2.83(3)	3.472(4)	126(3)
C(2)-H(2)…N(7)$^{\#2}$	0.95(4)	2.65(4)	3.368(4)	133(3)
C(2)-H(2)…O(2S)$^{\#2}$	0.95(4)	2.42(4)	3.253(4)	146(3)

Symmetry transformations used to generate equivalent atoms: #1. 1/2-x,y,-1/2+z; #2. 1/2-x,y,1/2+z.

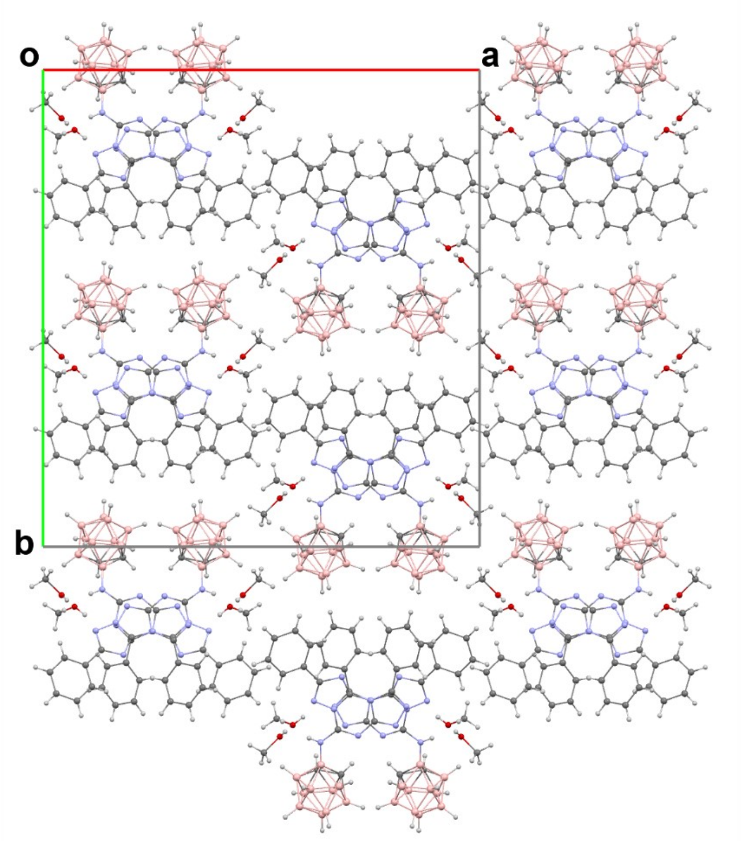

Figure S25. Fragment of crystal packing in **19** (projection along *c* axis).

Table S9. Hydrogen bonds observed in the crystal of compound **20**.

D-H···A	d(D-H), Å	d(H···A), Å	d(D···A), Å	<(DHA), deg.
N(5A)-H(5AA)···O(1B)	0.88	2.04	2.835(4)	150
N(5B)-H(5BA)···O(1A)	0.88	2.07	2.871(4)	151
N(5B)-H(5BA)···N(6B)[#1]	0.88	2.62	3.281(5)	132
N(6B)-H(6BA)···O(1A)[#1]	0.88	2.19	2.891(6)	137
C(1A)-H(1A)…N(3A)[#2]	0.98	2.46	3.424(6)	167
C(1A)-H(1A)…N(9A)[#2]	0.98	2.64	3.319(6)	126
C(2B)-H(2B)…N(10A)	0.98	2.45	3.373(6)	157

Symmetry transformations used to generate equivalent atoms: #1. 1-x,2-y,1-z; #2. 1-x,1-y,1-z.

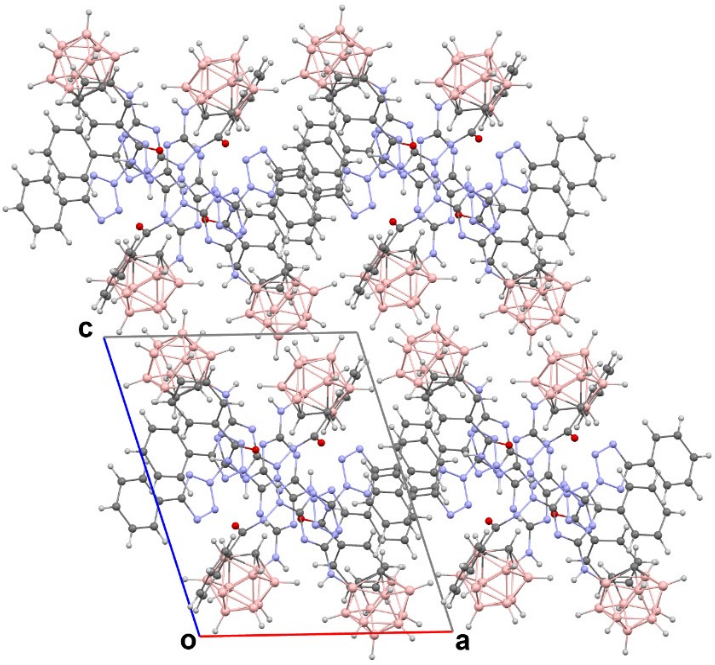

Figure S26. Fragment of crystal packing of **20** (projection along b axis).

Hirshfeld surfaces analysis.

Hirshfeld surface for all compounds plotted over d_{norm} property (white - contacts around the van der Waals radii, blue - the longer intermolecular contacts and red - shorter intermolecular contacts of the molecules) using the CrystalExplorer17 software [1].

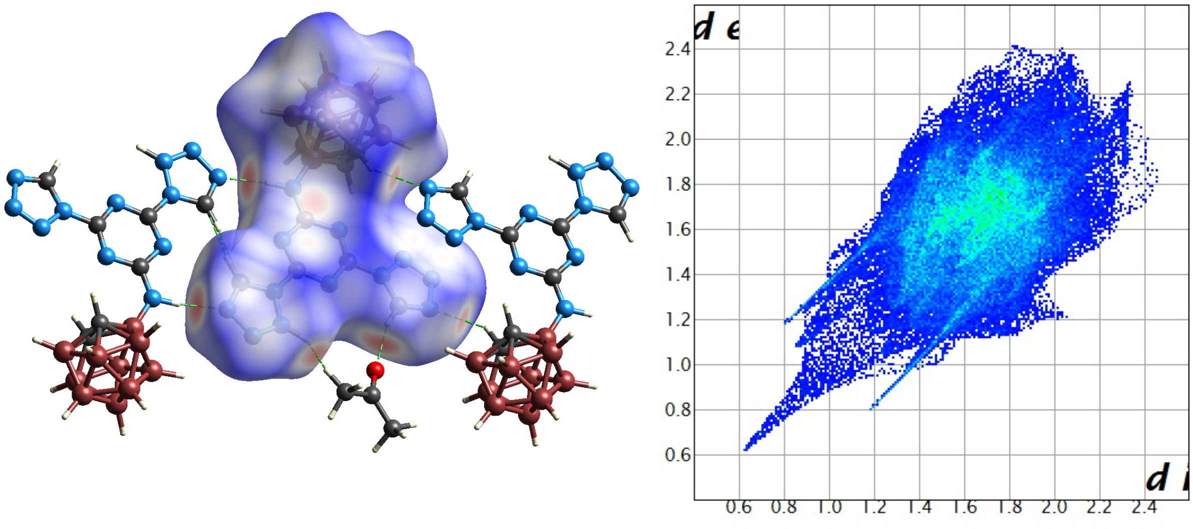

(a) (b)

Figure S27. Hirshfeld surfaces (a) and full fingerprint plots (b) of compound **9.**

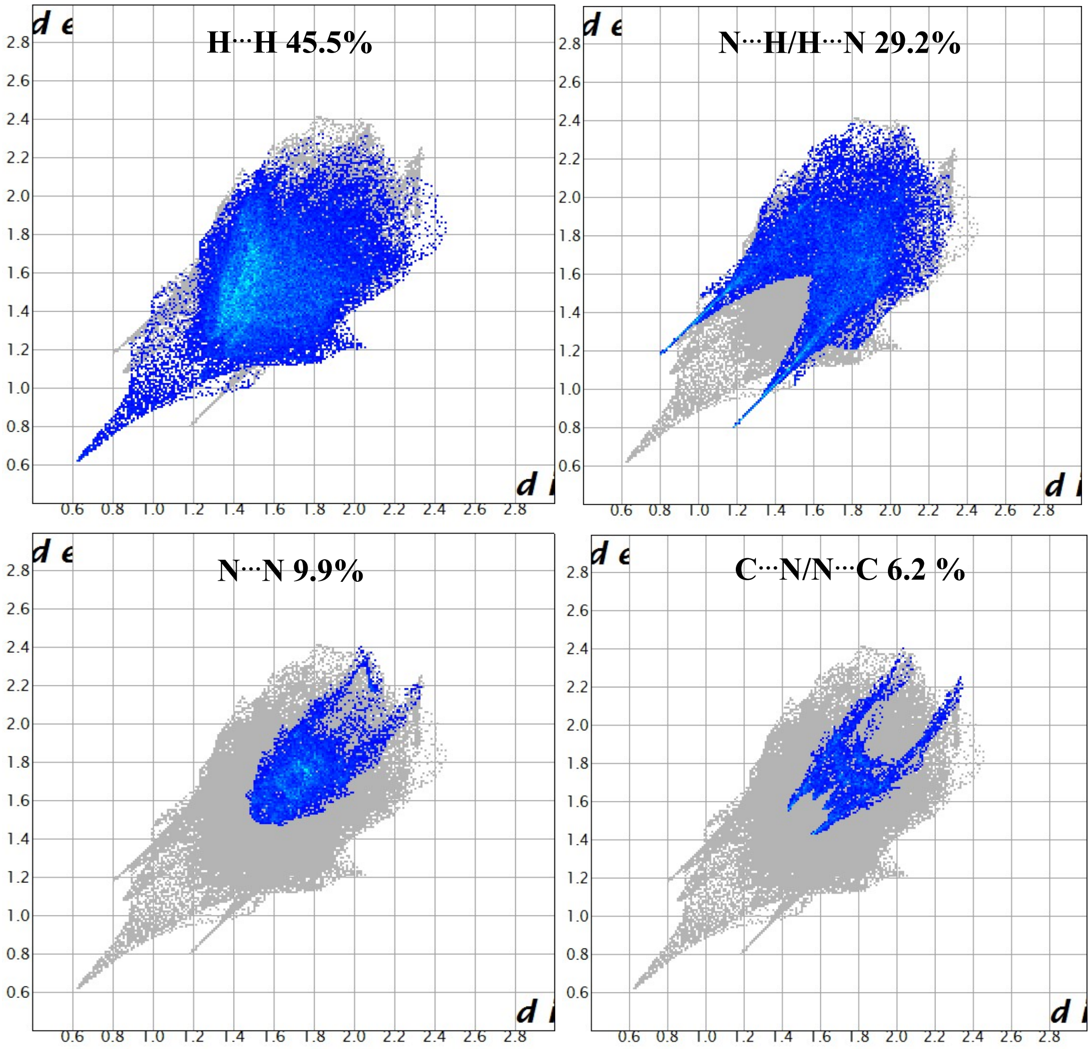

Figure S28. Individual interatomic contacts of compound **9** with percentage contribution in the crystal packing greater than 4.5%. The contribution of H···O/O···H 4.3%, C···H/H···C 2.4%, N···O/O···N 1% interactions.

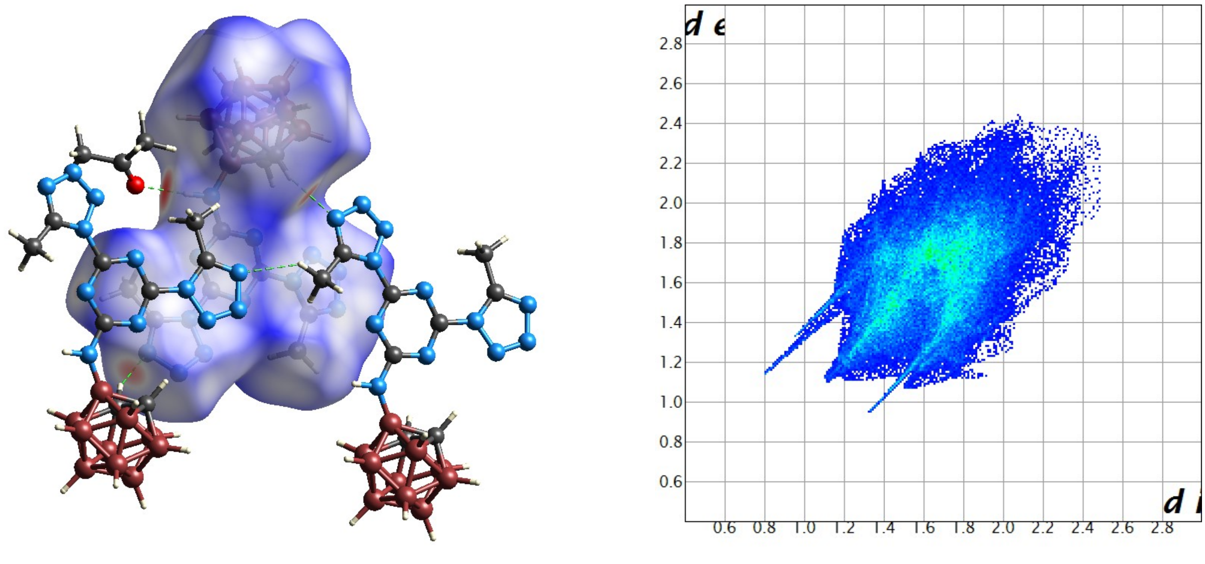

(a) (b)

Figure S29. Hirshfeld surfaces (a) and full fingerprint plots (b) of compound **10.**

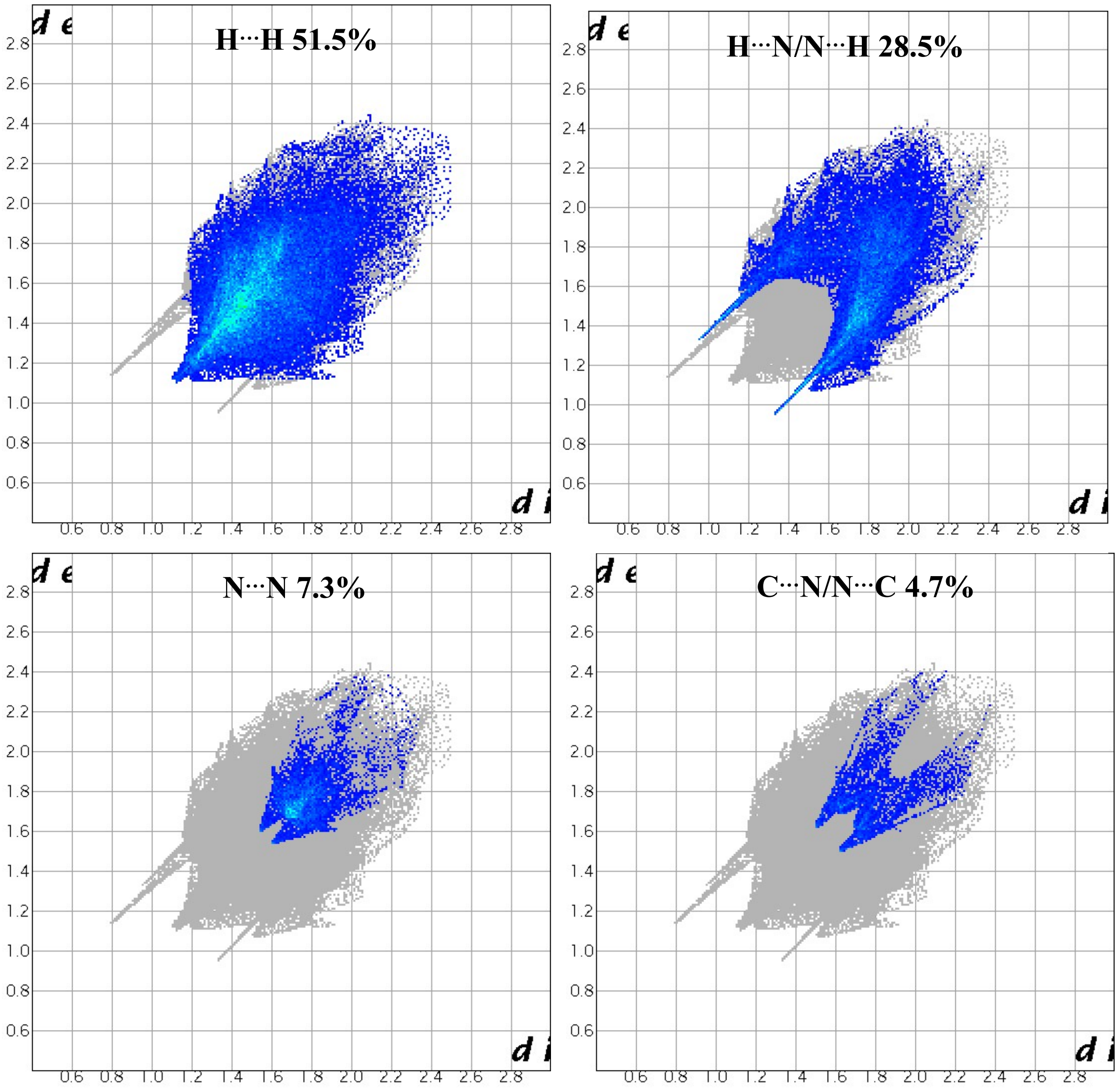

Figure S30. Individual interatomic contacts of compound **10** with percentage contribution in the crystal packing greater than 4.5%. The contribution of C-H+H-C 3.7%, H-O+O-H 3.3%, N-O+O-N 0.4%, C-O+O-C 0.1% interactions.

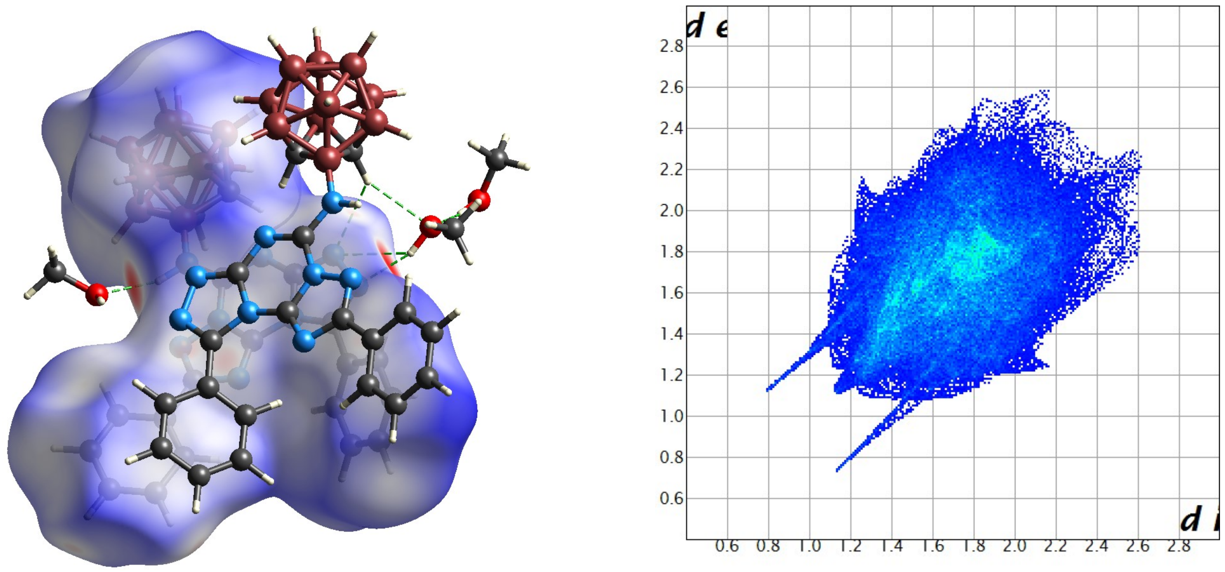

(a) (b)

Figure S31. Hirshfeld surfaces (a) and full fingerprint plots (b) of compound **19.**

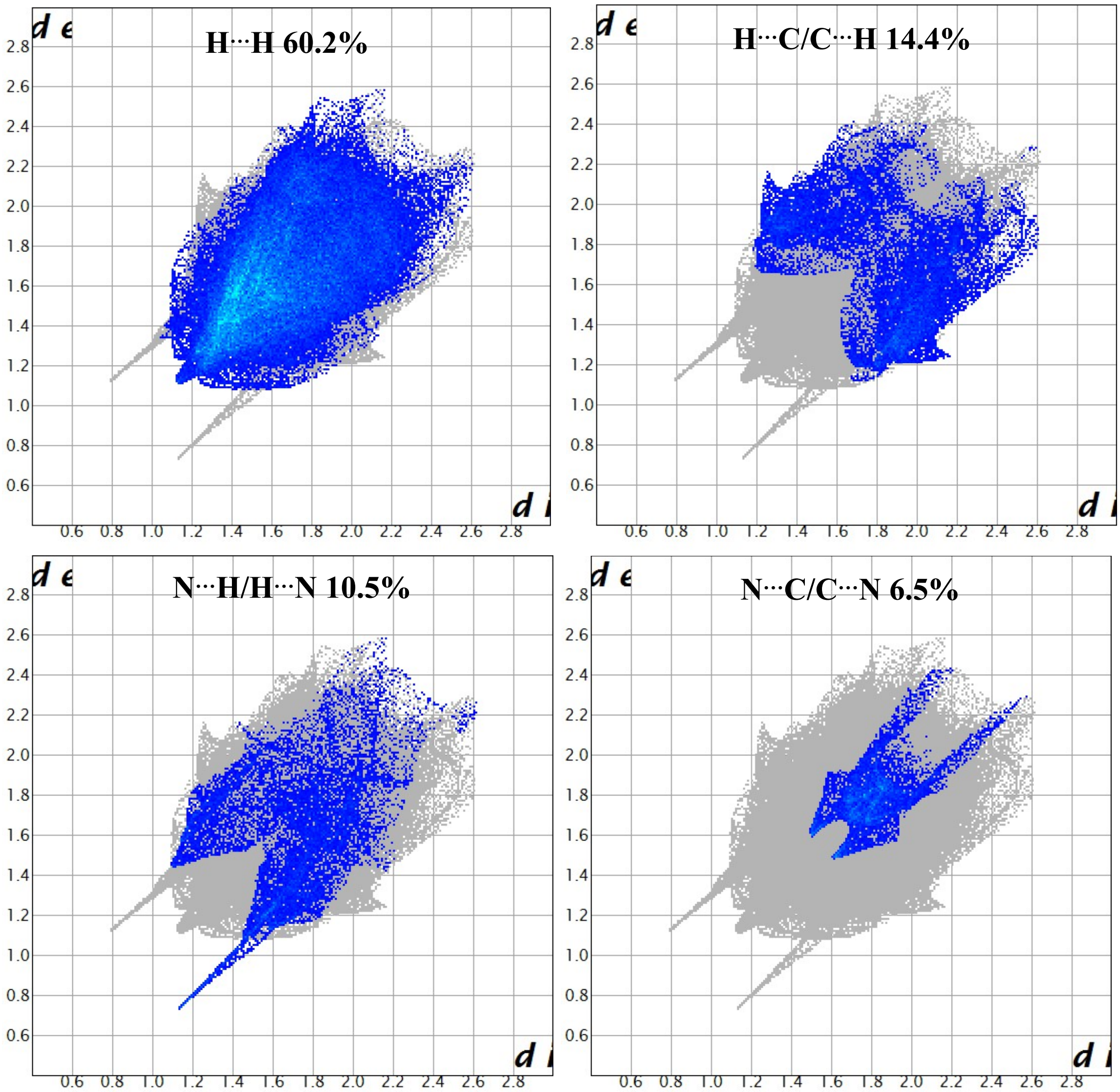

Figure S32. Individual interatomic contacts of compound **19** with percentage contribution in the crystal packing greater than 4.5%. The contribution of C···C 3.5%, H···O/O···H 2.5%, N···N 2.4% interactions.

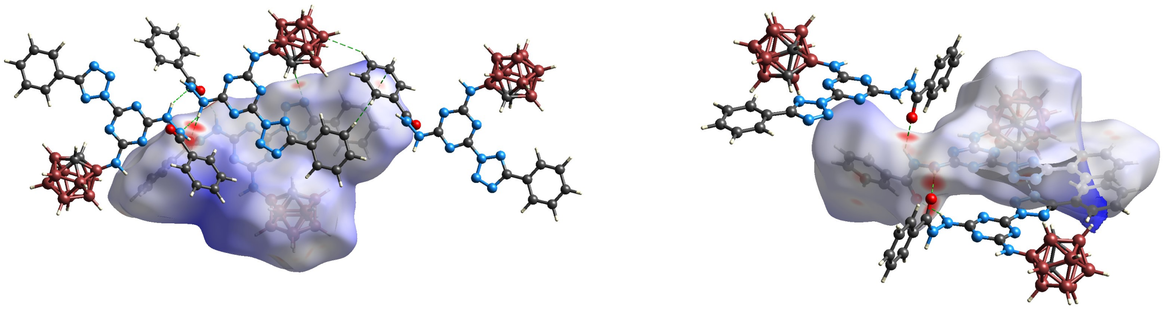

A B

Figure S33. Hirshfeld surfaces of compound **20** (for two crystallographically independent molecules A and B).

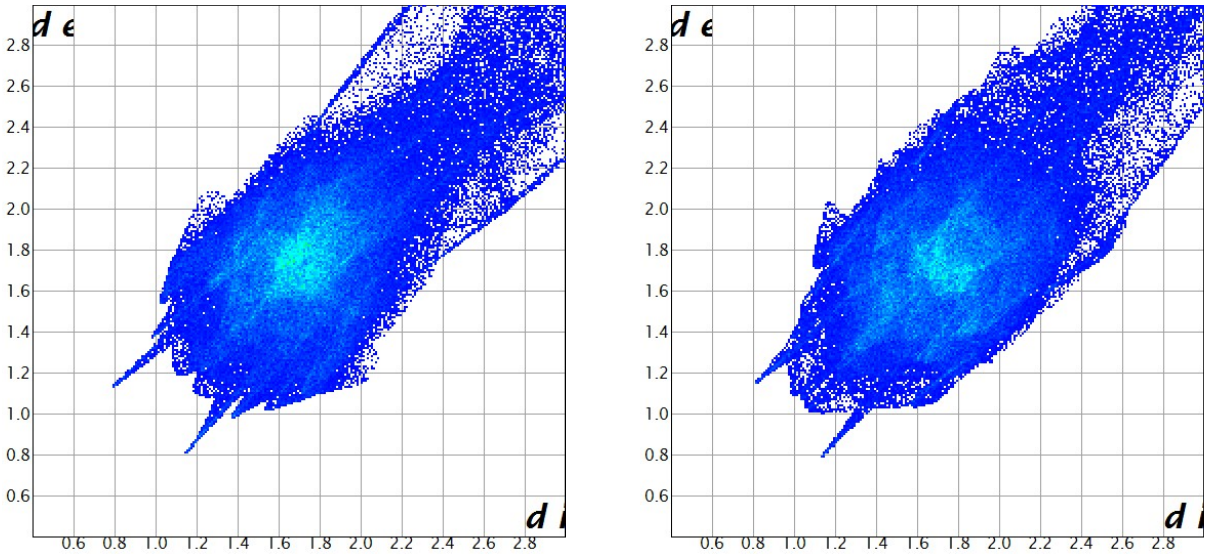

A B

Figure S34. Full fingerprint plots (b) of compound **20** (for two crystallographically independent molecules A and B)

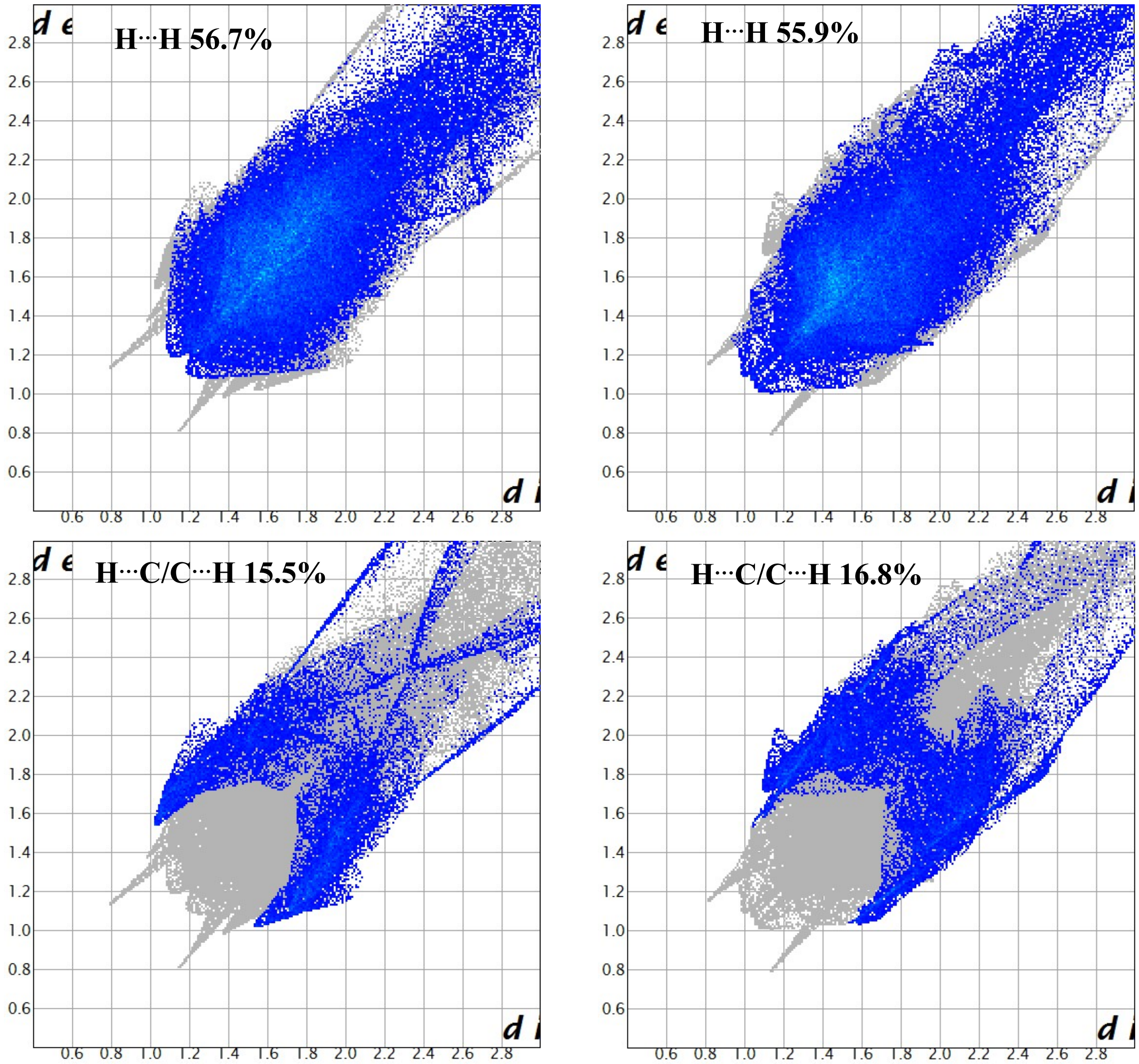

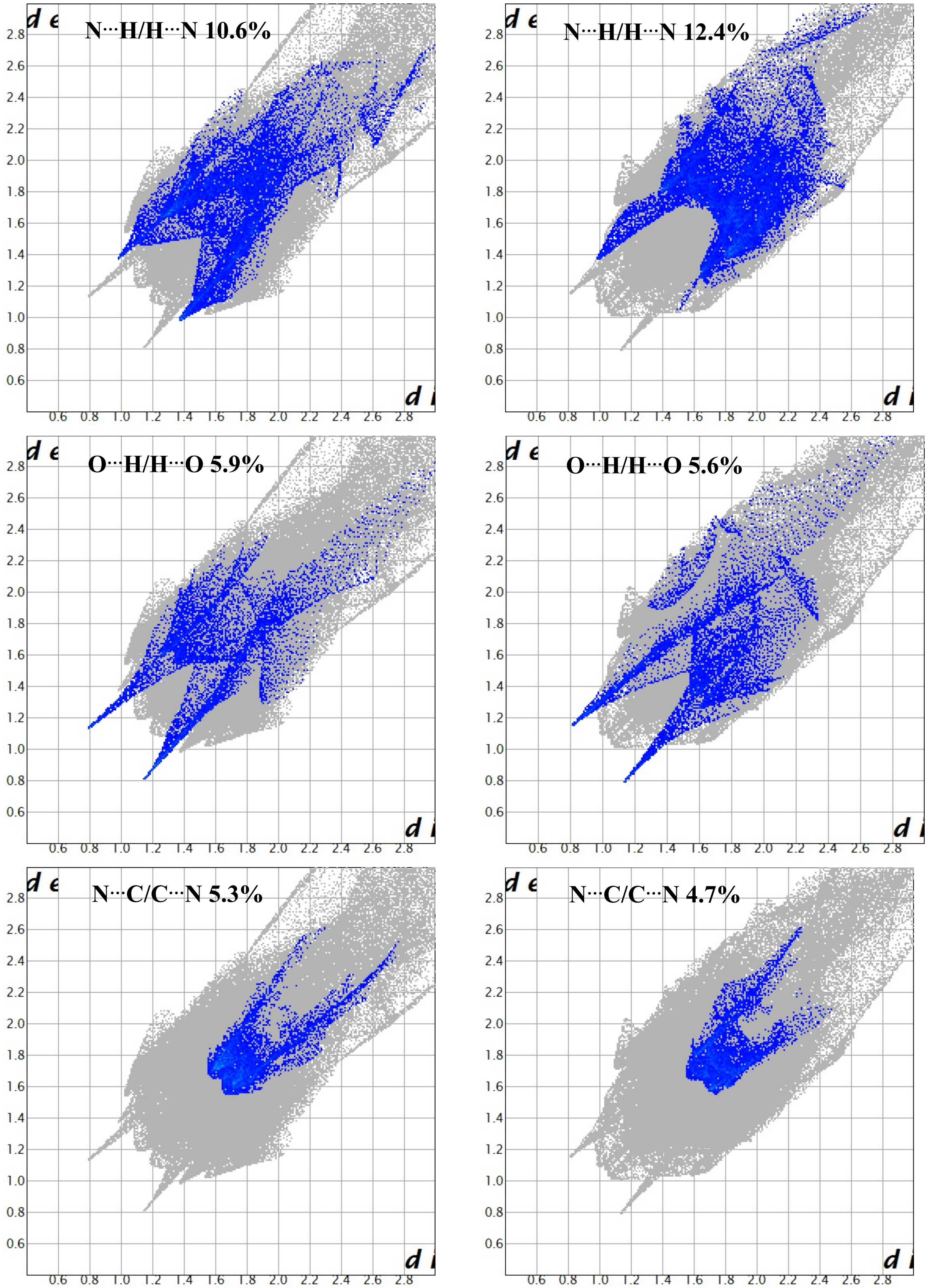

The contribution of O···H/H···O 5.9%, N···N 4.2%, C···C 1.5%, O···N/N···O 0.2% interactions indicated for molecule A.

The contribution of N···N 2.3%, C···C 2.0%, O···N/N···O 0.2% interactions indicated for molecule B.

Figure S35. Individual interatomic contacts of compound **20** (for two crystallographically independent molecules A and B) with percentage contribution in the crystal packing greater than 4.5%.

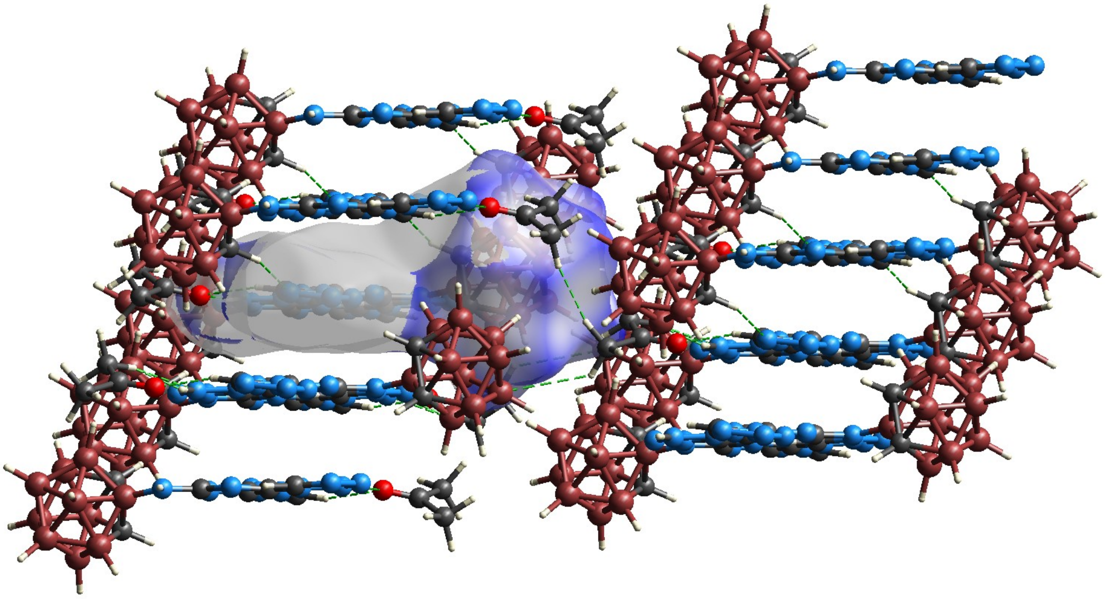

Figure S36. Supramolecular assembling of compound **9**. Hirshfeld surfaces mapped over d_{norm}, generated from intermolecular H···H contacts.

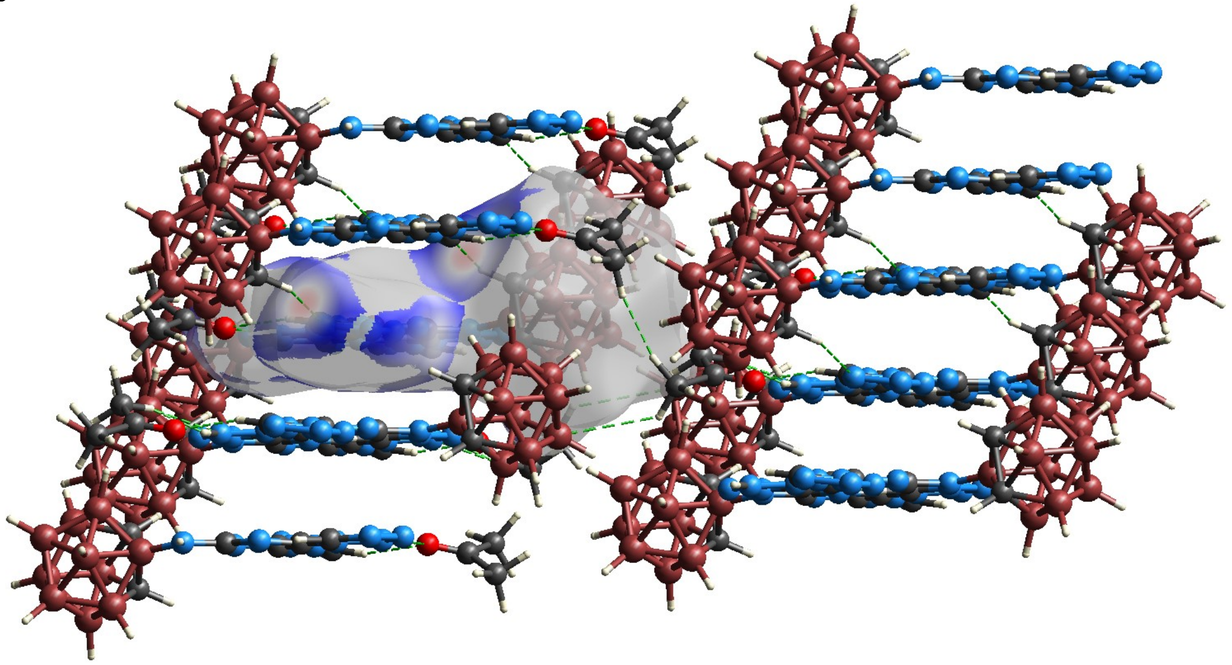

Figure S37. Supramolecular assembling of compound **9**. Hirshfeld surfaces mapped over d_{norm}, generated from intermolecular N···H contacts.

Calculated geometry and electronic structure of compounds 9-11, 15-17, 19 and 25.

Table S10. Geometry structure of studied compounds.

№ of compound	geometry structure
**9**	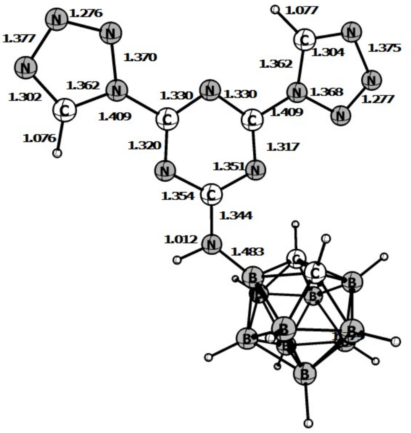
**10**	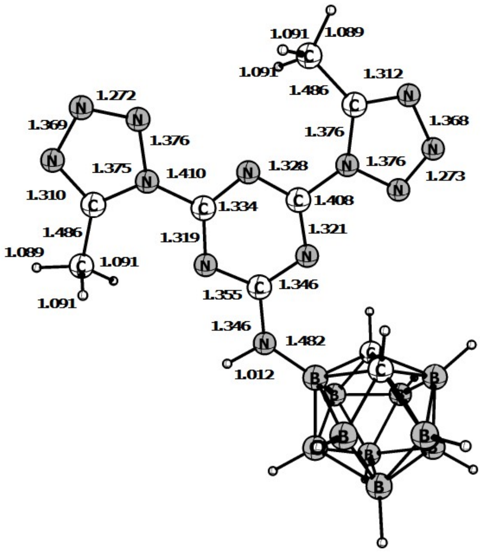
**11**	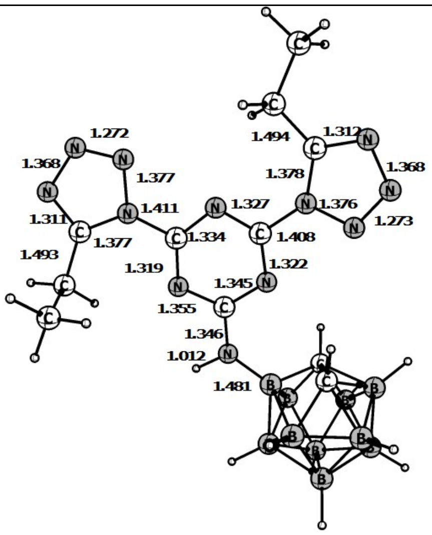

<table>
<tr><td>15</td><td>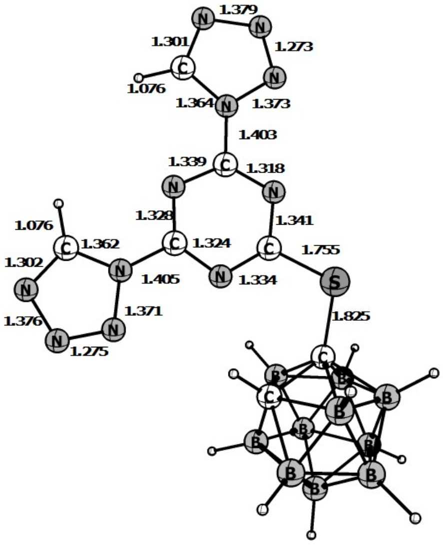
</td></tr>
<tr><td>16</td><td>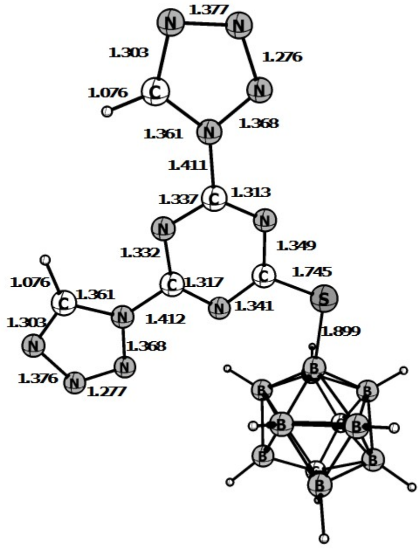
</td></tr>
<tr><td>17</td><td>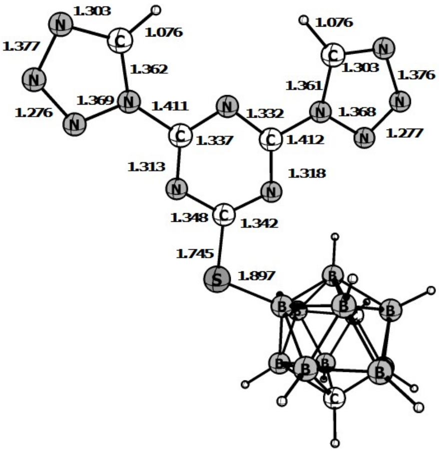
</td></tr>
</table>

<table>
<tr><td>19</td><td>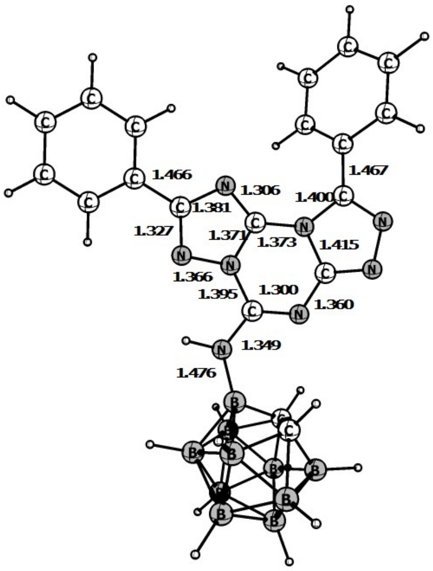
</td></tr>
<tr><td>25</td><td>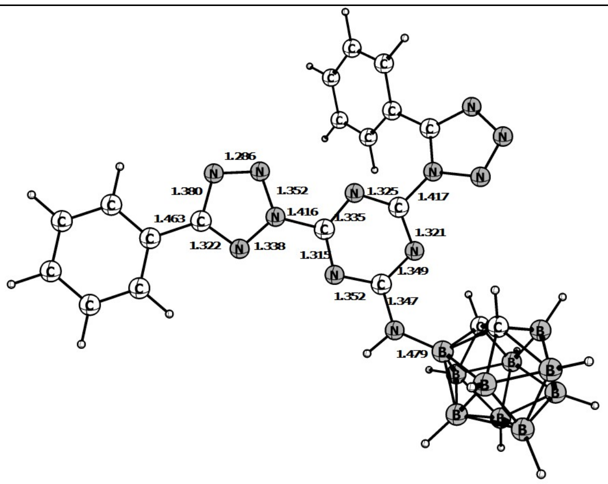
</td></tr>
</table>

Table S11. The calculated values of total energy and entropy of compounds **9-11**, **15-17**, **19** and **25** (6-311+G* basis set) in gas phase.

Compound	E_t, a.u.	E_{zpc}, a.u.	G, a.u.	S, a.u.
**9**	–1181.0509	–1180.7589	–1180.8105	0.0735
**10**	–1259.7142	–1259.3665	–1259.4221	0.0807
**11**	–1338.3563	–1337.9513	–1338.0110	0.0875
**15**	–1523.8202	–1523.5470	–1523.5996	0.0752
**16**	–1523.8626	–1523.5870	–1523.6405	0.0761
**17**	–1523.8877	–1523.6113	–1523.6637	0.0747
**19**	–1424.2702	–1423.8363	–1423.8949	0.0863
**25**	–1643.2666	–1642.8134	–1642.8794	0.0974

Table S12. Comparison of calculated and experimental EOF values of test molecules (kcal/mol).

Compound	$\Delta H_{calc.}$ (kcal/mol) Present calcultions	$\Delta H_{exp.}$ (kcal/mol) Literature data
o-carborane ($C_2H_{12}B_{10}$)	–26.4	–26.4±2.6
m-carborane ($C_2H_{12}B_{10}$)	–42.0	–42.6±3.4
1H-tetrazole	+80.0	+79.0±1.0
2H-tetrazole	+77.3	+77.3±1.0
5-methyl-1H-tetrazole	+71.7	+67.2±1.0
1-methyl-1H-tetrazole	+78.0	+74.2±1.0
TTZ	+161.8	+159.5[a]
TETZ	+190.3	+188.3[a]
TAAT	+442.2	+460.0[b]
TAH	+325.2	+336.8[b]
TAT	+258.1	+271.7[b]

[a] For compounds TTZ (1,2,4-triazolo-[4,3-b]-1,2,4,5-tetrazine), TETZ (tetrazolo-[1,5-b]- 1,2,4,5-tetrazine) data of the right column are taken from article T. Ewi et. al. [2].

[b] For compounds TAAT (4,4′,6,6′-tetra(azido)azo-1,3,5-triazine), TAH (2,5,8-tri(azido)-s-heptazine), TAT (2,4,6-tri(azido)-1,3,5-triazine), DiAT (3,6-di(azido)-1,2,4,5-tetrazine), which are leading HEDMs, the data of the right column are taken from article O. V. Dorofeeva et. al.[3].The experimentally evaluated EOF magnitude of TAAT [4] was found to be overestimated on 90 kcal/mol.

References

1. Turner, M. J.; McKinnon, J. J.; Wolff, S. K.; Grimwood, D. J.; Spackman, P. R.; Jayatilaka D.; Spackman, M. A. CrystalExplorer17 (2017). University of Western Australia.
2. Ewi, T.; Zhu, W.; Zhang, J.; Xiao, H. DFT study on energetic tetrazolo-[1,5-b]-1,2,4,5-tetrazine and 1,2,4-triazolo-[4,3-b]- 1,2,4,5-tetrazine derivatives. *J. Hazard. Mater*. **2010**, *179*, 581–590.
3. Dorofeeva, O.V.; Ryzhova, O.N.; Suntsova, M.A. Accurate Prediction of Enthalpies of Formation of Organic Azides by Combining G4 Theory Calculations with an Isodesmic Reaction Scheme. *J. Phys. Chem. A*. **2013**, *117*, 6835–6845.
4. Huynh, M.H.V.; Hiskey, M.A.; Pollard, C.J.; Montoya, D.P.; Hartline, E.L.; Gilardi, R. 4,4’,6,6’-Tetra-Substituted Hydrazo- and Azo-1,3,5-Triazines. *J. Energ. Mat*. **2004**, *22*, 217–229.
